# Cobalt(II) Complexes of 4′-Bromo-Fenamic Acid: Antioxidant Properties, Antibacterial Activity, and Interaction with DNA and Albumins

**DOI:** 10.3390/ijms26199787

**Published:** 2025-10-08

**Authors:** Georgios Malis, Christina N. Banti, Alexia Tialiou, Michael R. Reithofer, Antonios G. Hatzidimitriou, Sotiris K. Hadjikakou, Konstantina C. Fylaktakidou, George Psomas

**Affiliations:** 1 Laboratory of Inorganic Chemistry, Department of Chemistry, Aristotle University of Thessaloniki, GR-54124 Thessaloniki, Greece; 2 Department of Chemistry, University of Ioannina, GR-45110 Ioannina, Greece; 3 Institute of Inorganic Chemistry, Faculty of Chemistry, University of Vienna, Währinger Str. 42, 1090 Vienna, Austria; 4 Vienna Doctoral School in Chemistry (DoSChem), University of Vienna, Währinger Str. 42, 1090 Vienna, Austria; 5 Institute of Materials Science and Computing, University Research Centre of Ioannina (URCI), GR-45110 Ioannina, Greece; 6 Laboratory of Organic Chemistry, Department of Chemistry, Aristotle University of Thessaloniki, GR-54124 Thessaloniki, Greece

**Keywords:** fenamates, cobalt(II) complexes, antioxidant activity, antimicrobial activity, DNA binding, photocleavage of DNA, affinity for albumins

## Abstract

The reaction of 4′–bromo-fenamic acid, a bromo-derivative of fenamic acid (the scaffold of the fenamate non-steroidal anti-inflammatory drugs), with Co(II) in the absence or presence of various nitrogen-donor ligands yielded nine novel, neutral mononuclear Co(II) complexes. These complexes were characterized by physicochemical and spectroscopic techniques and single-crystal X-ray crystallography. The biological evaluation of the compounds focused on their antioxidant and antimicrobial efficacy, as well as their interaction with calf-thymus DNA, pBR322 plasmid DNA (in the absence or presence of diverse irradiations) and serum albumins. The complexes have shown significant antioxidant activity since they can scavenge 2,2′-azinobis-(3-ethylbenzothiazoline-6-sulfonic acid) radicals (up to 96.48 ± 0.07%) and reduce H_2_O_2_ (up to 96.93 ± 0.53%). Antimicrobial testing revealed that the complexes were more active than free 4′-bromo-fenamic acid with four of them classified as bactericidal agents against selected bacterial strains. The compounds can interact with calf-thymus DNA via intercalation, and the calculated DNA-binding constants are on the 10^6^ M^−1^ order. The plasmid DNA-cleavage ability of the compounds is strongly enhanced under UVA irradiation (photocleavage > 90%). In addition, the compounds can bind tightly and reversibly to serum albumins with binding constants in the 10^5^ M^−1^ range.

## 1. Introduction

Non-steroidal anti-inflammatory drugs (NSAIDs) are widely used in modern medicine for their anti-inflammatory, analgesic, and antipyretic properties and they account for nearly 10% of annually prescribed medications [1]. NSAIDs are essential for managing pain, fever and inflammation in both acute and chronic conditions, such as arthritis. Their mechanism of action primarily involves the inhibition of cyclooxygenase (COX) enzymes; these enzymes are key in the biosynthesis of prostaglandins which are mediators of inflammation and pain. Insertion of metal ions into organic drugs has often been shown to enhance the activity, and NSAID-based coordination compounds with first-row transition metals have yielded promising results [2]. Beyond their classical applications, increasing evidence supports the use of NSAIDs against cancer and Alzheimer’s disease, as antiviral and antibacterial agents, or even in treating major depressive disorder [3,4,5]. The therapeutic efficacy of NSAIDs is often limited due to gastrointestinal and cardiovascular side-effects, highlighting the need for ongoing research into more specific targets for the development of safer alternatives with reduced side-effects. In this regard, the development of NSAIDs with antioxidant properties is particularly attractive, as oxidative stress often accompanies long-term NSAID use [6,7].

Recent studies have identified NSAIDs as potential chemopreventive agents in several cancers, including breast cancer, where they interfere with signaling pathways involved in tumor progression [8,9]. Epidemiological and experimental studies paved the way for repurposing NSAIDs and their metal complexes in chemoprevention or chemotherapy [10,11]. In the case of melanoma which has a high mortality rate, malignancy and ability to acquire resistance to drugs, NSAIDs sensitized the suppression effect of mitogen-activated protein kinase inhibitors and efficiently inhibited various important neoplastic phenotypes [12,13]. Since melanoma progression is affected by light, both metallic and non-metallic photosensitizers have been studied as possible DNA-photocleavers or tools for photodynamic therapies (PDT) [14,15,16,17]. In this context, compounds combining anti-inflammatory and phototoxicity properties are especially appealing [18,19]. Furthermore, epidemiological studies concerning NSAIDs revealed their antibacterial potency, their impact on the treatment of Alzheimer’s disease [20] and their potential use as antiseptic and/or disinfectant in topical formulations [21].

**Figure 1:**
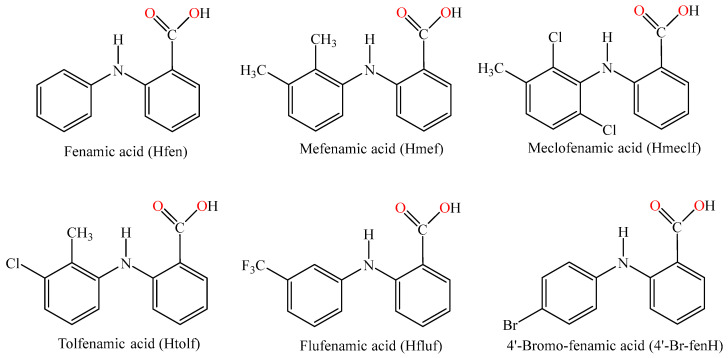
The structural formulas of fenamic acid (Hfen), and its derivatives: mefenamic acid (Hmef), tolfenamic acid (Htolf), meclofenamic acid (Hmeclf), flufenamic acid (Hfluf) and 4′-bromofenamic acid (4′-Br-fenH).

Fenamic acid (Hfen) is an anthranilic acid bearing a characteristic *N*-arylated scaffold. It belongs to the ‘fenamate family’ of NSAIDs, which includes mefenamic acid (Hmef), tolfenamic acid (Htolf), meclofenamic acid (Hmeclf), and flufenamic acid (Hfluf) (Figure 1). They bear a *N*-aryl scaffold substituted with one-to-three alkyl or halogen groups on each or combination of 2′-, 3′-, 4′- and 6′-positions on the aromatic ring. Fenamates’ primary use as agents against pain, arthritis and dysmenorrhea is based on their strong anti-inflammatory, analgesic, and antipyretic properties [22]. These drugs are commercially available, exhibit a wide range of biological activities with extended common use and may be recruited in diverse therapeutic areas including the treatment of cancer, neurodegenerative diseases and bacterial infections [23,24,25,26]. The coordination compounds of commercially available fenamate NSAIDs have consistently demonstrated better biological properties compared to free fenamic acids, including antioxidant, anti-inflammatory, antibacterial, antiproliferative, anti-tuberculosis and/or antifungal activities [27].

4′-Bromo–fenamic acid (4′-Br-fenH, Figure 1) is a mono-brominated derivative of Hfen. In the literature, 4′-Br-fenH has been reported as a key intermediate in the synthesis of heterocycles such as acridines and acridones [28,29,30,31], fused *N*-1-aryl-quinazolinones [32] and evodiamines [33,34]. In contrast, another study described the use of Br on 4′-Br-fenH as a leaving group (‘sacrificed’) to yield linked heterocycles of fenamic ethylesters [35]. To the best of our knowledge, no coordination compounds have ever been synthesized with this ligand. From a biological perspective, the *N*-anthraniloyl tryptamine derivative of 4′-Br-fenH has been found to be a better COX-2 inhibitor due to the Br-substituent when compared with electron donating substituents (Me, OMe, NMe_2_) [36]. 4′-Br-fenH has been found to exhibit antifungal [37] and antidiarrheal activity [38].

As a part of Bioinorganic Chemistry research, metal-based drugs have been long established in the treatment of various diseases for many decades [39]. A combination of metal ions with biologically active compounds including drugs often results in novel compounds with improved or modified activity due to synergetic effects, milder side-effects, differentiated mechanisms of action, and solubility changes [39,40,41,42]. Cobalt is a trace element that has a crucial role in medicine, due to its key presence in cobalamin (vitamin B_12_), an important vitamin participating in various neurological functions, DNA synthesis, and the formation of red blood cells. At the same time, cobalt deficiency can cause anorexia, chronic swelling or even anemia [43,44]. Recently, novel cobalt complexes have attracted attention for their potential anti-inflammatory, antiproliferative, antioxidant, antimicrobial, and antiviral activity [3,42,45,46]. Among them, Co(II) complexes with carboxylate NSAIDs as ligands have displayed promising antioxidant activity [47,48,49,50,51,52,53,54].

**Figure 2:**
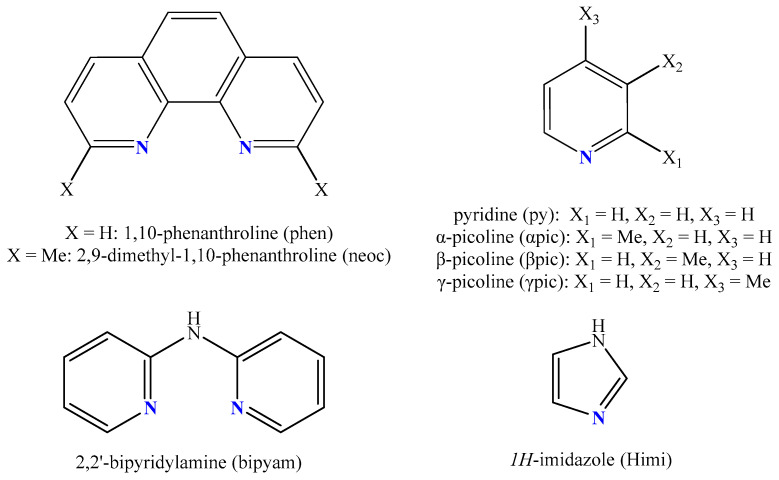
The structural formulas of the nitrogen-donor ligands: 1,10-phenanthroline (phen), 2,9-dimethyl-1,10-phenanthroline (neoc), pyridine (py), 2-methylpyridine (α-picoline, αpic), 3-methylpyridine (β-picoline, βpic), 4-methylpyridine (γ-picoline, γpic), 2,2′-bipyridylamine (bipyam) and *1H*-imidazole (Himi).

Given the significant biological activity of fenamates, the preparation and characterization of a synthetic bromo-derivative of fenamic acid, i.e., 4′-bromo-fenamic acid (4′-Br-fenH, Figure 1), is of particular interest for several reasons such as the: (i) biological activity of the free compound [37,38] and its derivatives [36], (ii) enhanced activity arising from the insertion of the halogen compared to the non-halogenated derivatives [19,55], (iii) DNA-photocleavage activity shown by *p*-halogenated anthranilic acid derivatives [17] due to the heavy atom effect of the halogen [56], (iv) absence of commercially available fenamates containing bromine atom(s), and (v) wide interest for coordination compounds with ligands containing the amino-benzoic scaffold [27]. Based on the enhanced biological activity exhibited by the reported Co(II)-NSAID complexes [47,48,49,50,51,52,53,54], and in order to explore how the insertion of the Br-group on the fenamic acid influences the biological profile of the free drug and its metal complexes, nine novel Co(II) complexes with 4′-Br-fenH were synthesized in the absence or presence of a variety of nitrogen-donors as co-ligands, including the *N*-donors *1H*-imidazole (Himi), pyridine (py), 2-methylpyridine (α-picoline, αpic), 3-methylpyridine (β-picoline, βpic), and 4-methylpyridine (γ-picoline, γpic), or the *N*,*N*′-donors 1,10-phenanthroline (phen), 2,9-dimethyl-1,10-phenanthroline (neocuproine, neoc), and 2,2′-bipyridylamine (bipyam) (Figure 2). The resultant coordination compounds, namely [Co(4′-Br-fen)_2_(MeOH)_4_]·2MeOH (complex **1**), [Co(4′-Br-fen)_2_(phen)(MeOH)_2_] (complex **2**), [Co(4′-Br-fen)_2_(neoc)(MeOH)_2_] (complex **3**), [Co(4′-Br-fen)_2_(bipyam)] (complex **4**), [Co(4′-Br-fen)_2_(Himi)_2_] (complex **5**), [Co(4′-Br-fen)_2_(py)_2_(MeOH)_2_] (complex **6**), [Co(4′-Br-fen)_2_(αpic)_2_(MeOH)_2_] (complex **7**), [Co(4′-Br-fen)_2_(βpic)_2_(MeOH)_2_] (complex **8**) and [Co(4′-Br-fen)_2_(γpic)_2_(MeOH)_2_] (complex **9**), were characterized with physicochemical (elemental analysis, molar conductivity measurements) and spectroscopic methods (FT-IR, MS and UV-vis). Additionally, the crystal structure of complex **1** was determined with single-crystal X-ray crystallography.

To determine and better understand the biological profile of the synthesized compounds, their antioxidant and antimicrobial activity, as well as their interactions with key biomolecules such as DNA and albumins, were evaluated to shed light on some issues related to the properties of NSAIDs. More specifically, the ability of the compounds to scavenge free radicals, such as 1,1-diphenyl-picrylhydrazyl (DPPH) and 2,2′-azinobis(3-ethylbenzothiazoline-6-sulfonic acid) (ABTS), and to reduce H_2_O_2_ was investigated as the measurement of the antioxidant activity. The antimicrobial activity of the compounds was evaluated against the Gram-negative strains *Pseudomonas aeruginosa* (*P. aeruginosa*) and *Escherichia coli Dh5a* (*E. coli*) and the Gram-positive ones *Staphylococcus epidermidis* ATCC^®^ 14990™ (*S. epidermidis*) and *Staphylococcus aureus* subsp. *aureus* ATCC^®^ 25923™ (*S. aureus*). The interaction of the compounds with calf-thymus (CT) DNA was studied with UV-vis spectroscopy, viscosity measurements, and fluorescence emission spectroscopy aiming to calculate the corresponding DNA-binding constant, and to assess DNA-interaction mode, the competition with ethidium bromide (EB) and the effect of temperature on this interaction. The interaction of the compounds with supercoiled circular pBR322 plasmid DNA (pDNA) was focused on evaluating their ability to cleave pDNA and the effect of UVA, UVB or visible light on cleavage. The affinity of the compounds for both bovine (BSA) and human serum albumins (HSA) was monitored with fluorescence emission spectroscopy purposing the calculation of the corresponding binding constants and the determination of the albumin-binding site.

## 2. Results and Discussion

### 2.1. Synthesis and Characterization of the Compounds

**Scheme 1:**
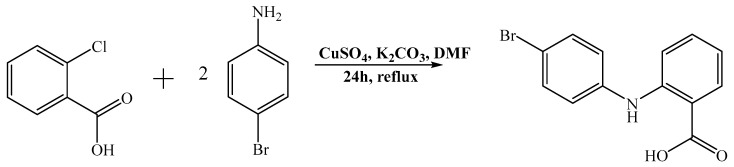
Synthesis of compound 4′-Br-fenH using 2-chlorobenzoic acid (1 eq), 4-bromoaniline (2 eq), CuSO_4_ (catalytic), K_2_CO_3_ (0.5 eq), DMF, reflux, 24 h, 77% yield.

4′-Br-fenH was synthesized (Scheme 1) using a modified literature procedure [57,58]. The obtained yield (77%) was higher compared to the yields obtained at reactions performed thermally (74%) [57]. 4′-Br-fenH was characterized with ^1^H NMR and IR spectroscopies (Figures S1 and S2) and the obtained data were in agreement with the literature [28].

The aerobic reaction of deprotonated 4′-Br-fenH with a methanolic solution of CoCl_2_·6H_2_O in a 1:2 Co^2+^:(4′-Br-fen)^−1^ ratio in the absence or presence of the corresponding nitrogen-donor ligands yielded complexes **1**–**9** which were characterized with IR and UV-vis spectroscopies, mass spectrometry (HRMS or ESI MS), and single-crystal X-ray crystallography (for complex **1**). The proposed formulas of the complexes are based on the results of elemental analysis and mass spectrometry (in the mass spectra, fragments close to the expected molecular ions were observed, Figure S3). The complexes are stable in the air and soluble in MeOH, EtOH, and DMSO, but partially soluble or insoluble in H_2_O. According to the molar conductivity values (Λ_M_ values ranging 10–15 S∙cm^2^∙mol^−1^ for a 1 mM DMSO solution), the complexes are non-electrolytes in DMSO solution [59], thus they do not dissociate in solution maintaining their structure.

In the IR spectra of the complexes, bands attributed to characteristic groups of the ligands were monitored to determine the presence and the coordination mode of the ligands (Figure S2). More specifically, for all complexes **1**–**9**, two characteristic bands of the carboxylic group of the 4′-Br-fen^−1^ ligand were observed in the range 1579–1583 cm^−1^ and 1384–1400 cm^−1^, attributed to the antisymmetric (ν_asym_(COO)) and the symmetric (ν_sym_(COO)) stretching vibrations, respectively, of the carboxylato group. The parameter Δν(COO) (defined as Δν(COO) = ν_asym_(COO) − ν_sym_(COO); values higher than that of the corresponding of salt of carboxylate ligand may reveal a monodentate coordination mode, while lower values may indicate a bidentate chelating/bridging fashion [60,61]) is in the range 183–197 cm^−1^, suggesting an asymmetric coordination of the carboxylato group of 4′-Br-fen^−1^ ligands [60,61]. In the IR spectra of complexes **2**–**9**, characteristic bands attributed to the out-of-plane *ρ*(C-H) of the nitrogen-donor co-ligands are found in region 695–770 cm^−1^ verifying the coordination of these co-ligands in the complexes [60]. Similar IR findings were reported for the Co(II)-NSAID complexes found in the literature [47,48,49,50,51,52,53,54].

In the visible region of the UV-vis spectra of most complexes **1**–**9** recorded in DMSO solution, one or two low-intensity bands were observed in region 470–656 nm and can be attributed to d-d transitions (^4^Τ_1g_(F)→^4^A_2g_ and ^4^Τ_1g_(F)→^4^T_1g_(P)), characteristic for octahedral Co(II) complexes [62]. Similar bands were also observed for previously reported Co(II)-NSAID complexes [47,48,49,50,51,52,53,54]. In the UV region of the spectra, one or two bands attributed to intra-ligand transitions were observed in the range 268–364 nm. In addition, the UV-vis spectra of the complexes were also recorded in the presence of buffer solution (150 mM NaCl and 15 mM trisodium citrate, pH = 7, which is used in the DNA/albumin interaction studies). The similarity of the spectra (representatively shown in Figure S4) with those recorded in DMSO may suggest, in combination with the molar conductivity results, that the complexes retain their pharmacophore structure in these solution mixtures.

### 2.2. Structure of the Complexes

Among the nine novel Co(II) complexes studied herein, single-crystals suitable for X-ray crystallography structural determination were obtained only for complex 1. The structural characterization of complexes **2**–**9** was performed based on derived experimental data and after comparison with the literature.

#### 2.2.1. Crystal Structure of [Co(4′-Br-fen)_2_(MeOH)_4_]·2MeOH (Complex **1**)

**Figure 3:**
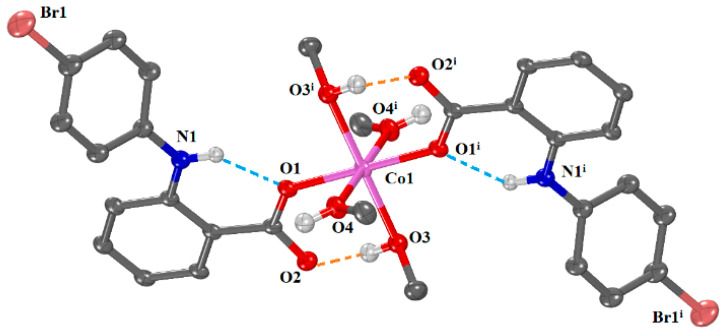
Molecular structure of complex **1** [Co(4′-Br-fen)_2_(MeOH)_4_] (symmetry code: (i) −*x* + 2, −*y*, −*z* + 2). Aromatic and methyl hydrogen atoms and solvate methanol molecules are omitted for clarity reasons. The light-blue and orange dashed lines represent the intra-ligand and intramolecular, respectively, hydrogen bonds.

The molecular structure of complex **1** is shown in Figure 3, and selected bond lengths and angles are summarized in Table 1. The complex **1** crystallized in triclinic crystal system and *P*ī space group. Two solvate methanol molecules were also found in the structure.

The structure of the complex is centrosymmetric with the cobalt(II) ion being on the symmetry center coordinated to two 4′-Br-fen^−1^ and four methanol ligands, all related in pairs by the inversion center. The complex is mononuclear with each deprotonated 4′-Br-fenamato ligand being monodentately bound to the cobalt(II) ion through a carboxylato oxygen; therefore, the cobalt atom is six-coordinate with a CoO_6_ coordination sphere and an octahedral geometry. Among the Co-O bond lengths, the Co-O_carboxylate_ are the shortest, having value (Co(1)-O(1) = 2.0391(19) Å), and the Co-O_methanol_ being the longest and measured as Co(1)-O(3) = 2.0966(19) Å and Co(1)-O(4) = 2.1253(17) Å. Taking into consideration the differences in the Co(1)-O bond lengths and the angles around Co(1) (in the range 87.50(8)–92.50(8)°), the octahedral coordination geometry displays distortion. The arrangement of the 4′-Br-fenamato ligands around the Co(II) ion is similar to that observed in the crystal structures diverse metal(II)-fenamato complexes, such as [Co(mef)_2_(MeOH)_4_] [48], [Co(nif)_2_(MeOH)_4_] [53], [Zn(nif)_2_(MeOH)_4_] [63], and [Mn(nif)_2_(MeOH)_4_] [64] (Hmef and Hnif are the NSAIDs mefenamic acid and niflumic acid, respectively).

A series of hydrogen bonds further stabilize the structure of complex **1**. In particular, intraligand H-bonds are developed between the coordinated carboxylato oxygen O1 and the amino hydrogen H11 of 4′-Br-fenamato ligands and intramolecular H-bonds are developed between the non-coordinated carboxylato oxygen O2 of 4′-Br-fenamato ligands and the hydroxy hydrogen H165 of MeOH ligands. Furthermore, intermolecular H-bonds between hydroxy hydrogen H166 of solvate MeOH molecules and non-coordinated 4′-Br-fen carboxylato oxygen O2 of adjacent molecules contribute to further stabilization of the structure (Table S2).

#### 2.2.2. Proposed Structures for Complexes **2**–**9**

Based on the MS, IR and UV-vis spectral data, and molar conductivity measurements, as well as a comparison to the literature, the structures for complexes **2**–**9** may be proposed. All complexes are non-electrolytes and contain deprotonated 4′-Br-fen^−1^ ligands and the corresponding nitrogen-donor ligands.

Based on mass spectra, complexes **2** and **3** are expected to have the general formula [Co(4′-Br-fen)_2_(*N*,*N*′-donor)(MeOH)_2_], i.e., [Co(4′-Br-fen)_2_(phen)(MeOH)_2_] and [Co(4′-Br-fen)_2_(neoc)(MeOH)_2_], respectively. The 4′-Br-fen^−1^ ligands are expected to bind monodentately to the Co(II) ion; the Co(II) coordination sphere is completed with two nitrogen atoms of the corresponding *N*,*N*′-donor ligands phen and neoc and two oxygen atoms from the methanol ligands (Figure S5). Similar arrangement around Co(II) in Co(II)-fenamato complexes was reported in the structures of complexes [Co(mef-O)_2_(bipy)(MeOH)_2_] [48] and [Co(tolf-O)_2_(bipy)(MeOH)_2_] [52] (Htolf is the NSAID tolfenamic acid).

According to mass spectra, the formula [Co(4′-Br-fen)_2_(bipyam)] is suggested for complex **4**. In this case, the coordination sphere of the six-coordinate Co(II) ion is expected to consist of four oxygen atoms from two 4′-Br-fenamato ligands bound in an asymmetric bidentate chelating mode and two nitrogen atoms from bipyam ligand (Figure S5). Such arrangement is similar to that previously reported for complexes [Co(tolf-O,O′)_2_(bipyam)] [52], [Co(fluf-O,O′)_2_(bipyam)] and [Co(mef-O,O′)_2_(bipyam)] [53] (Hfluf is the NSAID flufenamic acid).

Complex **5** is suggested to bear the formula [Co(4′-Br-fen)_2_(Himi)_2_] and have a similar structure to complexes [Co(dicl)_2_(Himi)_2_] (Hdicl is the NSAID diclofenac) [50], [Co(tolf)_2_(Himi)_2_] and [Co(mef)_2_(Himi)_2_] [49]. For this reason, an asymmetric bidentate chelating mode for two 4′-Br-fenamato ligands may be suggested leading to a ‘pseudo-octahedral’ geometry around Co(II) ion with ‘4 + 2’ coordination number (Figure S5).

Complexes **6**–**9** are expected to bear the general formula [Co(4′-Br-fen)_2_(*N*-donor)_2_(MeOH)_2_] where *N*-donor is py, αpic, βpic and γpic, respectively. Two O atoms of the monodentately bound 4′-Br-fenamato ligands, two N atoms from two pyridine/picoline ligands and two O atoms from methanol ligands constitute the coordination sphere of the Co(II) ions being in a distorted octahedral geometry (Figure S4), which is similar to that reported for complexes [Co(nap)_2_(py)_2_(H_2_O)_2_] [47], [Co(meclf)_2_(py)_2_(H_2_O)_2_] [49] and [Co(dicl)_2_(py)_2_(H_2_O)_2_] [50] (Hnap and Hmeclf are the NSAIDs naproxen and meclofenamic acid, respectively).

### 2.3. Antioxidant Activity of the Compounds

Free radicals are species with unpaired electron(s) and play a significant role in the inflammatory process. The transfer of unpaired electron(s) to adjacent molecules can initiate chain reactions which may induce various detrimental effects, including swelling, inflammation, and potentially cancer [65]. In general, NSAIDs can function in diverse ways, either by inhibiting the formation of free radicals or by scavenging these reactive species. Therefore, such compounds exhibiting antioxidant properties can be crucial in managing inflammation, paving the way for the development of novel pharmaceuticals. Within this context, the antioxidant activity of compounds under study was evaluated for their ability to scavenge DPPH and ABTS radicals and to reduce H_2_O_2_, and was compared with appropriate reference compounds, i.e., nordihydroguaiaretic acid (NDGA), butylated hydroxytoluene (BHT), 6-hydroxy-2,5,7,8-tetramethylchroman-2-carboxylic acid (trolox), and L-ascorbic acid [66,67]. The results of the antioxidant activity of all the synthesized compounds are shown in Table 2.

The evaluation of DPPH radical scavenging is a process based on electron transfer that produces a violet colored methanolic solution. In the presence of an antioxidant agent, the free DPPH radical is reduced, resulting in a colorless solution [68]. This scavenging ability can be linked with a possible antiaging, anti-inflammatory and possible anticancer activity of the compounds [69]. As shown in Table 2, among the compounds, only complexes **2**, **4** and **9** exhibit a measurable activity which is time-dependent, yet significantly lower than reference compounds BHT and NDGA. Among these three active compounds, complex **9** is by far the most active DPPH-scavenger (DPPH% up to 35.65 ± 1.05%) being within the range reported for Co(II)-NSAID complexes (DPPH% = up to 42.4%) [47,48,49,50,51,53,54].

To evaluate the total oxidation capacity of each compound, ABTS radicals are used as a marker [70]. The ABTS-scavenging assay monitors the discoloration of a dark green solution containing the cationic radical ABTS^•+^ induced by hydrogen-donating antioxidants. As displayed in Table 2, all complexes present high ABTS-scavenging ability, close to that of trolox, with complex **9** being the best ABTS-scavenger (ABTS% = 96.48 ± 0.07%). It is worth noting that even though the free ligand is practically inactive, when coordinated with Co(II), a marked increase in its ABTS-scavenging ability occurs, which is in accordance with the enhanced activity shown by the metal-NSAID complexes when compared to free NSAID. Regarding the nitrogen-donor ligands, the use of the *N*-donor co-ligands (in complexes **5**–**9**) results in higher activity than that of *N*,*N*′-donors (in complexes **2**–**4**). Most of the Co(II)-NSAID complexes reported in the literature exhibited such high ABTS-scavenging activity (ABTS% = 21.4–99.0%) [47,48,49,50,51,53,54].

The interaction between the compounds and H_2_O_2_ was also studied. Hydrogen peroxide functions as an oxidizing agent [71], able to produce hydroxyl radicals when interacting with transition metals; thus compounds able to act as hydroxyl radical scavengers or H_2_O_2_ reductants can inhibit the production of reactive oxygen species (ROS) and offer protection from oxidative stress [72]. All compounds, including free 4′-Br-fenH, can greatly reduce H_2_O_2_ with values higher than that of reference compound L-ascorbic acid (Table 2). Complex **3** induces by far the highest percentage of H_2_O_2_ reduction (H_2_O_2_%= 96.93 ± 0.53%). Such high activity towards H_2_O_2_ or hydroxyl radicals was also reported for a series of Co(II)-NSAID complexes (H_2_O_2_% = 69.5–98.6%) [47,48,49,50,53,54].

In total, the compounds were practically inactive towards DPPH radicals except for complex **9,** which presented moderate activity. The behavior of the compounds towards ABTS radicals and H_2_O_2_ was significant and comparable to similar reported Co(II)-NSAID complexes [47,48,49,50,51,53,73]. Complexes **7**–**9** were the most active among the compounds towards ABTS radicals, approaching the activity of the reference compound trolox. All compounds studied herein were more active towards hydrogen peroxide than the reference compound L-ascorbic acid with [Co(4′-Br-fen)_2_(neoc)(MeOH)_2_] (complex **3**) being the most active compound.

### 2.4. Antibacterial Activity

#### 2.4.1. Antibacterial Effect of Compounds on the Growth of Microbial Strains

The antimicrobial activity of 4′-Br-fenH and its complexes **1**–**9** was evaluated against two Gram-negative strains (*P. aeruginosa* and *E. coli*) and two Gram-positive ones (*S. epidermidis* and *S. aureus*) by means of minimum inhibitory concentration (MIC), minimum bactericidal concentration (MBC) and agar diffusion method—inhibition zone (IZ). MIC is defined as the minimum concentration required to inhibit bacterial growth, while MBC is the lowest concentration of an antibacterial agent needed to eliminate 99.9% of the bacterial inoculum. IZ refers to the area (in mm) in the media where bacteria are unable to grow due to the presence of a drug that inhibits their growth [74,75]. The quinolone antibacterial drug ciprofloxacin was used as the positive control [76]; the IZ diameters developed by ciprofloxacin were 35.5 ± 0.6 mm, 33.0 ± 0.8 mm, 39.2 ± 0.9 mm and 30.5 ± 0.6 mm for *P. aeruginosa*, *E. coli*, *S. epidermidis* and *S. aureus*, respectively.

The MIC values of compounds **1**–**9** against Gram-negative microbes ranged from 20.0 μM to over 250 μM, and from 50.3 to over 250 μM against Gram-positive microbes (Table 3). Free 4′-Br-fenH exhibited low antibacterial activity, with MIC values higher than 200 μΜ.

The compounds were active against *E. coli*, and complexes **3** and **7** exhibited the strongest activity, both showing a MIC value of 20 μM. However, free 4′-Br-fenH and its complexes **1**–**9** were inactive against *P. aeruginosa*, with MIC values exceeding 250 μM. For Gram-positive bacteria, the lowest MIC values were observed for complex **2** (MIC = 60.5 μM) against *S. epidermidis* and complex **3** (MIC = 50.3 μM) against *S. aureus*. It may be noted that complexes **2** and **3** (bearing the *N*,*N*′-donors phen and neoc, respectively) were among the most active compounds between the compounds studied herein. Such enhanced activity of compounds bearing chelating *N*,*N*′-donor co-ligands is expected since the bidentate chelating ligands lead to higher activity than monodentate ligands, according to the chelation theory [77,78].

#### 2.4.2. Minimum Bactericidal Concentration Testing

**Figure 4:**
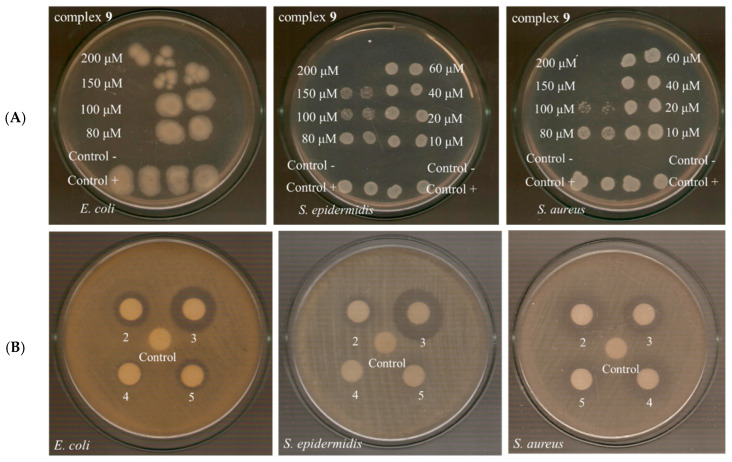
(**A**) Minimum bactericidal concentration of complex **9** against *E. coli*, *S. epidermidis* and *S. aureus*. (**B**) IZs which are developed in agar plates of *E. coli*, *S. epidermidis* and *S. aureus* by complexes **2**–**5** at 1 mM.

The MBC values found for complexes **1**–**9** ranged from 75 μM to over 250 μM (Table 3, Figure S6). The MBC/MIC ratio was used to categorize antibacterial agents as either bacteriostatic or bactericidal; agents with MBC/MIC value ≤ 2 were classified as bactericidal ones, indicating that they eliminated 99.9% of the microorganisms, and agents with MBC/MIC ≥ 4 were classified as bacteriostatic as they only inhibit but did not kill the microorganism [74,75,79]. The MBC/MIC value against *E. coli* could only be determined for complexes **4**, **5**, and **8**, classifying them as bactericidal agents. Additionally, complex **9** (Figure 4A) was classified as bactericidal against both *S. epidermidis* and *S. aureus*, while complex **8** was bactericidal only against *S. aureus* (Table 3).

#### 2.4.3. Determination of the IZ Through Agar Disc-Diffusion Method

The antimicrobial activity of the compounds was also tested using the agar diffusion assay (Table 3, Figure S7). Sterilized paper discs, 9.0 mm in diameter, were soaked in a solution of the compounds (1 mM). The discs were placed on agar plates, and they were incubated at 37 °C for 20 h. The IZs values of 4′-Br-fenH and its complexes **1**–**9** were in the range 9.0–19.5 mm against all tested bacterial strains. Free 4′-Br-fenH possessed no activity towards any of the bacterial strains. Complex **3** showed the highest IZ values, i.e., 16.1 ± 1.2, 19.5 ± 0.9, 16.7 ± 1.3 mm against *E. coli*, *S. epidermidis* and *S. aureus*, respectively. In most cases, the complexes exhibited high antibacterial activity, displayed low MIC values and simultaneously showed a large inhibition zone.

Based on the size of the inhibition zone, microbes could be classified into three categories: susceptible (IZ ≥ 17 mm), intermediate (13 ≤ IZ ≤ 16 mm), and resistant (IZ ≤ 12 mm), [75,80]. The bacteria used in this study were generally considered resistant (Table 3), except for complex **3**, which was susceptible to *S. epidermidis* and intermediate against *E. coli* and *S. aureus* (Figure 4B). The *P. aeruginosa* strain was resistant to all complexes.

### 2.5. Interaction of the Complexes with CT DNA

As a means to investigate the biomedical applications of the compounds, it is important to study the binding affinity of the compounds with DNA. As is known, metal complexes bind to DNA via covalent bond(s) (replacement of a labile ligand of the coordination compounds by a constituent of DNA, usually nitrogen atom(s) of the DNA-bases) or through non-covalent interactions (intercalation, electrostatic interactions and groove-binding) [81,82]. Furthermore, the metal complexes may present nuclease-like activity by inducing cleavage to the DNA-helix. To elucidate the nature of the interaction of herein compounds with CT DNA, UV-vis spectroscopy, viscosity measurements, and ethidium bromide displacement assays were employed.

#### 2.5.1. Interaction of the Complexes with CT DNA Studied with UV-Vis Spectroscopy

UV-vis spectroscopic analysis is commonly used as an initial evaluation of the interaction of complexes with DNA. Within this context, the UV-vis spectra of the compounds were recorded in the presence of incrementally increased amounts of CT DNA (Figure S8). The intra-ligand bands of the complexes showed a slight hyperchromism or hypochromism often with a bathochromic shift (Table 4). Such alterations confirm the interaction between the compounds and CT DNA, suggesting the formation of a new DNA-complex adduct which offers a stabilization of the interacting system [81,83]. Nevertheless, a clear interaction mode between the compounds and CT DNA cannot be safely concluded, necessitating the employment of viscosity measurements and competitive studies with EB.

With the Wolfe–Shimer equation (Equation (S1)) [84] and the plots of [DNA]/(ε_A_ − ε_f_) versus [DNA] (Figure S9), the DNA-binding constants (K_b_) of the compounds were calculated. Apart from complex **5,** all of the complexes had higher K_b_ values than free 4′-Br-fenH (Table 4), of the 10^5^–10^6^ M^−1^ magnitude. Additionally, the K_b_ values of all complexes **1**–**9** were higher than that of the classical intercalator EB (1.23 × 10^5^ M^−1^) [85]. Compared to similar Co(II) complexes with various NSAIDs (K_b_ = 9.65 × 10^3^–9.41 × 10^6^ M^−1^) [47,48,49,50,51,52,53,73,86,87,88], the synthesized complexes exhibited similar or even higher K_b_ values, with complexes **6**–**8** bearing significantly high K_b_ values (higher than 1 × 10^6^ M^−1^, Table 4), that rank them in the tightest DNA-binder among the Co(II)-NSAID complexes.

#### 2.5.2. CT DNA Viscosity Measurements

**Figure 5:**
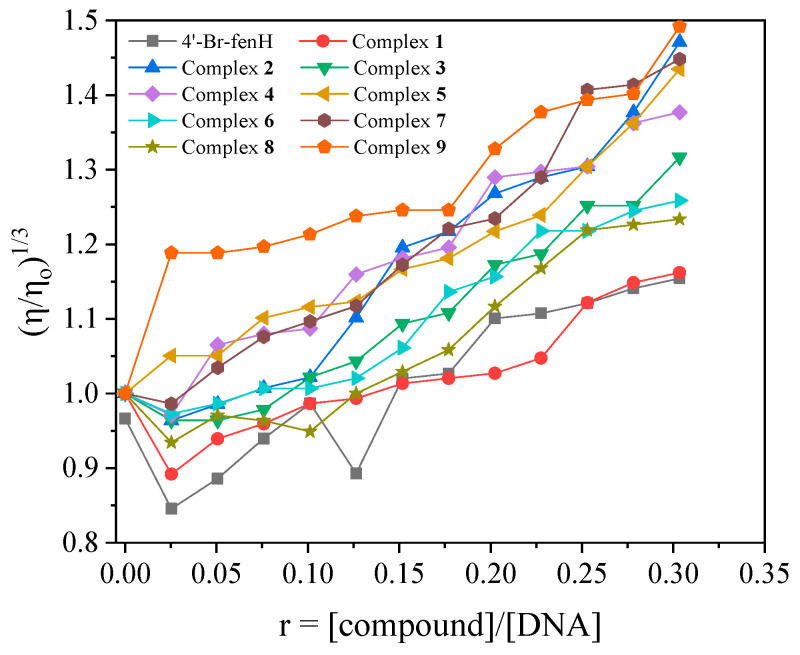
Relative viscosity (η/η_0_)^1/3^ of CT DNA (0.1 mM) in buffer solution (150 mM NaCl and 15 mM trisodium citrate at pH 7.0) in the presence of the compounds in increasing amounts (r = [compound]/[DNA]).

DNA-length changes occurring during the interaction of compounds with DNA can affect DNA-viscosity since they are proportional. More specifically, practically stable or slightly decreased DNA-viscosity usually indicates electrostatic interactions of the compounds to the outer space of DNA or in the grooves of DNA which cause the DNA-helix to bend, reducing its length [89]. On the other hand, if the interaction of a compound takes place through intercalation, an increase in DNAviscosity is noticed, due to the lengthening of the DNA-helix caused by the separation of the DNA-base pairs to accommodate the intercalating compound. In the case of DNA-cleavage, shorter fragments of DNA will result in a significant decrease in DNA-viscosity [89]. In the current study, the viscosity of a CT DNA solution (0.1 mM) was monitored in the presence of incrementally increasing concentrations of each compound (Figure 5). For most complexes, viscosity initially decreased slightly (r ≤ 0.1) and then increased markedly. Such behavior is consistent with the outer-surface approach of CT DNA followed by classic intercalation.

#### 2.5.3. Competitive Studies with EB

**Figure 6:**
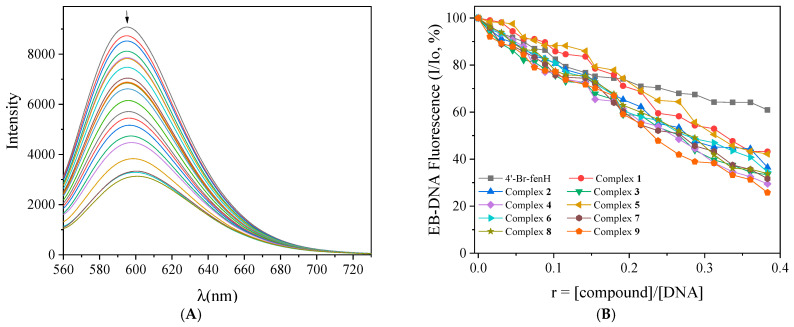
(**A**) Fluorescence emission spectra (λ_excitation_ = 540 nm) for EB-DNA conjugate ([EB] = 40 µM, [DNA] = 40 µM) in buffer solution (150 mM NaCl and 15 mM trisodium citrate at pH = 7.0) in the absence and presence of increasing amounts (up to r = [compound]/[DNA] = 0.38) of complex **9**. The arrow shows the changes of intensity upon increasing amounts of the complex. (**B**) Plot of relative EB-DNA fluorescence emission intensity at λ_emission_ = 594 nm (I/I_0_, %) versus r (r = [compound]/[DNA]) in the presence of 4′-Br-fenH and its complexes **1**–**9** (up to 60.9% of the initial EB-DNA fluorescence for 4′-Br-fenH, 53.2% for complex **1**, 36.5% for complex **2**, 33.4% for complex **3**, 29.5% for complex **4**, 42.2% for complex **5**, 34.5% for complex **6**, 31.7% for complex **7**, 33.8% for complex **8**, and 25.8% for complex **9**).

EB is a DNA-intercalator through the insertion of its planar phenanthridine ring in-between two adjacent bases of the DNA double helix leading to the formation of a fluorescent EB-DNA adduct with an intense emission band at λ_max_ = 592–594 nm, upon excitation at 540 nm [90]. A possible quenching of this fluorescence upon the addition of a compound able to intercalate into DNA-bases indicated that the tested compound displaced EB from EB-DNA due to their competition for the DNA-intercalation sites [90]. For this purpose, the fluorescence emission spectra of a buffer solution of EB (40 μM) and CT DNA (40 μM) (pretreated for 1 h) were recorded (with λ_excitation_ = 540 nm) in the presence of increasing amounts of the compounds and the fluorescence emission intensity of the EB-DNA band was monitored (Figure S10 and representatively shown for complex **9** in Figure 6A). A significant quenching of the fluorescence EB-DNA emission band (Figure 6B) was noticed (up to 74.21% of the initial fluorescence, Table 5) which can be attributed to possible displacement of EB from EB-DNA by the compounds.

The Stern–Volmer constants (K_SV_) were calculated with the Stern–Volmer equation (Equation (S2)) [90] and the corresponding plots (Figure S11). Furthermore, the quenching constants (K_q_) for the compounds are calculated with Equation (S3) applying the value of 23 ns as the fluorescence lifetime of EB-DNA system (τ_0_) [91]. All compounds exhibit high K_SV_ values (Table 5), with the complexes having higher values than that of the free 4′-Br-fenH, and complex **4** having the highest value (=1.52(±0.05) × 10^5^ M^−1^). The K_q_ values of all compounds are significantly higher than 10^10^ M^−1^s^−1^, indicating the presence of a static quenching mechanism, which verifies the formation of a new DNA-compound adduct because of the displacement of EB [90].

#### 2.5.4. Effect of Temperature on the Interaction of the Complexes with CT DNA

**Figure 7:**
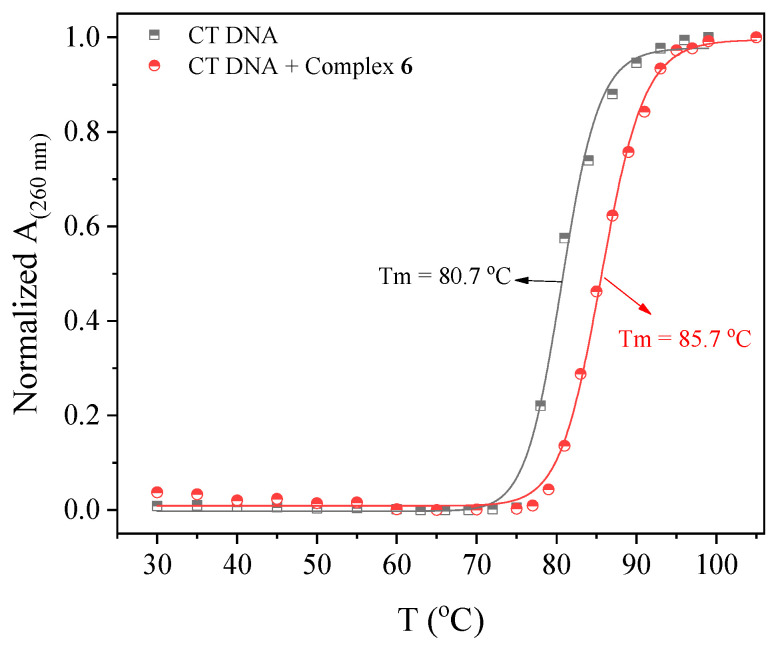
Thermal melting profile of CT DNA in the absence or the presence (compound:DNA ratio = 1:50) of complex **6**.

The thermal denaturation of DNA in the presence of the compounds was studied with UV-vis spectroscopy in order to study the effect of temperature on their interaction. The UV spectra of a CT DNA solution (1.25 × 10^−4^ M) in the presence of the compounds in a 1:50 compound:DNA ratio were recorded for increasing temperatures and the changes of the DNA UV-band with λ_max_ at 260 nm were monitored versus temperature (Figure S12). The DNA-melting temperature (T_m_), i.e., the temperature at which half of double-stranded DNA is denatured to single-stranded DNA (DNA-duplexes are “melted”), was determined via a sigmoidal fitting of the experimental data. As is known, compounds that bind to DNA in an intercalation mode offer stabilization of the DNA double-helix, inducing an increase in the T_m_ of DNA (usually by ~5 °C), while groove-binders do not change practically the T_m_ of the DNA duplex [92,93]. In the presence of complexes **1**–**9**, an increase in T_m_ of CT DNA from 80.7 °C to 83.5–87.7 °C (Figure 7, Table 4) is observed (i.e., ΔT_m_ = 2.8–7.0 °C) revealing the stabilization of CT DNA from the complexes as a result from an intercalative interaction, while the presence of 4′-Br-fenH induces a lower (<2 °C) increase of T_m_ (ΔT_m_ = 1.9 °C) revealing a stabilization probably from an external (non-intercalative) interaction.

#### 2.5.5. Thermodynamic Parameters of the Interaction of the Complexes with CT DNA

The most common interaction forces developed between compounds and DNA are hydrophobic forces, electrostatic interactions, van der Waals interactions and hydrogen bonds [94]. In the literature, the diverse combinations of the signs of the values calculated for the changes of enthalpy (ΔH) and entropy (ΔS) are related to the different types of this interaction. More specifically, positive values of both ΔH and ΔS (ΔH > 0 and ΔS > 0) are indicative of the presence of hydrophobic forces, negative values of both ΔH and ΔS (ΔH < 0 and ΔS < 0) are found for van der Waals interactions, while the case of ΔH < 0 and ΔS > 0 is consistent with electrostatic interactions [94].

In order to obtain deeper insight into the possible interaction forces developed between the compounds and CT DNA, the UV-vis spectroscopic titration studies in the presence of CT DNA were performed for three different temperatures (295 K, 303 K and 310 K) and the corresponding K_b_ values were also determined (Table S3). The changes of enthalpy (ΔH) and entropy (ΔS) were calculated from the van’t Hoff equation (Equation (S4)) and ΔG was obtained from the Gibbs–Helmholtz equation (Equation (S5)).

It may be noted that the increase in temperature resulted in higher K_b_ values for the complexes (Table S3). From the plots of ln(K_b_) versus (1/T) for the compounds (−ΔH/R is the slope and ΔS/R is the intercept of the fitting line (R is the universal gas constant) in Figure S13), the corresponding ΔH and ΔS values are calculated (Table S3). For the complexes, the positive values of ΔH and ΔS suggest the development of hydrophobic forces between them and CT DNA, stabilized by π-π stacking interactions which are explained from the existence of intercalative interaction [95]. Additionally, the negative ΔG values for all compounds indicate that their interaction with CT DNA is spontaneous [94,95,96].

### 2.6. (Photo)Cleavage of pBR322 Plasmid DNA

As a continuation of interaction studies with CT DNA, the ability of the compounds to damage plasmid DNA both in the presence and absence of irradiation was explored. For this purpose, the compounds (500 µM) were combined with pBR322 DNA in a tris buffer solution (25 μM, pH 6.8), ensuring that DMSO in the final mixture did not exceed 10% *v*/*v*. The effect of the compounds on pDNA was evaluated, after incubating the samples at 37 °C, with the results analyzed by gel electrophoresis on 1% agarose stained with EB in the absence or presence of UV-B (irradiation at 312 nm for 30 min) or UV-A (irradiation at 365 nm for 120 min) or visible light (irradiation for 120 min). During electrophoresis, the supercoiled pDNA appears as Form I in the gel. The interaction of the compounds with pDNA resulted in three forms: Form I (supercoiled pDNA), Form II (relaxed pDNA) induced from single-stranded (ss) damage, and Form III (linear pDNA) induced from double-stranded (ds) damage. The extent of pDNA-damage is assessed by calculating the percentages ss% and ds% with Equations (S6) and (S7) [97].

**Figure 8:**
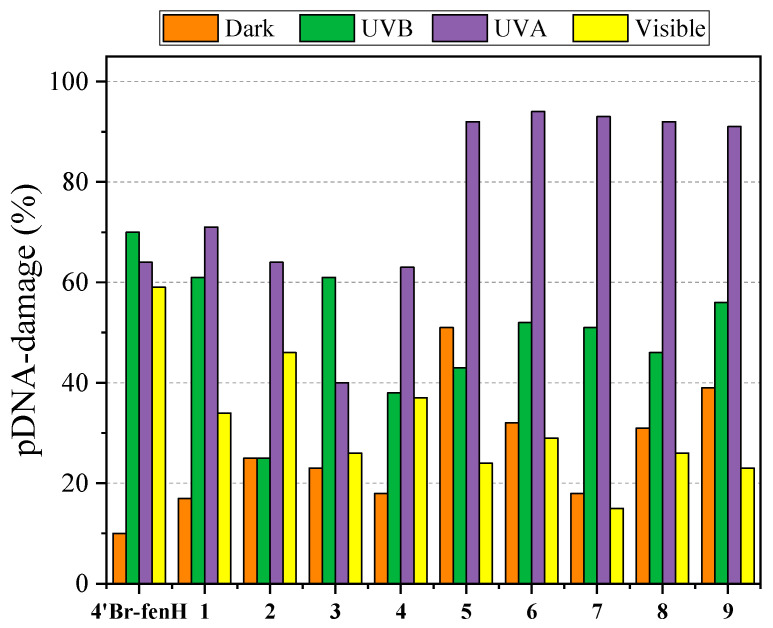
Overall percentage of damage induced by the compounds to plasmid DNA (pDNA-damage, in %) in the absence (dark) or presence of UVB, UVA, and visible irradiation.

The reaction mixtures of pDNA and the compounds were incubated in the dark for 150 min and then analyzed by 1% agarose gel electrophoresis with EB-staining (Figure S14). In the absence of light, all compounds (500 μM) converted supercoiled pDNA, albeit at a low percentage, into relaxed circular DNA (form II) by inducing single-strand (ss) breaks. In addition, low-percentage double-strand (ds) breaks were observed for complexes **2**, **3**, and **9**. The overall pDNA damage induced by the compounds was low-to-moderate with complex **5** inducing the highest overall pDNA-damage (~51%) among the compounds (Figure 8).

The exposure of pDNA-compounds to radiation resulted in more active compounds. After exposure for 30 min to UVB radiation, where the highest absorption is observed, the compounds become more active resulting in ss nicks (Figure S15). 4′-Br-fenH was active, provoking pDNA-damage of 70% (Figure 8). The overall pDNA-damage induced by the complexes after exposure to UVB radiation resulted in enhanced pDNA-damage in comparison to the absence of radiation (Figure 8).

The highest activity of the compounds was observed after exposure to UVA radiation, which induced more DNA-fragments (Figure S16) and much higher overall DNA-damage. The lower energy of UVA compared to UVB allowed more extensive irradiation periods (120 min) which seemed to facilitate photocleavage of the compounds, although their UV-vis absorption was much less efficient than in UVB. This was observed more evidently for complexes **5**–**9** (containing a *N*-donor co-ligand) where almost all pDNA was consumed, almost doubling the damage (Figure 8). In addition, for most complexes, a new band exhibiting a delayed electrophoretic mobility compared to Form II of pDNA was observed (Figure S16) and can be attributed to pDNA fragments of higher molecular weight [98,99]. The overall difference in the photocleavage with UVA radiation was slightly increased in favor of the complexes compared to the ligand.

Exposure to visible light resulted in moderate activity of the compounds towards pDNA (Figure S17). The complexes were moderately active, causing only ss breaks (ss damage up 46%, Figure 8), while 4′-Br-fenH showed appreciable ds damage (of ~22%). On the basis of their UV-vis spectra, the compounds did not seem to absorb at this concentration, and the cleavage seemed to be consistent with the hydrolytic cleavage in the dark, rather than photocleavage.

In conclusion, exposure time and irradiation energy affected the photocleavage activity of the compounds. All the compounds become more active after exposure to UVA or UVB radiation. Free 4′-Br-fenH was more active than its Co(II) complexes when exposed to UVB irradiation and visible light showing its pDNA-cleavage activity. After exposure to UVA irradiation, the complexes were much more active than free 4′-Br-fenH, showed their highest photocleavage activity and induced almost total damage of pDNA. The exposure to visible light resulted in moderate activity of the compounds which, in some cases, was even lower than their activity in the absence of light. Such differentiated (photo)cleavage behavior is noteworthy and may indicate the potential chemotherapeutic efficacy of the compounds. Besides the complexity of the pDNA-photocleavage which was affected by factors such as exposure time, irradiation energy, affinity for DNA and structural diversity, all compounds may be considered photoreactive.

### 2.7. Interaction of the Compounds with Serum Albumins

#### 2.7.1. Affinity of the Compounds for Serum Albumins

Serum albumin (SA) constitutes one of the most abundant proteins of the mammalian bloodstream, with a plethora of biological roles, ranging from regulation of normal blood volume and osmotic pressure to a major drug carrier due to its ability to bind reversibly to a variety of bioactive molecules and drugs [90]. Besides their role as drug carriers, SAs can also be considered as possible targets for anti-cancer treatment [100,101]. The interaction of all herein synthesized compounds with the homologues BSA and HSA was studied with fluorescence emission spectroscopy.

**Figure 9:**
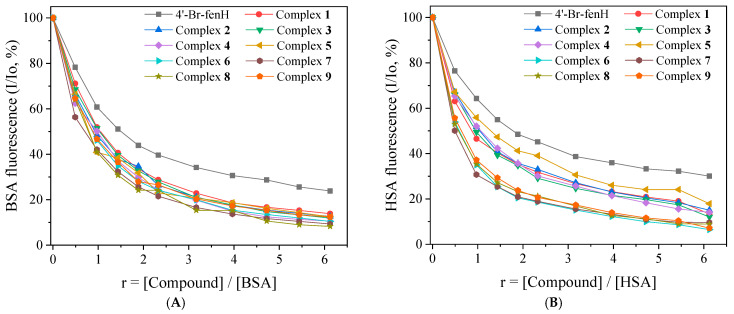
(**A**) Plot of % relative fluorescence intensity of BSA at λ_em_ = 344 nm (I/I_0_, %) versus *r* (*r* = [compound]/[BSA]) for 4′-Br-fenH and its complexes **1**–**9** (up to 23.8% of the initial BSA fluorescence for 4′-Br-fenH, 13.8% for **1**, 12.6% for **2,** 11.9% for **3**, 10.6% for **4**, 12.5% for **5**, 10.4% for **6**, 9.3% for **7**, 8.3% for **8**, and 12.4% for **9**). (**B**) Plot of % relative fluorescence intensity of HSA at λ_em_ = 342 nm (I/I_0_, %) versus *r* (*r* = [compound]/[HSA]) for 4′-Br-fenH and its complexes **1**–**9** (up to 30.0% of the initial HSA fluorescence for 4′-Br-fenH, 13.4% for **1**, 15.0% for **2,** 12.1% for **3**, 14.1% for **4**, 17.9% for **5**, 6.5% for **6**, 9.6% for **7**, 8.9% for **8**, and 7.2% for **9**).

As seen in Figures S18 and S19, when the compounds were added in a solution of serum albumin, the fluorescence intensity (with λ_excitation_ = 295 nm) of the corresponding BSA and HSA band was significantly quenched (up to 93.5% of the initial fluorescence, Figure 9). This quenching indicates structural changes around the tryptophan residues of the serum albumins, and interaction of the compounds with the SAs resulting in changes in the secondary structure of each albumin [90]. The influence of the inner-filter effect on the measurements was determined with Equation (S8) [102] and was negligible.

In order to quantify these results, Stern–Volmer and Scatchard equations (Equations (S2), (S3) and (S9)) and plots (Figures S20–S23) were used to calculate the SA-quenching constants (K_q_) and the SA-binding (K) constants (Table 6) [103]. For K_q_, the value τ_ο_ = 10^−8^ s was used as the fluorescence lifetime of tryptophan in SA. For all compounds, the calculated K_q_ values were approximately three orders higher than the value 10^10^ M^−1^s^−1^, suggesting a static quenching mechanism [90] and confirming the interaction of the compounds with the albumins.

The K values for the compounds were of the 10^5^ M^−1^ order (Table 6) and in the range reported for similar Co(II)-NSAID complexes K_BSA_ = 2.64 × 10^4^–1.08 × 10^7^ M^−1^, K_HSA_ = 1.58 × 10^4^–1.33 × 10^6^ M^−1^) [47,48,49,50,51,52,53,54,73,86,87,88]. The K values for 4′-Br-fenH and its complexes **1**–**9** were lower than the association constants of avidin with diverse compounds (K ≈ 10^15^ M^−1^, this value is considered the highest of known noncovalent interactions), showing that the novel compounds can bind reversibly with SAs, thus being able to be transferred and released at the desired biotargets [104]. It is worth noting that all complexes (especially complexes **7** and **8**) have higher K values than free 4′-Br-fenH for both BSA and HSA, suggesting their enhanced drug delivery potential compared to free 4′-Br-fenH.

#### 2.7.2. Determination of the Albumin-Binding Site

Identifying the albumin site where the synthesized compounds select to bind is a requirement to further understand their interactions with serum albumins. To identify the preferred albumin-site (Sudlow’s site 1 (or drug site I) in subdomain IIA or Sudlow’s site 2 (or drug site II) in subdomain IIIA) for the drugs to bind, competitive experiments were carried out using warfarin and ibuprofen as site-markers. Warfarin and ibuprofen are the most common markers to study these albumin-binding sites, because of their selective binding to drug sites I and II, respectively [105].

When compounds were added to a solution containing SA and one of the markers (warfarin or ibuprofen), a significant decrease in fluorescence was noticed compared to the titration studies performed in the absence of the markers (Figures S24–S27). To pinpoint the preferred binding site, the SA-binding constants for the compounds are calculated in the presence of warfarin or ibuprofen (Table 6), with the Scatchard equation (Equation (S9)) and corresponding plots (Figures S28–S31). A drop in the binding constant (K) in the presence of a marker suggests that the compound’s binding to albumin is influenced competitively by the presence of the respective marker [106].

In the case of BSA, compounds **1**, **2**, **7** and **8** presented significantly lower K values in the presence of marker warfarin, which suggests that they have a binding selectivity for drug site I. On the other hand, compounds **3**, **6** and **9** bore lower K values in the presence of marker ibuprofen, indicating a preference to bind to Sudlow’s site II. Furthermore, 4′-Br-fenH and complexes **4** and **5** had almost equally decreased K values in the presence of both markers, leading to the conclusion that they do not present any binding preference between both sites I and II.

Regarding HSA, the selectivity behavior of the compounds was altered in some cases. More specifically, 4′-Br-fenH and complexes **1**, **2**, **3** and **9** seemed to bind selectively to drug site I while complexes **4**, **6** and **7** seemed to prefer drug site II; for complexes **5** and **8,** any binding selectivity among these sites was not obvious.

## 3. Materials and Methods

### 3.1. Materials—Instrumentation- Physical Measurements

All chemicals and solvents were of analytical reagent grade and were used without further purification. They were purchased from commercial sources: CoCl_2_·6H_2_O, phen, neoc, bipyam, py, αpic, βpic, γpic, Himi, BSA, HSA, EB, ABTS, K_2_CO_3_, CuSO_4_ and KOH from Sigma-Aldrich Co., (Burlington, MA, USA); 2-chlorobenzoic acid, 4-bromoaniline from Alfa Aesar (Heysham, UK); CT DNA, DPPH, BHT, NDGA, K_2_S_2_O_8_ and trolox from J&K Scientific Co., (Beijing, China); sodium warfarin, ibuprofen, and DPPH from Tokyo Chemical industry (TCI); NaCl and trisodium citrate from Merck (Rahway, NJ, USA); H_2_O_2_ (30% *w*/*v*) from PanReac AppliChem ITW Reagents Co., (Barcelona, Spain); supercoiled circular pBR322 plasmid DNA from New England Bioline (Ipswich, MA, USA); L-ascorbic acid, Na_2_HPO_4_, NaH_2_PO_4_, HCl (35% *v*/*v*) and the solvents from Chemlab Co., (Zedelgem, Belgium).

CT DNA was diluted to buffer (15 mM trisodium citrate and 150 mM NaCl at pH 7.0) and was stirred to form a CT DNA stock solution. The resultant stock solution was stored at 4 °C for no longer than ten days. The solution of CT DNA gave a ratio of UV absorbance at 260 and 280 nm (A_260_/A_280_) of 1.87, an indication that the DNA was sufficiently free of protein contamination and its concentration was determined by the measured absorbance at 260 nm after 1:20 dilution (ε = 6600 M^−1^cm^−1^) [107].

Fourier-transform infrared (FT-IR) spectra were obtained using a Thermo Scientific (Waltham, MA, USA) Nicolet iS20 FT-IR ATR spectrometer in the range 400–4000 cm^−1^. The following abbreviations were used to denote peak intensities: vs = very strong; s = strong; m = medium; Δ*v*(COO) = *v*_asym_(COO) − *v*_sym_(COO). The UV-vis spectra were recorded in the range 200–800 nm as nujol mulls and in solution (concentrations in the range of 1 µM–5 mM) on a Jasco V-750 spectrophotometer equipped with an internal thermostat. C, H and N elemental analysis were performed on a Perkin Elmer (Waltham, MA, USA) 240B elemental analyzer. MS spectra were recorded in a Bruker amaZon speed ETD mass spectrometer and a Brucker maXis UHR-TOF with electrospray ionization as the ion source. Molar conductivity measurements of DMSO solutions (1 mM) of the complexes were performed on a Crison Basic 30 conductometer (Crison Instruments, Barcelona, Spain). Fluorescence spectra of the compounds were recorded in solution on a Hitachi F-7000 Fluorescence spectrophotometer (Hitachi, Tokyo, Japan). Viscosity experiments were carried out using an ALPHA L Fungilab rotational viscometer (Fungilab S.A., Barcelona, Spain) equipped with an 18 mL LCP spindle at 100 rpm.

### 3.2. Synthesis and Experimental Data of the Compounds

Analytical synthetic details and the experimental data of the compounds (4′-Br-fenH and its complexes **1**–**9**) are given in the ESI file (Section S1).

The synthesis of 4′-bromo-fenamic acid (4′-Br-fenH) was based on a previously reported procedure [57,58], which was altered to fit our desired product and increase the achieved yield. More specifically, 2-chlorobenzoic acid (1.45 mmol, 227 mg), 4-bromoaniline (2.9 mmol, 500 mg), K_2_CO_3_ (0.725 mmol, 100 mg) and CuSO_4_ (8 mg, catalytic) were dissolved in DMF (5 mL) and refluxed at 153 °C for 24 h. The reaction mixture was allowed to cool to room temperature (RT) and 15 mL of HCl 1 M were added. A precipitate was formed and was collected after standing for 24 h at RT. The filtrate was washed with 200 mL H_2_O and was left to dry under vacuum.

For the synthesis of complex [Co(4′-Br-fen)_2_(MeOH)_4_]·2MeOH (complex **1**), a methanolic solution (13 mL) containing 4′-Br-fenH (0.4 mmol, 100 mg) and KOH (0.4 mmol, 0.4 mL of 1 M solution) was stirred for 1 h, to achieve the deprotonation of the carboxylic group. This solution was afterwards added dropwise into a methanolic solution (7 mL) of CoCl_2_·6H_2_O (0.2 mmol, 48 mg) and the resultant solution was stirred for additional 0.5 h. The mixture was then filtrated, and the filtrate was left to evaporate slowly at RT. After three weeks, orange-yellow crystals were formed, suitable for X-ray structure determination (64 mg, 41%).

Complexes **2**–**4** (i.e., [Co(4′-Br-fen)_2_(*N*,*N*′-donor)(MeOH)_x_], x = 0 or 2) were synthesized in a similar way. A methanolic solution (13 mL) containing 4′-Br-fenH (0.4 mmol, 100 mg) and KOH (0.4 mmol, 0.4 mL of 1 M solution) was stirred for 1-h. Afterwards, the solution was added to a methanolic solution (3 mL) of CoCl_2_·6H_2_O (0.2 mmol, 48 mg) simultaneously with a methanolic solution (3 mL) of the corresponding *N*,*N*′-donor (0.2 mmol) and the reaction solution was stirred for additional 30 min. After filtration, the solution was left to evaporate slowly at RT. The desired product (yield: 42–72%) was collected after two to four weeks.

Complexes **5**–**9** (i.e., [Co(4′-Br-fen)_2_(*N*-donor)_2_(MeOH)_x_], x = 0 or 2) were synthesized in a similar way. More specifically, a methanolic solution (13 mL) containing 4′-Br-fenH (0.4 mmol, 100 mg) and KOH (0.4 mmol, 0.4 mL of 1 M solution) was added, after 1-h stirring, simultaneously with a methanolic solution (3 mL) of the corresponding *N*-donor (0.4 mmol) into a methanolic solution (3 mL) of CoCl_2_·6H_2_O (0.2 mmol, 48 mg). The resultant solution was further stirred for 0.5 h and, after filtration, was left to evaporate slowly at RT. The desired product (yield: ~50%) was collected within a month.

### 3.3. X-Ray Crystal Structure Determination

Suitable single-crystals of compound **1** were mounted on thin glass fibers using epoxy resin. X-ray diffraction data were recorded on a Bruker Apex II CCD area-detector diffractometer, equipped with a Mo Ka (λ = 0.71073 Å) sealed tube source and a Triumph monochromator (Bruker, Billerica, MA, USA) at 295 K, using the φ and ω scans technique. The program Apex2 (Bruker AXS, 2006) was used for data collection and cell refinement. The collected data were integrated with the Bruker SAINT software package [108], using a narrow-frame algorithm. Data were corrected for absorption using the numerical method SADABS [109], based on the crystal dimensions. Structures were solved using the SUPERFLIP package [110] and refined with full-matrix least-squares on *F^2^* using the Crystals program package version 14.61 build 6236 [111]. Anisotropic displacement parameters were applied for all non-hydrogen atoms, while hydrogen atoms were in general found and/or positioned geometrically and refined using a riding model. Details of crystal data and structure refinement parameters are shown in Table S1 [112].

CCDC deposition number 2424334 contains the supplementary crystallographic data for complex **1**. These data can be obtained free of charge via www.ccdc.cam.ac.uk (or from the Cambridge Crystallographic Data Centre, 12 Union Road, Cambridge CB21EZ, UK; fax: (+44) 1223-336-033; or deposit@ccdc.cam.ac.uk).

### 3.4. Study of the Biological Profile of the Compounds

All the procedures and relevant equations used in the in vitro evaluation of the biological activity (antioxidant activity, antimicrobial activity, and interaction with CT DNA, pDNA, HSA and BSA) of the compounds can be found in the Supporting Information file (Sections S2–S6).

## 4. Conclusions

Nine neutral mononuclear Co(II) complexes of a bromo-derivative of 4′-bromo-fenamic acid (4′-Br-fenH) in the presence of nitrogen-donors as co-ligands were isolated and characterized with diverse techniques including ESI-MS/HRMS, and IR and UV-vis spectroscopies. The crystal structure of [Co(4′-Br-fen)_2_(MeOH)_4_]·2MeOH (complex **1**) was determined with single-crystal X-ray crystallography. In all these complexes, the deprotonated 4′-Br-fenamato ligands bound asymmetrically to Co(II) within a distorted octahedral environment.

The in vitro biological properties of the compounds were evaluated based on their interaction with biomacromolecules (albumins and DNA), as well as antioxidant and antimicrobial potency. Antioxidant potency was assessed through their ability to scavenge DPPH and ABTS radicals and to reduce H_2_O_2_. Almost all compounds were inactive against DPPH radicals with complex **9** being the most effective DPPH-scavenger, albeit with only moderate time-dependent activity (DPPH% = 21.98–35.65%). In contrast, most complexes exhibited significant ABTS-scavenging activity (ABTS%~96%) comparable to that of the reference compound trolox. Regarding H_2_O_2_ reduction, all complexes were found to be more active than the reference L-ascorbic acid.

The antimicrobial activity of the compounds was tested against four bacterial strains (*P. aeruginosa*, *E. coli*, *S. epidermidis* and *S. aureus*). According to the MIC values, all the complexes display stronger activity than free 4′-Br-fenH against all bacterial strains tested. The most pronounced activity was found for complexes **3** and **7** against *E. coli* (MIC value of 20 μM). In general, compounds bearing bidentate chelating *N*,*N*′-donor co-ligands (compounds **2**–**4**) showed better activity than those with monodentate *N*-donor co-ligands (compounds **5**–**9**) which is consistent with the chelation theory. Evaluation of their bactericidal potential revealed that complexes **4**, **5**, **8**, and **9** can be classified as bactericidal agents against selected bacterial strains, namely complexes **4** and **5** against *E. coli*, complex **8** against *E. coli* and *S. aureus*, and complex **9** against *S. epidermidis* and *S. aureus*. Based on the IZ values, only complex **3** was found to be susceptible to *S. epidermidis* and intermediate against *E. coli* and *S. aureus* strains.

The complexes are suggested to interact with CT DNA primarily via intercalation (as indicated from the applied techniques, the observed thermal denaturation effects (ΔT_m_) and the calculated thermodynamics parameters (ΔH and ΔS)). They showed relatively strong DNA-binding affinities, with K_b_ values in the range 10^5^–10^6^ M^−1^; the highest DNA-binding constant was observed for complex **6** (K_b_ = 2.00 × 10^6^ M^−1^). The ability of the complexes to cleave pBR322 plasmid DNA into relaxed circular DNA was moderate at a concentration of 500 μM, but was highly enhanced upon irradiation with UVA, UVB or visible light. Complexes **5**–**9** were very active pDNA photocleaving agents under UVA-irradiation (inducing almost complete total plasmid DNA damage).

The interaction of the compounds with serum albumins was tight and reversible, suggesting their potential for transport and controlled release at biological targets. Competitive studies with the typical site-markers warfarin and ibuprofen were employed to assess whether the compounds bind selectively to BSA-binding sites. Considering BSA, complexes **1**, **2**, **7** and **8** seemed to bind selectively to drug site I, whereas complexes **3**, **6** and **9** preferentially bound to Sudlow’s site II. Regarding HSA, some alterations were observed: 4′-Br-fenH and complexes **1**, **2**, **3** and **9** showed selectivity for Sudlow’s site I, while complexes **4**, **6** and **7** bound preferentially to drug site II.

A direct correlation between antibacterial activity and interaction with CT DNA cannot be established, as the determined K_b_ values of the complexes were relatively close. For instance, complex **6** had the highest K_b_ and showed good (though not the best) activity against *E. coli*, but rather only moderate activity against *S. epidermidis* and *S. aureus*. On the other hand, the most effective antibacterial agents, i.e., complexes **2**, **3** and **7**, showed relatively high, though not the highest, K_b_ values.

These findings highlight the therapeutic potential of the synthesized compounds, suggesting novel avenues for their application in oxidative stress management, cancer treatment and drug delivery systems. Future studies should investigate their mechanisms of action and assess their in vivo efficacy.

## Figures and Tables

**Table 1:** Selected bond lengths (Å) and angles (°) for complex **1**.

**Bond**	**Length (Å)**	**Bond**	**Distance (Å)**
Co1—O1	2.0391 (19)	O1—C1	1.260 (3)
Co1—O3	2.0966 (19)	O2—C1	1.264 (3)
Co1—O4	2.1253 (17)		
**Bonds**	**Angle (°)**	**Bonds**	**Angle (°)**
O1—Co1—O4	88.86 (8)	O3—Co1—O4	92.50 (8)
O1—Co1—O3	90.58 (8)	O3 ^i^—Co1—O4	87.50 (8)
O1 ^i^—Co1—O3	89.42 (8)	O1 ^i^—Co1—O4	91.14 (8)

Symmetry code: (i) −*x* + 2, −*y*, −*z* + 2.

**Table 2:** %DPPH-scavenging ability (DPPH%), %ABTS radical scavenging activity (ABTS %) and H_2_O_2_-reducing ability (H_2_O_2_%) for the compounds.

Compound	DPPH%, 30 min/60 min	ABTS%	H_2_O_2_%
4′-Br-fenH	Not active	0.84 ± 0.54	65.60 ± 0.76
Complex **1**	Not active	96.02 ± 0.64	64.74 ± 0.93
Complex **2**	3.93 ± 0.59/8.29 ± 0.71	94.93 ± 0.24	60.43 ± 0.04
Complex **3**	Not active	87.52 ± 0.63	96.93 ± 0.53
Complex **4**	3.37 ± 0.38/10.47 ± 0.44	81.86 ± 0.26	59.20 ± 0.24
Complex **5**	Not active	89.18 ± 0.45	58.85 ± 0.22
Complex **6**	Not active	93.34 ± 0.61	76.94 ± 0.22
Complex **7**	Not active	96.23 ± 0.14	82.73 ± 0.78
Complex **8**	Not active	95.84 ± 0.11	71.70 ± 0.55
Complex **9**	21.98 ± 0.11/35.65 ± 1.05	96.48 ± 0.07	74.74 ± 0.38
NDGA	93.51 ± 1.28/92.68 ± 0.86	Not tested	Not tested
BHT	70.23 ± 9.50/86.54 ± 0.53	Not tested	Not tested
trolox	Not tested	98.10 ± 0.48	Not tested
L-ascorbic acid	Not tested	Not tested	58.90 ± 0.52

**Table 3:** Minimum Inhibitory Concentration (MIC, in μΜ), Bactericidal Concentration (MBC, in μΜ), ratio MBC/MIC, and Inhibition zone (IZ, in mm) of 4′-Br-fenH and its complexes **1**–**9** against four bacterial strains (*P. aeruginosa*, *E. coli*, *S. epidermidis* and *S. aureus*).

Compound	*P. aeruginosa*		*E. coli*		*S. epidermidis*		*S. aureus*	
	MIC (μΜ)	MBC (μΜ)	MIC (μΜ)	MBC (μΜ)	MIC (μΜ)	MBC (μΜ)	MIC (μΜ)	MBC (μΜ)
4′-Br-fenH	>250	-	207.9 ± 8.7	>250	215.3 ± 1.5	>250	250 ± 0.0	>250
Complex **1**	>250	-	79.5 ± 11.5	>200	168.1 ± 18.6	200	146.6 ± 16.1	>200
Complex **2**	>250	-	30.4 ± 7.1	>250	60.5 ± 2.1	>250	56.4 ± 1.0	>250
Complex **3**	>250	-	20.0 ± 0.0	>250	74.6 ± 0.9	>250	50.3 ± 0.8	>250
Complex **4**	>250	-	85.3 ± 3.3	133 ± 33	106.5 ± 2.4	>250	128.7 ± 1.0	250
Complex **5**	>250	-	115.7 ± 5.1	205 ± 30	94.7 ± 7.3	>250	98.0 ± 1.3	>250
Complex **6**	>250	-	44.8 ± 4.9	>250	98.9 ± 11.1	>250	99.1 ± 3.3	>250
Complex **7**	>250	-	20.1 ± 0.2	>250	97.4 ± 2.0	>250	61.1 ± 7.7	250
Complex **8**	>250	-	78.5 ± 6.5	75 ± 19	107.8 ± 0.9	>250	64.8 ± 5.6	115 ± 39
Complex **9**	>250	-	66.3 ± 7.4	>200	197.4 ± 24.6	200	149.8 ± 18.4	150
	**MBC/MIC**	**IZ** **(mm)**	**MBC/MIC**	**IZ** **(mm)**	**MBC/MIC**	**IZ** **(mm)**	**MBC/MIC**	**IZ** **(mm)**
4′-Br-fenH	-	9.0 ± 0.0	-	9.0 ± 0.0	-	9.0 ± 0.0	-	9.0 ± 0.0
Complex **1**	-	9.1 ± 0.2	-	12.8 ± 0.5	1.2	10.8 ± 0.8	-	9.8 ± 0.9
Complex **2**	-	9.0 ± 0.0	-	12.8 ± 0.9	-	11.5 ± 0.9	-	12.0 ± 0.0
Complex **3**	-	9.0 ± 0.0	-	16.1 ± 1.2	-	19.5 ± 0.9	-	16.7 ± 1.3
Complex **4**	-	9.0 ± 0.0	1.6	10.9 ± 0.8	-	9.3 ± 0.5	-	12.0 ± 1.1
Complex **5**	-	9.3 ± 0.5	1.8	12.0 ± 1.1	-	9.8 ± 0.5	-	10.4 ± 0.7
Complex **6**	-	9.0 ± 0.0	-	11.3 ± 0.7	-	9.0 ± 0.0	-	11.5 ± 0.9
Complex **7**	-	9.0 ± 0.0	-	10.8 ± 0.5	-	9.8 ± 1.1	4.1	11.9 ± 0.6
Complex **8**	-	9.0 ± 0.0	1.0	11.9 ± 0.8	-	10.0 ± 0.9	1.8	11.3 ± 0.7
Complex **9**	-	9.2 ± 0.5	-	13.3 ± 0.5	1.0	9.4 ± 0.5	1.0	9.8 ± 0.9

**Table 4:** UV-vis spectroscopic data concerning the interaction of 4′-Br-fenH and its complexes **1**–**9** with CT DNA: UV-band (λ_max_, in nm) (percentage of the observed hyper-/hypo-chromism (ΔA/A_0_, in %), blue-/red-shift of the λ_max_ (Δλ, in nm)); DNA-binding constant (K_b_, in M^−1^); T_m_ (in °C).

Compound	λ_max_ (nm) (ΔA/A_0_ (%)) ^a^, Δλ (nm) ^b^)	K_b_ (M^−1^)	T_m_ (°C)
4′-Br-fenH	301 (+8, ^a^ +9 ^b^); 347 (−40, elim ^c^)	4.42 (±0.30) × 10^4^	82.6
Complex **1**	304 (+5, ^a^ +2 ^b^)	8.90 (±0.19) × 10^5^	84.3
Complex **2**	285 (−5, 0); 311 (−5, −2)	6.73 (±0.20) × 10^5^	84.8
Complex **3**	286 (+13, 0); 312 (+10, +2)	7.92 (±0.35) × 10^5^	85.0
Complex **4**	297 (+5, +6); 347 (−20, elim)	9.92 (±0.14) × 10^5^	87.7
Complex **5**	285 (−10, +20)	3.61 (±0.10) × 10^5^	83.5
Complex **6**	307 (+3, +3); 350 (−30, elim)	2.00 (±0.07) × 10^6^	85.7
Complex **7**	311 (−15, 0)	1.56 (±0.07) × 10^6^	83.8
Complex **8**	306 (−11, −1)	1.21 (±0.19) × 10^6^	84.5
Complex **9**	308 (−2, 0)	9.37 (±0.65) × 10^5^	84.6

^a^ “+” denotes hyperchromism; “−” denotes hypochromism. ^b^ “+” denotes red-shift; “−” denotes blue-shift. ^c^ “elim” = eliminated.

**Table 5:** Fluorescence features of the EB-displacement studies of 4′-Br-fenH and its complexes **1**–**9**: percentage of EB-DNA fluorescence emission quenching (ΔI/I_0_, in %), Stern-Volmer (K_SV_, in M^−1^) and quenching constants (K_q_, in M^−1^s^−1^).

Compound	ΔI/I_0_ (%)	K_SV_ (M^−1^)	K_q_ (M^−1^s^−1^)
4′-Br-fenH	39.1	2.59 (±0.07) × 10^4^	1.13 (±0.03) × 10^12^
Complex **1**	56.8	5.74 (±0.03) × 10^4^	2.49 (±0.11) × 10^12^
Complex **2**	63.5	1.13 (±0.04) × 10^5^	4.90 (±0.17) × 10^12^
Complex **3**	66.6	1.11 (±0.04) × 10^5^	4.84 (±0.16) × 10^12^
Complex **4**	70.5	1.52 (±0.05) × 10^5^	6.60 (±0.23) × 10^12^
Complex **5**	57.8	1.27 (±0.07) × 10^5^	5.53 (±0.30) × 10^12^
Complex **6**	65.5	5.78 (±0.20) × 10^4^	2.51 (±0.09) × 10^12^
Complex **7**	68.3	6.42 (±0.22) × 10^4^	2.79 (±0.09) × 10^12^
Complex **8**	66.2	9.90 (±0.34) × 10^4^	4.31 (±0.15) × 10^12^
Complex **9**	74.2	4.45 (±0.15) × 10^4^	1.94 (±0.07) × 10^12^

**Table 6:** The BSA/HSA-quenching constants (K_q_, in M^−1^s^−1^) calculated for 4′-Br-fenH and its complexes **1**–**9**. The BSA/HSA-binding constants (K, in M^−1^) calculated for 4′-Br-fenH and its complexes **1**–**9** in the absence or presence of the albumin-site marker warfarin and ibuprofen.

**Compound**	**K_q (BSA)_ (M^−1^s^−1^)**	**K_(BSA)_ (M^−1^)**	**K_(BSA, warfarin)_ (M^−1^)**	**K_(BSA, ibuprofen)_ (M^−1^)**
4′-Br-fenH	1.74 (±0.09) × 10^13^	2.72 (±0.09) × 10^5^	1.65 (±0.06) × 10^5^	1.79 (±0.05) × 10^5^
Complex **1**	2.90 (±0.09) × 10^13^	3.24 (±0.11) × 10^5^	2.46 (±0.05) × 10^5^	2.97 (±0.11) × 10^5^
Complex **2**	3.21 (±0.09) × 10^13^	3.45 (±0.12) × 10^5^	2.13 (±0.05) × 10^5^	3.04 (±0.11) × 10^5^
Complex **3**	3.43 (±0.05) × 10^13^	3.00 (±0.08) × 10^5^	3.20 (±0.10) × 10^5^	2.70 (±0.07) × 10^5^
Complex **4**	4.88 (±0.12) × 10^13^	3.84 (±0.08) × 10^5^	1.36 (±0.06) × 10^5^	1.45 (±0.08) × 10^5^
Complex **5**	3.69 (±0.09) × 10^13^	3.38 (±0.10) × 10^5^	1.23 (±0.04) × 10^5^	1.32 (±0.04) × 10^5^
Complex **6**	4.65 (±0.07) × 10^13^	3.95 (±0.14) × 10^5^	2.75 (±0.05) × 10^5^	2.16 (±0.06) × 10^5^
Complex **7**	5.34 (±0.39) × 10^13^	5.00 (±0.15) × 10^5^	1.01 (±0.05) × 10^5^	2.02 (±0.06) × 10^5^
Complex **8**	6.23 (±0.09) × 10^13^	4.69 (±0.13) × 10^5^	1.02 (±0.05) × 10^5^	6.11 (±0.37) × 10^5^
Complex **9**	3.89 (±0.72) × 10^13^	3.72 (±0.84) × 10^5^	3.47 (±0.11) × 10^5^	3.10 (±0.10) × 10^5^
**Compound**	**K_q (HSA)_ (M^−1^s^−1^)**	**K_(HSA)_ (M^−1^)**	**K_(HSA, warfarin)_ (M^−1^)**	**K_(HSA, ibuprofen)_ (M^−1^)**
4′-Br-fenH	1.52 (±0.75) × 10^13^	2.70 (±0.06) × 10^5^	1.96 (±0.06) × 10^5^	2.26 (±0.07) × 10^5^
Complex **1**	2.14 (±0.01) × 10^13^	4.77 (±0.15) × 10^5^	2.32 (±0.88) × 10^5^	2.94 (±0.15) × 10^5^
Complex **2**	2.36 (±0.08) × 10^13^	3.60 (±0.10) × 10^5^	1.14 (±0.04) × 10^5^	3.10 (±0.08) × 10^5^
Complex **3**	2.43 (±0.11) × 10^13^	3.83 (±0.09) × 10^5^	2.22 (±0.10) × 10^5^	2.85 (±0.10) × 10^5^
Complex **4**	2.43 (±0.14) × 10^13^	3.80 (±0.10) × 10^5^	4.62 (±0.15) × 10^5^	3.46 (±0.12) × 10^5^
Complex **5**	2.36 (±0.08) × 10^13^	2.90 (±0.11) × 10^5^	4.57 (±0.14) × 10^5^	3.74 (±0.16) × 10^5^
Complex **6**	6.32 (±0.16) × 10^13^	5.82 (±0.23) × 10^5^	4.92 (±0.25) × 10^5^	9.53 (±0.21) × 10^5^
Complex **7**	5.45 (±0.16) × 10^13^	7.15 (±0.15) × 10^5^	5.34 (±0.20) × 10^5^	3.52 (±0.21) × 10^5^
Complex **8**	5.82 (±0.21) × 10^13^	6.20 (±0.18) × 10^5^	7.19 (±0.23) × 10^5^	10.20 (±0.30) × 10^5^
Complex **9**	5.21 (±0.09) × 10^13^	5.58 (±0.15) × 10^5^	4.57 (±0.14) × 10^5^	6.51 (±0.23) × 10^5^

## Data Availability

All necessary supplementary data are included in the Supplementary Materials.

## Supplementary Materials

The supporting information can be downloaded at: https://www.mdpi.com/article/10.3390/ijms26199787/s1.

## Abbreviations

The following abbreviations are used in this manuscript:

4′-Br-fen^−1^	4′-bromofenamato anion
4′-Br-fenH	4′-bromofenamic acid
ABTS	2,2′-azinobis-(3-ethylbenzothiazoline-6-sulfonic acid)
BHT	butylated hydroxytoluene
bipyam	2,2′-bipyridylamine
BSA	bovine serum albumin
COX	cyclooxygenase
CT	calf-thymus
dicl^−1^	anion of diclofenac
DPPH	1,1-diphenyl-picrylhydrazyl
*E. coli*	*Escherichia coli*
EB	ethidium bromide
fen^−1^	fenamato anion
fluf^−1^	flufenamato anion
Hdicl	diclofenac
Hfen	fenamic acid
Hfluf	flufenamic acid
Himi	*1H*-imidazole
Hmeclf	meclofenamic acid
Hmef	mefenamic acid
Hnap	naproxen
Hnif	niflumic acid
HSA	human serum albumin
Htolf	tolfenamic acid
IZ	inhibition zone
K	SA-binding constant
K_b_	DNA-binding constant
K_q_	quenching constant
K_SV_	Stern-Volmer constant
MBC	minimum bactericidal concentration
meclf^−1^	meclofenamato anion
mef^−1^	mefenamato anion
MIC	minimum inhibitory concentration
nap^−1^	anion of naproxen
NDGA	nordihydroguaiaretic acid
neoc	neocuproine (2,9-dimethyl-1,10-phenanthroline)
nif^−1^	niflumato anion
NSAID	non-steroidal anti-inflammatory drug
*P. aeruginosa*	*Pseudomonas aeruginosa*
pDNA	pBR322 plasmid DNA
phen	1,10-phenanthroline
py	pyridine
ROS	reactive oxygen species
RT	room temperature
*S. aureus*	*Staphylococcus aureus*
*S. epidermidis*	*Staphylococcus epidermidis*
SA	serum albumin
T_m_	DNA-melting temperature
tolf^−1^	tolfenamato anion
trolox	6-hydroxy-2,5,7,8-tetramethylchroman-2-carboxylic acid
αpic	α-picoline (2-methylpyridine)
βpic	β-picoline (3-methylpyridine)
γpic	γ-picoline (4-methylpyridine)
