# Supplementary material for: Cobalt(II) Complexes of 4′-Bromo-Fenamic Acid: Antioxidant Properties, Antibacterial Activity, and Interaction with DNA and Albumins
Source: Int J Mol Sci. 2025 Oct 8;26(19):9787. doi: 10.3390/ijms26199787 (PMC12524402; doi:10.3390/ijms26199787)
Supplement: Supplementary file 1 [file ijms-26-09787-s001.zip › Malis - Psomas, ESI for IJMS.pdf]

Cobalt(II) complexes of 4′-bromo-fenamic acid: Antioxidant properties, antibacterial activity, and interaction with DNA and albumins

Georgios Malis[1], Christina N. Banti[2], Alexia Tialiou[3,4], Michael R. Reithofer[3], Antonios G. Hatzidimitriou[1], Sotiris K. Hadjikakou[2,5,*], Konstantina C. Fylaktakidou[6], and George Psomas[1,*]

[1] *Laboratory of Inorganic Chemistry, Department of Chemistry, Aristotle University of Thessaloniki, GR-54124 Thessaloniki, Greece*
[2] *Department of Chemistry, University of Ioannina, GR-45110 Ioannina, Greece*
[3] *Institute of Inorganic Chemistry, Faculty of Chemistry, University of Vienna, Währinger Str. 42, 1090 Vienna, Austria.*
[4] *Vienna Doctoral School in Chemistry (DoSChem), University of Vienna, Währinger Str. 42, 1090 Vienna, Austria*
[5] *University Research Centre of Ioannina (URCI), Institute of Materials Science and Computing, Ioannina, Greece*
[6] *Laboratory of Organic Chemistry, Department of Chemistry, Aristotle University of Thessaloniki, GR-54124 Thessaloniki, Greece*

SUPPLEMENTARY MATERIAL

* Corresponding authors' e-mails: shadjika@uoi.gr (S.K. Hadjikakou); gepsomas@chem.auth.gr (G. Psomas)

Content

S1 SYNTHESIS and EXPERIMENTAL DATA of the COMPOUNDS 5
4′-bromo-fenamic acid (4′-Br-fenH) 5
[Co(4′-Br-fen)$_2$(MeOH)$_4$]·2MeOH (complex **1**) 5
[Co(4′-Br-fen)$_2$(phen)(MeOH)$_2$] (complex **2**) 5
[Co(4′-Br-fen)$_2$(neoc)(MeOH)$_2$] (complex **3**) 5
[Co(4′-Br-fen)$_2$(bipyam)] (complex **4**) 6
[Co(4′-Br-fen)$_2$(Himi)$_2$] (complex **5**) 6
[Co(4′-Br-fen)$_2$(py)$_2$(MeOH)$_2$] (complex **6**) 6
[Co(4′-Br-fen)$_2$(αpic)$_2$(MeOH)$_2$] (complex **7**) 6
[Co(4′-Br-fen)$_2$(βpic)$_2$(MeOH)$_2$] (complex **8**) 7
[Co(4′-Br-fen)$_2$(γpic)$_2$(MeOH)$_2$] (complex **9**) 7
EXPERIMENTAL PROTOCOLS 7
S2 Antioxidant activity assay 7
S2.1 Determination of the reducing activity of the radical DPPH 8
S2.2 Assay of radical cation ABTS-scavenging activity 8
S2.3 Reduction of hydrogen peroxide 8
S3 Antibacterial Activity studies 9
S3.1 Bacterial Strains 9
S3.2 Antibacterial effects of compounds on the growth of microbial strains 9
S4 Binding studies with CT DNA 9
S4.1 Binding study with CT DNA by UV-vis spectroscopy 10
S4.2 CT DNA-binding studies by viscosity measurements 10
S4.3 EB-displacement studies 10
S4.4 Determination of the DNA-melting temperature (T_m) 11
S4.5 Determination of the thermodynamic parameters 11
S5 Plasmid DNA cleavage experiments 11
S6 Albumin-binding studies 12
S6.1 Interaction with albumins 12
S6.2 Competitive albumin-fluorescence studies with warfarin and ibuprofen 13
S7 References 13
TABLES 15
Table S1. Experimental crystallographic details for complex **1**. 15

Table S2. Hydrogen bonds (lengths in Å, angles in °) for complex **1**. 16

Table S3. Thermodynamic parameters of the compounds for the interaction with CT DNA at different temperatures (295 K, 303 K and 310 K). 17

FIGURES 18

Figure S1. ^{1}H NMR spectrum of 4′-Br-fenH in DMSO-d_6. 18

Figure S2. IR spectra (ATR) of the compounds. 19

Figure S3. Mass spectra recorded for complexes **1-9**. 22

Figure S4. Visible spectra recorded for complexes **4** and **9** in the absence and presence of buffer solution for two different time-intervals. 25

Figure S5: Proposed structures for complexes **2-9.** 26

Figure S6. Minimum bactericidal concentration 4′-Br-fenH and its complexes **1-9** against *E. coli, S. epidermidis* and *S. aureus*. 27

Figure S7. IZs which are developed in agar plates of *P. aeruginosa, E. coli, S. epidermidis* and *S. aureus* by 4′-Br-fenH and its complexes **1-9** and at 1 mM. 30

Figure S8. UV-vis spectra of a DMSO solution of the compounds (4′-Br-fenH and its complexes **1-9**) in the presence of increasing amounts of CT DNA. 31

Figure S9. Plots of $\frac{[DNA]}{(\varepsilon_A - \varepsilon_f)}$ *versus* [DNA] for the compounds. 33

Figure S10. Fluorescence emission spectra for EB-DNA in buffer solution in the absence and presence of increasing amounts of the compounds. 35

Figure S11. Stern-Volmer plots of the EB-DNA quenching experiments upon addition of the compounds. 37

Figure S12. Thermal melting profile of CT DNA in the absence or presence of the compounds. 39

Figure S13. van't Hoff plots for the interaction of CT DNA with the compounds. 41

Figure S14: Agarose gel electrophoretic pattern of EB-stained plasmid DNA (pBR322 plasmid DNA) after incubation with the compounds, in dark. 43

Figure S15: Agarose gel electrophoretic pattern of EB-stained plasmid DNA (pBR322 plasmid DNA) with the compounds, after irradiation at 312 nm (UV-B) for 30 min. 44

Figure S16: Agarose gel electrophoretic pattern of EB-stained plasmid DNA (pBR322 plasmid DNA) with the compounds, after irradiation at 365 nm (UV-A) for 30 min. 45

Figure S17: Agarose gel electrophoretic pattern of EB-stained plasmid DNA (pBR322 plasmid DNA) with the compounds, after irradiation under visible light for 2 h. 46

Figure S18. Fluorescence emission spectra of BSA in buffer solution in the presence of increasing amounts of the compounds. 47

Figure S19. Fluorescence emission spectra of HSA in buffer solution in the presence of increasing amounts of the compounds. 49

Figure S20. Stern-Volmer plots of the BSA-quenching experiments upon addition of the compounds....51

Figure S21. Stern-Volmer plots of the HSA-quenching experiments upon addition of the compounds....53

Figure S22. Scatchard plots of the BSA-quenching experiments upon addition of the compounds....55

Figure S23. Scatchard plots of the HSA-quenching experiments upon addition of the compounds....57

Figure S24. Fluorescence emission spectra of BSA in buffer solution in the presence of warfarin upon addition of increasing amounts of the compounds....59

Figure S25. Fluorescence emission spectra of BSA in buffer solution in the presence of ibuprofen upon addition of increasing amounts of the compounds....61

Figure S26. Fluorescence emission spectra of HSA in buffer solution in the presence of warfarin upon addition of increasing amounts of the compounds....63

Figure S27. Fluorescence emission spectra of HSA in buffer solution in the presence of ibuprofen upon addition of increasing amounts of the compounds....65

Figure S28. Scatchard plots of the BSA quenching experiments in the presence of warfarin upon addition of the compounds....67

Figure S29. Scatchard plots of the BSA quenching experiments in the presence of ibuprofen upon addition of the compounds....69

Figure S30. Scatchard plots of the HSA quenching experiments in the presence of warfarin upon addition of the compounds....71

Figure S31. Scatchard plots of the HSA quenching experiments in the presence of ibuprofen upon addition of the compounds....73

S1 SYNTHESIS and EXPERIMENTAL DATA of the COMPOUNDS

4′-bromo-fenamic acid (4′-Br-fenH)

White-yellow solid, m.p. 179-181°C (lit.: 177-180 °C; m.p., IR and ^{1}H-NMR spectra are in correlation with reference [1]) recrystallized in EtOH, (77 %) was collected. IR (ATR), υ/cm^{-1}: ν(N-H): 3313 (m), 3148 (m); ν(C=O): 1667 (vs); ν(C=C): 1608 (vs); ν(C-Br): 664 (m). ^{1}H NMR (500 MHz, DMSO-d_6), δ (ppm) (Figure S1): 13.12 (s, 1H), 9.60 (s, 1H), 7.90 (brs, 1H), 7.49 (d, J = 8.3 Hz, 2H), 7.40 (t, J = 7.8 Hz, 1H), 7.26 (brs, 1H), 7.21 (d, J = 8.4 Hz, 2H), 6.83 (brt, J = 7.0 Hz, 1H). The compound is soluble in MeOH, MeCN, DMF and DMSO.

[Co(4′-Br-fen)$_2$(MeOH)$_4$]·2MeOH **(**complex **1)**

Anal. Calc for [Co(4′-Br-fen)$_2$(MeOH)$_4$]·2MeOH ($C_{32}H_{42}Br_2CoN_2O_{10}$) (MW = 833.43): C, 46.12; H, 5.08; N, 3.36%. Found: C, 45.95; H, 5.01; N, 3.48%. ESI(+) MS in MeOH/MeCN, found (calculated) (m/z): 737.84 (738.33 for [Co(4′-Br-fen)$_2$(MeOH)$_3$]$^+$, 770.36 for [Co(4′-Br-fen)$_2$(MeOH)$_4$]$^+$). IR (ATR), υ/cm^{-1}: υ_{asym}(COO): 1579 (vs); υ_{sym}(COO): 1386 (s); $\Delta\upsilon$(COO) = 193. UV-vis in DMSO solution, λ_{max}/nm (ε/M^{-1}cm^{-1}): 521 (50), 396 (350), 306 (21000). The complex is soluble in MeOH, EtOH, MeCN, DMF and DMSO (Λ_M = 12 S·cm^2·mol^{-1} in 1 mM DMSO), and partially soluble in H_2O.

[Co(4′-Br-fen)$_2$(phen)(MeOH)$_2$] (complex **2**)

Phen (0.2 mmol, 36 mg) was used as the corresponding *N,N′*-donor. Orange-yellow microcrystalline product (75 mg, 42%) was collected after two weeks. Anal. Calc for [Co(4′-Br-fen)$_2$(phen)(MeOH)$_2$] ($C_{40}H_{34}Br_2CoN_4O_6$) (MW = 885.49): C, 54.26; H, 3.87; N, 6.33%. Found: C, 54.68; H, 4.03; N, 6.48%. HRMS(+) in MeOH/MeCN, found (calculated) (m/z): 882.9963 (882.9999 for [Co(4′-Br-fen)$_2$(phen)(MeOH)$_2$]$^+$). IR (ATR), υ/cm^{-1}: υ_{asym}(COO): 1581 (vs); υ_{sym}(COO): 1384 (s); $\Delta\upsilon$(COO) = 197; ρ(C-H)$_{phen}$ = 720 (m). UV-vis in DMSO solution, λ_{max}/nm (ε/M^{-1}cm^{-1}): 482 (235), 302 (21400), 273 (18700). The complex is soluble in MeOH, EtOH, MeCN, DMF, and DMSO (Λ_M = 10 S·cm^2·mol^{-1} in 1 mM DMSO), and partially soluble in H_2O.

[Co(4′-Br-fen)$_2$(neoc)(MeOH)$_2$] (complex **3**)

Neoc (0.2 mmol, 42 mg) was used as the corresponding *N,N′*-donor. Brown-orange microcrystalline product (107 mg, 59%) was collected after several weeks. Anal. Calc for [Co(4′-Br-fen)$_2$(neoc)(MeOH)$_2$] ($C_{42}H_{38}Br_2CoN_4O_6$) (MW = 913.54): C, 55.22; H, 4.19; N, 6.13%. Found: C, 55.46; H, 4.05; N, 6.28%. HRMS(+) in MeOH/MeCN, found (calculated) (m/z): 911.7857 (911.0313 for [Co(4′-Br-fen)$_2$(neoc)(MeOH)$_2$]$^+$). IR (ATR), υ/cm^{-1}: υ_{asym}(COO): 1583 (vs); υ_{sym}(COO): 1400 (s); $\Delta\upsilon$(COO) = 183; ρ(C-H)$_{neoc}$ = 730 (m). UV-vis in DMSO solution, λ_{max}/nm (ε/M^{-1}cm^{-1}): 615 (45), 470 (120), 300 (23700), 274 (21200). The complex is soluble in

MeOH, EtOH, MeCN, DMF, and DMSO (Λ_M = 11 S·cm^2·mol^{-1} in 1 mM DMSO), and partially soluble in H_2O.

[Co(4′-Br-fen)$_2$(bipyam)] (complex **4**)

Bipyam (0.2 mmol, 99 mg) was used as the corresponding *N,N′*-donor. orange-yellow microcrystalline product (125 mg, 72%) was collected after twenty days. Anal. Calc for [Co(4′-Br-fen)$_2$(bipyam)] ($C_{36}H_{27}Br_2CoN_5O_4$) (MW = 812.39): C, 53.22; H, 3.35; N, 8.62%. Found: C, 53.36; H, 3.27; N, 8.48%. ESI(-) MS in MeOH/MeCN, found (calculated) (m/z): 810.88 [812.39 for [Co(4′-Br-fen)$_2$(bipyam)(MeOH)$_2$]). IR (ATR), v/cm^{-1}: v_{asym}(COO): 1581 (vs); v_{sym}(COO): 1386 (s); Δv(COO) = 195; ρ(C-H)$_{bipyam}$ = 769 (m). UV-vis in DMSO solution, λ_{max}/nm (ε/M^{-1}cm^{-1}): 592 (220), 484 (300), 311 (34500). The complex is soluble in MeOH, MeCN, DMF, and DMSO (Λ_M = 12 S·cm^2·mol^{-1} in 1 mM DMSO), and partially soluble in H_2O.

[Co(4′-Br-fen)$_2$(Himi)$_2$] (complex **5**)

Himi (0.4 mmol, 27 mg) was used as the corresponding *N*-donor. Yellow-red microcrystalline product was formed and collected after several days (71 mg, 42%). Anal. Calc for [Co(4′-Br-fen)$_2$(Himi)$_2$] ($C_{34}H_{34}Br_2CoN_6O_6$) (MW = 777.35): C, 49.44; H, 3.37; N, 10.81%. Found: C, 49.64; H, 3.30; N, 10.58%. ESI(-) MS in MeOH/MeCN, found (calculated) (m/z): 806.66 (809.39 for **5** + MeOH, i.e. [Co(4′-Br-fen)$_2$(Himi)$_2$] + MeOH). IR (ATR), v_{max}/cm^{-1}: v_{asym}(COO): 1581 (vs); v_{sym}(COO): 1385 (s); Δv(COO) = 196; ρ(C-H)$_{Himi}$ = 750 (m). UV-vis in DMSO solution, λ_{max}/nm (ε/M^{-1}cm^{-1}): 560 (450), 364 (8800), 350 (12300), 282 (45000). The complex is soluble in MeOH, EtOH and DMSO (Λ_M = 10 S·cm^2·mol^{-1} in 1 mM DMSO), and is partially soluble in H_2O.

[Co(4′-Br-fen)$_2$(py)$_2$(MeOH)$_2$] (complex **6)**

Pyridine (0.4 mmol, 40 μL) was used as the corresponding *N*-donor. Brown-orange microcrystalline product was collected after two weeks (85 mg, 49%). Anal. Calc for [Co(4′-Br-fen)$_2$(py)$_2$(MeOH)$_2$] ($C_{38}H_{36}Br_2CoN_4O_6$) (MW = 863.48): C, 52.86; H, 4.20; N, 6.49%. Found: C, 52.78; H, 4.11; N, 6.37%. ESI(+) MS in MeOH/MeCN, found (calculated) (m/z): 829.74 (831.43 for **6** - MeOH, i.e. [Co(4′-Br-fen)$_2$(py)$_2$(MeOH)]). IR (ATR), v/cm^{-1}: v_{asym}(COO): 1580 (vs); v_{sym}(COO): 1386 (s); Δv(COO) = 194; ρ(C-H)$_{py}$ = 698 (m). UV-vis in DMSO solution, λ_{max}/nm (ε/M^{-1}cm^{-1}): 592 (230), 324 (14700). The complex is soluble in MeOH, EtOH, MeCN and DMSO (Λ_M = 13 S·cm^2·mol^{-1} in 1 mM DMSO), and partially soluble in H_2O.

[Co(4′-Br-fen)$_2$(αpic)$_2$(MeOH)$_2$] (complex **7**)

αpic (0.4 mmol, 38 μL) was used as the corresponding *N*-donor. Brown-orange microcrystalline product (89 mg, 50%) was formed after three weeks. Anal. Calc for [Co(4′-Br-fen)$_2$(αpic)$_2$(MeOH)$_2$] ($C_{40}H_{40}Br_2CoN_4O_6$) (MW = 891.53): C, 53.89; H, 4.52; N, 6.28%. Found: C, 53.75; H, 4.57; N, 6.38%. ESI(+) MS in MeOH/MeCN, found (calculated) (m/z):

889.47 (891.53 for [Co(4′-Br-fen)$_2$(αpic)$_2$(MeOH)$_2$]). IR (ATR), v/cm^{-1}: v_{asym}(COO): 1583 (vs); v_{sym}(COO): 1392 (s); Δv(COO) = 191; ρ(C-H)$_{\alpha pic}$ = 702 (m). UV-vis in DMSO solution, λ_{max}/nm (ε/M^{-1}cm^{-1}): 540 (230), 325 (14100). The complex is soluble in MeOH, EtOH, MeCN, DMF and DMSO (Λ_M = 11 S·cm^2·mol^{-1} in 1 mM DMSO), and partially soluble in H_2O.

[Co(4′-Br-fen)$_2$(βpic)$_2$(MeOH)$_2$] (complex **8**)

βpic (0.4 mmol, 38 μL) was used as the corresponding *N*-donor. Brown-orange microcrystalline product (50 mg, 28%) was formed after one month. Anal. Calc for [Co(4′-Br-fen)$_2$(βpic)$_2$(MeOH)$_2$] ($C_{40}H_{40}Br_2CoN_4O_6$) (MW = 891.53): C, 53.89; H, 4.52; N, 6.28%. Found: C, 53.96; H, 4.41; N, 6.20%. ESI(+) MS in MeOH/MeCN, found (calculated) (m/z): 885.94 (890.05 for [Co(4′-Br-fen)$_2$(βpic)$_2$(MeOH)$_2$]); ESI(-) MS in MeOH/MeCN, found (calculated) (m/z): 886.88 (890.05 for [Co(4′-Br-fen)$_2$(βpic)$_2$(MeOH)$_2$]). IR (ATR), v/cm^{-1}: v_{asym}(COO): 1581 (vs); v_{sym}(COO): 1386 (s); Δv(COO) = 195; ρ(C-H)$_{\beta pic}$ = 706 (m). UV-vis in DMSO solution, λ_{max}/nm (ε/M^{-1}cm^{-1}): 656 (75), 538 (150), 313 (22600), 283 (14000). The complex is soluble in MeOH, MeCN, DMF and DMSO (Λ_M = 14 S·cm^2·mol^{-1} in 1 mM DMSO), and partially soluble in H_2O.

[Co(4′-Br-fen)$_2$(γpic)$_2$(MeOH)$_2$] (complex **9**)

γpic (0.4 mmol, 38 μL) was used as the corresponding *N*-donor. Brown-orange microcrystalline product (80 mg, 45%) was formed after two weeks. Anal. Calc for [Co(4′-Br-fen)$_2$(γpic)$_2$(MeOH)$_2$] ($C_{40}H_{40}Br_2CoN_4O_6$) (MW = 891.53): C, 53.89; H, 4.52; N, 6.28%. Found: C, 54.05; H, 4.66; N, 6.43%. ESI(-) MS in MeOH/MeCN, found (calculated) (m/z): 886.83 (890.05 for [Co(4′-Br-fen)$_2$(γpic)$_2$(MeOH)$_2$]). IR (ATR), v/cm^{-1}: v_{asym}(COO): 1581 (vs); v_{sym}(COO): 1385 (s); Δv(COO) = 196; ρ(C-H)$_{\gamma pic}$ = 722 (m). UV-vis in DMSO solution, λ_{max}/nm (ε/M^{-1}cm^{-1}): 538 (280), 290 (10160), 268 (5030). The complex is soluble in MeOH, MeCN, DMF, and DMSO (Λ_M = 15 S·cm^2·mol^{-1} in 1 mM DMSO), and partially soluble in H_2O.

EXPERIMENTAL PROTOCOLS

S2 Antioxidant activity assay

The antioxidant activity of the compound was evaluated *via* the ability to scavenge *in vitro* free radicals such as DPPH and ABTS and to reduce H_2O_2. All the experiments were carried out at least in triplicate and the standard deviation of absorbance was less than 10% of the mean.

S2.1 Determination of the reducing activity of the radical DPPH

To an ethanolic solution of DPPH (0.1 mM) an equal volume solution of the compounds (0.1 mM) in ethanol was added. Absolute ethanol was also used as control solution. The absorbance at 517 nm was recorded at room temperature after 30 and 60 min in order to examine the possible existence of a potential time-dependence of the DPPH radical scavenging activity [2]. The DPPH-scavenging activity of the compounds was expressed as the percentage reduction of the absorbance values of the initial DPPH solution (DPPH%). NDGA and BHT were used as reference compounds.

S2.2 Assay of radical cation ABTS-scavenging activity

The ABTS assay was performed to determine the activity of the compounds to scavenge the radical cation ABTS. Initially, a water solution of ABTS was prepared (2 mM). ABTS radical cation ($ABTS^{+\cdot}$) was produced by the reaction of ABTS stock solution with potassium persulfate (0.17 mM) and the mixture was stored in the dark at room temperature for 12-16 h before its use. The ABTS was oxidized incompletely because the stoichiometric reaction ratio of ABTS and potassium persulfate is 1:0.5. The absorbance became maximal and stable only after more than 6 h of reaction although the oxidation of the ABTS started immediately. The radical was stable in this form for more than 2 days when allowed to stand in the dark at room temperature. Afterwards, the $ABTS^{+\cdot}$ solution was diluted in ethanol to an absorbance of 0.70 at 734 nm and 10 μL of diluted compounds or standards (0.1 mM) in DMSO were added. The absorbance was recorded out exactly 1 min after initial mixing [2]. The ABTS-radical scavenging activity of the compounds was expressed as the percentage inhibition of the absorbance of the initial ABTS solution (ABTS%). Trolox was used as an appropriate standard.

S2.3 Reduction of hydrogen peroxide

The ability of the compounds to reduce hydrogen peroxide (H_2O_2) was estimated according to the method described in the literature [3]. The reaction mixture contained 20 μL of each of the tested compounds (0.1 mM) and 5 μL H_2O_2 solution (40 mM) in phosphate buffer (50 mM, pH 7.4). The absorbance was measured at 230 nm after 10 min. The antioxidant activity (reduction of H_2O_2) of the compounds was expressed as the percentage decrease of the initial H_2O_2 solution (H_2O_2%). L-ascorbic acid (or vitamin C) was used as a standard.

S3 Antibacterial Activity studies

S3.1 Bacterial Strains

For the antibacterial experiments the strain of *Staphylococcus epidermidis* (ATCC® 14990™), *Staphylococcus aureus* subsp. *aureus* (ATCC® 25923™), *P. aeruginosa and Escherichia coli Dh5a* (*E. coli*) were used.

S3.2 Antibacterial effects of compounds on the growth of microbial strains

This study was performed according to standard procedure which is also described elsewhere [4-6]. Briefly, the bacterial strains were streaked onto in trypticase soy agar. The plates were incubated for 18-24 h at 37 ^{o}C. Three to five isolated colonies are selected of the same morphological appearance from the fresh agar plate using a sterile loop and transfer into a tube containing 2 mL of sterile saline solution. The optical density at 620 nm is adjusted to 0.1 which corresponds to 10^8 cfu/mL. For the evaluation of MIC the inoculum size for broth dilution is 5×10^5 cfu/mL. The total volume of the culture solution treated by compounds, as well as the total volume of the positive and negative control was 2 mL. The range of concentrations of compounds is 0.5-250 μM. The growth is assessed after incubation for 20 h.

For the evaluation of MBC, the bacteria were initially cultivated in the presence of compounds, in broth culture for 20 h. The MBC values were determined in duplicate, by subculturing 4 μL of the broth an agar plate [4-6].

The study of IZ agar plates were inoculated with a standardized inoculum (10^8 cfu/mL) of the tested microorganism. Filter paper disks (9 mm in diameter), which have been previously soaked by compounds (1 mM), were placed on the agar surface. The Petri dishes were incubated for 20 h.

S4 Binding studies with CT DNA

In order to study the interaction of the compounds with DNA, the compound was initially dissolved in DMSO (1 mM). Mixing of such solutions with the aqueous buffer solutions of DNA used in the studies never exceeded 5% DMSO (v/v) in the final solution, which was needed due to low aqueous solubility of most compounds. In all experiments, the effect of DMSO on the data has been taken into consideration and the appropriate corrections have been performed. The interaction of the compound with CT DNA was monitored by UV-vis spectroscopy, and viscosity measurements, and *via* competitive studies with EB by fluorescence emission spectroscopy.

S4.1 Binding study with CT DNA by UV-vis spectroscopy

The interaction of the compound with CT DNA has been studied by UV-vis spectroscopy in order to investigate the possible binding mode to CT DNA and to calculate the DNA-binding constant (K_b). The K_b constant (in M^{-1}) of the compounds was determined with the Wolfe-Shimer equation (equation S1) [7] and the plots [DNA]/(ε_A-ε_f) *versus* [DNA] using the UV-vis spectra of the compound (25-100 μM) recorded for a constant concentration with increasing amounts of CT DNA for diverse [compound]/[DNA] mixing ratios (= *r*). According to the Wolfe-Shimer equation:

$$\frac{[\mathrm{DNA}]}{(\varepsilon_A-\varepsilon_f)} = \frac{[\mathrm{DNA}]}{(\varepsilon_b-\varepsilon_f)} + \frac{1}{K_b(\varepsilon_b-\varepsilon_f)} \qquad \text{(equation S1)}$$

where [DNA] is the concentration of DNA in base pairs, $\varepsilon_A = A_{obsd}$/[compound], ε_f = the extinction coefficient for the free compound and ε_b = the extinction coefficient for the compound in the fully bound form. K_b is given by the ratio of slope to the y intercept in plots [DNA]/(ε_A-ε_f) *versus* [DNA].

S4.2 CT DNA-binding studies by viscosity measurements

The interaction of compounds with DNA was evaluated *via* the study of the CT DNA viscosity ([DNA] = 0.1 mM) in a buffer solution (150 mM NaCl and 15 mM trisodium citrate at pH 7.0) in the presence of increasing amounts of the compounds (up to the value of *r* = 0.32). The obtained data are presented as $(\eta/\eta_0)^{1/3}$ *versus r*, where η is the viscosity of DNA in the presence of the compound, and η_0 is the viscosity of DNA alone in buffer solution.

S4.3 EB-displacement studies

The competition of the compounds with EB was investigated by fluorescence emission spectroscopy to examine whether the compounds can displace EB from its DNA-EB adduct. The CT DNA-EB complex was formed by pre-treating 40 μM EB and 46 μM CT DNA in buffer (150 mM NaCl and 15 mM trisodium citrate at pH 7.0). The possible displacement of EB by the compound sand subsequently the intercalating effect was studied by the stepwise addition of a certain amount of the solution of each compound into the solution of the CT DNA-EB adduct. The solutions were excited at 540 nm and the emission was monitored from 550-700 nm with λ_{max} = 592-594 nm and the effect of the addition of the compounds to the CT-DNA EB solution was recorded. The compounds did not display any fluorescence emission bands at room temperature in solution or in the presence of CT DNA or EB under the same experimental conditions ($\lambda_{excitation}$ = 540 nm); therefore, the observed quenching of the EB-DNA solution may be attributed to the displacement of EB from its EB-DNA adduct.

The Stern-Volmer constants (K_{SV}, in M^{-1}) were calculated according to the linear Stern-Volmer equation (equation S2) [8] and the respective plots Io/I *versus* [compound]:

$$\frac{Io}{I} = 1 + K_q\tau_0[Q] = 1 + K_{SV}[Q] \qquad \text{(equation S2)}$$

where Io and I are the emission intensities of the EB-DNA solution in the absence and the presence of the compound, respectively, τ_o = the average lifetime of the emitting system without the quencher and k_q = the quenching constant. Taking τ_o = 23 ns as the fluorescence lifetime of the EB-DNA adduct [9], the quenching constant of the compound (K_q, in $M^{-1}s^{-1}$) was calculated according to equation [8]:

$$K_{SV} = K_q \times \tau_o \qquad \text{(equation S3)}$$

S4.4 Determination of the DNA-melting temperature (T_m)

The UV-vis spectra of a CT DNA solution (1.25×10^{-4} M) in the presence of the compounds (2.5×10^{-6} M) were recorded upon increasing the temperature. The obtained data are presented as normalized absorbance at 260 nm (Normalized $A_{(260\ nm)}$) *versus* temperature (T (ºC)). A sigmoidal fitting of the data was applied for each compound and the melting temperature T_m (temperature with normalized absorbance of 0.5) was determined.

S4.5 Determination of the thermodynamic parameters

In order to determine the thermodynamics parameters enthalpy change (ΔH), entropy change (ΔS) and ΔG, the DNA-binding constants of the compounds were determined for three different temperatures (295 K, 303 K and 310 K) with equation S1 and the corresponding plots. The enthalpy change (ΔH) and the entropy change (ΔS) were calculated with the van't Hoff equation:

$$\ln(K_b) = -\frac{\Delta H}{RT} + \frac{\Delta S}{R} \qquad \text{(equation S4)}$$

where ΔH and ΔS can be determined from the plot of $\ln(K_b)$ *versus* (1/T), where - ΔH/R is the slope of the fitting line and ΔS/R is the intercept (R is the universal gas constant). In addition, ΔG was obtained from the Gibb's-Helmholtz equation:

$$\Delta G = \Delta H - T\cdot\Delta S \qquad \text{(equation S5)}$$

S5 Plasmid DNA cleavage experiments

The reaction mixtures (20 μL) containing supercoiled circular pBR322 plasmid DNA stock solution (Form I, 50 μM/base pair, ~500 ng), compounds, and Tris buffer (25 μM, pH 6.8) in Eppendorf PCR tubes were incubated for 30 min at 37 °C and centrifuged under aerobic conditions at room temperature. Afterwards, in the experiments that irradiation was used, the reaction mixtures contained in the Eppendorf PCR tubes, where irradiated at room temperature with UVB light (312 nm) for 30 min, at a distance of 15 cm, and in the case of UVA light (365 nm) and visible light (400 nm) for 2 h, at a distance of 10 cm.

After addition of the gel-loading buffer [6x Orange DNA Loading Dye 10 mM Tris-HCl (pH 7.6), 0.15% orange G, 0.03% xylene cyanol FF, 60% glycerol, and 60 mM EDTA, by Fermentas], the reaction mixtures were loaded on a 1% agarose gel with EB staining. The electrophoresis tank was attached to a power supply at a constant current (75 V for 30 min). The gel was visualized by the Mupid-ONE LED Illuminator and photographed by a Nikon

Digital Camera D3400. Quantification of DNA-cleaving activities was performed by integration of the optical density as a function of the band area using the program "Image J" available at the site http://rsb.info.nih.gov/ij/download.html.

The ss% and ds% damages were calculated according to the equations S6-S7:

$$ss\% = \frac{Form\ II}{(Form\ I + Form\ II + Form\ III)} \times 100 \qquad \text{(equation S6)}$$

$$ds\% = \frac{Form\ III}{(Form\ I + Form\ II + Form\ III)} \times 100 \qquad \text{(equation S7)}$$

where, as Form II we consider Form II of each series minus Form II of the irradiated control DNA and as Form I, we consider Form I of each series. The amount of supercoiled DNA was multiplied by factor of 1.43 to account for reduced EB intercalation into supercoiled DNA [10].

S6 Albumin-binding studies

S6.1 Interaction with albumins

In order to study the interaction of the compounds with the albumins, the compound was initially dissolved in DMSO (1 mM). Mixing of such solutions with the aqueous buffer solutions of the albumins used in the studies never exceeded 5% DMSO (v/v) in the final solution, which was needed due to low aqueous solubility of most compounds.

With the purpose to investigate if the compound can bind to carrier protein like serum albumins, albumin binding studies were carried out by tryptophan fluorescence quenching experiments using BSA or HSA (3 μM) in buffer (containing 15 mM trisodium citrate and 150 mM NaCl at pH 7.0). The quenching of the emission intensity of tryptophan residues of BSA at 344 nm or HSA at 342 nm was monitored using the compound as quenchers with increasing concentration [8]. The fluorescence emission spectra of the compound were also recorded with $\lambda_{excitation}$ = 295 nm; in case that an additional emission band appeared the albumin-fluorescence emission spectra were corrected by subtracting the spectra of the compound. The influence of the inner-filter effect on the measurements was evaluated by equation [11] :

$$I_{corr} = I_{meas} \times 10^{\frac{\varepsilon(\lambda_{exc})cd}{2}} \times 10^{\frac{\varepsilon(\lambda_{em})cd}{2}} \qquad \text{(equation S8)}$$

where I_{corr} = corrected intensity, I_{meas} = the measured intensity, c = the concentration of the quencher, d = the cuvette (1 cm), $\varepsilon_{(\lambda exc)}$ and $\varepsilon_{(\lambda em)}$ = the ε of the quencher at the excitation and the emission wavelength, respectively, as calculated from the UV-vis spectra of the compound [11].

The Stern-Volmer and Scatchard graphs are used to study the interaction of the compound with the albumins. According to the Stern-Volmer quenching equation (equation S2), where Io = initial tryptophan fluorescence intensity of albumin, I = tryptophan fluorescence intensity of BSA after the addition of the quencher, K_q = quenching constant, K_{SV} = Stern-Volmer constant, τ_o = average lifetime of albumin without the quencher, and, taking as fluorescence lifetime (τ_o) of tryptophan in albumin at around 10^{-8} s [8], K_{SV} (in M^{-1})

can be obtained by the slope of the diagram Io/I *versus* [compound] (Stern-Volmer plots), and subsequently the quenching constant (K_q, in $M^{-1}s^{-1}$) may be calculated from equation S3.

From the Scatchard equation:

$$\frac{\Delta I/\mathrm{Io}}{[Q]} = \mathrm{nK} - K\frac{\Delta \mathrm{I}}{\mathrm{Io}} \qquad \text{(equation S9)}$$

where n is the number of binding sites per albumin and K is the albumin-binding constant (K, in M^{-1}) is calculated from the slope in plots (ΔI/Io)/[compound] *versus* (ΔI/Io) and n is given by the ratio of y intercept to the slope [12].

S6.2 Competitive albumin-fluorescence studies with warfarin and ibuprofen

The competitive studies with warfarin or ibuprofen (as site-markers) [13] were performed by tryptophan fluorescence quenching experiments using a fixed concentration of the albumin and site markers (3 μM) in buffer (containing 15 mM trisodium citrate and 150 mM NaCl at pH 7.0). The fluorescence emission spectra were recorded in the presence of increasing amounts of the compounds as quenchers with an excitation wavelength of 295 nm. The Scatchard equation (equation S9) [12] and plots were applied on the corrected albumin-fluorescence emission spectra to determine the albumin-binding constant of the compounds in the presence of warfarin or ibuprofen.

S7 References

1. Tseng, H.J.; Lin, M.H.; Shiao, Y.J.; Yang, Y.C.; Chu, J.C.; Chen, C.Y.; Chen, Y.Y.; Lin, T.E.; Su, C.J.; Pan, S.L.; et al. Synthesis and Biological Evaluation of Acridine-Based Histone Deacetylase Inhibitors as Multitarget Agents against Alzheimer's Disease. *Eur J Med Chem* **2020**, *192*, 112193, doi:10.1016/j.ejmech.2020.112193.
2. Kontogiorgis, C.; Hadjipavlou-Litina, D. Biological Evaluation of Several Coumarin Derivatives Designed as Possible Anti-Inflammatory/Antioxidant Agents. *J Enzyme Inhib Med Chem* **2003**, *18*, 63-69, doi:10.1080/1475636031000069291.
3. Ruch, R.J.; Cheng, S.J.; Klaunig, J.E. Prevention of Cytotoxicity and Inhibition of Intercellular Communication by Antioxidant Catechins Isolated from Chinese Green Tea. *Carcinogenesis* **1989**, *10*, 1003-1008, doi:10.1093/CARCIN/10.6.1003.
4. Stathopoulou, M.E.K.; Banti, C.N.; Kourkoumelis, N.; Hatzidimitriou, A.G.; Kalampounias, A.G.; Hadjikakou, S.K. Silver Complex of Salicylic Acid and Its Hydrogel-Cream in Wound Healing Chemotherapy. *J Inorg Biochem* **2018**, *181*, 41-55, doi:10.1016/J.JINORGBIO.2018.01.004.
5. Milionis, I.; Banti, C.N.; Sainis, I.; Raptopoulou, C.P.; Psycharis, V.; Kourkoumelis, N.; Hadjikakou, S.K. Silver Ciprofloxacin (CIPAG): A Successful Combination of Chemically Modified Antibiotic in Inorganic-Organic Hybrid. *Journal of Biological Inorganic Chemistry* **2018**, *23*, 705-723, doi:10.1007/S00775-018-1561-9/FIGURES/8.
6. Banti, C.N.; Kapetana, M.; Papachristodoulou, C.; Raptopoulou, C.P.; Psycharis, V.; Zoumpoulakis, P.; Mavromoustakos, T.; Hadjikakou, S.K. Hydrogels Containing Water Soluble Conjugates of Silver(I) Ions with Amino Acids, Metabolites or Natural Products for

Non Infectious Contact Lenses. *Dalton Transactions* **2021**, *50*, 13712-13727, doi:10.1039/D1DT02158C.
7. Wolfe, A.; Shimer, G.H.; Meehan, T. Polycyclic Aromatic Hydrocarbons Physically Intercalate into Duplex Regions of Denatured DNA. *Biochemistry* **1987**, *26*, 6392-6396, doi:10.1021/bi00394a013.
8. Lakowicz, J.R. *Principles of Fluorescence Spectroscopy*; Lakowicz, J.R., Ed.; Springer US: Boston, MA, 2006; ISBN 978-0-387-31278-1.
9. Heller, D.P.; Greenstock, C.L. Fluorescence Lifetime Analysis of DNA Intercalated Ethidium Bromide and Quenching by Free Dye. *Biophys Chem* **1994**, *50*, 305-312, doi:10.1016/0301-4622(93)E0101-A.
10. Papastergiou, A.; Perontsis, S.; Gritzapis, P.; Koumbis, A.E.; Koffa, M.; Psomas, G.; Fylaktakidou, K.C. Evaluation of O-Alkyl and Aryl Sulfonyl Aromatic and Heteroaromatic Amidoximes as Novel Potent DNA Photo-Cleavers. *Photochemical and Photobiological Sciences* **2016**, *15*, 351-360, doi:10.1039/c5pp00439j.
11. Stella, L.; Capodilupo, A.L.; Bietti, M. A Reassessment of the Association between Azulene and [60]Fullerene. Possible Pitfalls in the Determination of Binding Constants through Fluorescence Spectroscopy. *Chemical Communications* **2008**, 4744-4746, doi:10.1039/B808357F.
12. Wang, Y.-Q.; Zhang, H.-M.; Zhang, G.-C.; Tao, W.-H.; Tang, S.-H. Interaction of the Flavonoid Hesperidin with Bovine Serum Albumin: A Fluorescence Quenching Study. *J Lumin* **2007**, *126*, 211-218, doi:10.1016/j.jlumin.2006.06.013.
13. Lazou, M.; Tarushi, A.; Gritzapis, P.; Psomas, G. Transition Metal Complexes with a Novel Guanine-Based (E)-2-(2-(Pyridin-2-Ylmethylene)Hydrazinyl)Quinazolin-4(3H)-One: Synthesis, Characterization, Interaction with DNA and Albumins and Antioxidant Activity. *J Inorg Biochem* **2020**, *206*, 111019, doi:10.1016/j.jinorgbio.2020.111019.
14. de Meulenaer, J.; Tompa, H. The Absorption Correction in Crystal Structure Analysis. *Acta Crystallogr* **1965**, *19*, 1014-1018, doi:10.1107/S0365110X65004802.

TABLES

Table S1. Experimental crystallographic details for complex **1**.

	Complex **1**
Crystal data	
Chemical formula	$C_{32}H_{42}Br_2CoN_2O_{10}$
M_r	833.43
Crystal system, space group	Triclinic, *P*-1
Temperature (K)	295
a, *b*, *c* (Å)	7.337 (3), 8.707 (3), 15.276 (7)
α, β, γ (°)	85.25 (3), 80.78 (3), 71.21 (2)
V (Å^3)	911.4 (7)
Z	1
Radiation type	Mo *K*α
μ (mm^{-1})	2.72
Crystal size (mm)	0.24 × 0.23 × 0.17
Data collection	
Diffractometer	Bruker Kappa Apex2
Absorption correction	Numerical, Analytical Absorption [14]
T_{min}, T_{max}	0.54, 0.63
No. of measured reflections	15247
No. of independent reflections	3522
No. of observed reflections [$I > 2.0\sigma(I)$]	2812
R_{int}	0.021
$(\sin \theta/\lambda)_{max}$ (Å^{-1})	0.620
Refinement	
$R[F^2 > 2\sigma(F^2)]$, $wR(F^2)$, S	0.032, 0.065, 1.00
No. of reflections	2812
No. of parameters	214
H-atom treatment	H-atom parameters constrained
$\Delta\rho_{max}$, $\Delta\rho_{min}$ (e Å^{-3})	0.42, -0.31

Table S2. Hydrogen bonds (lengths in Å, angles in °) for complex **1**.

D*—H···*A	***D*—H (Å)**	**H···*A* (Å)**	***D*···*A* (Å)**	***D*—H···*A* (°)**
O3—H31···O2	0.84	1.76	2.593 (4)	171
N1—H11···O1	0.86	1.94	2.647 (4)	138
O4—H165···O5	0.85	1.80	2.658 (4)	180
O5—H166···O2^{ii}	0.81	1.95	2.759 (4)	170

Symmetry code: (ii) *x*-1, *y*, *z*.

Table S3. Thermodynamic parameters of the compounds for the interaction with CT DNA at different temperatures (295 K, 303 K and 310 K).

Compound	T (K)	K_b (M^{-1})	ΔG (kcal M^{-1})	ΔH (kcal M^{-1})	ΔS (cal M^{-1} K^{-1})
4′-Br-fenH	295	4.42×10^5	-2.72		
294 nm	303	1.18×10^5	-3.04	+19.57	+75.34
	310	1.79×10^6	-3.85		
Complex **1**	295	8.90×10^5	-3.51		
304 nm	303	9.21×10^5	-3.60	+0.59	+13.79
	310	9.81×10^5	-3.69		
Complex **2**	295	6.73×10^5	-3.44		
285 nm	303	9.58×10^5	-3.61	+7.33	+36.16
	310	2.28×10^6	-3.91		
Complex **3**	295	7.92×10^5	-3.46		
286 nm	303	8.69×10^5	-3.56	+0.89	+14.72
	310	9.38×10^5	-3.68		
Complex **4**	295	9.92×10^5	-3.51		
297 nm	303	4.10×10^6	-3.98	+9.24	+43.36
	310	5.59×10^6	-4.15		
Complex **5**	295	3.61×10^5	-3.26		
285 nm	303	4.75×10^5	-3.42	+2.79	+20.49
	310	6.14×10^5	-3.56		
Complex **6**	295	2.00×10^6	-3.11		
307 nm	303	2.18×10^6	-3.21	+0.62	+12.65
	310	2.25×10^6	-3.30		
Complex **7**	295	1.56×10^6	-3.63		
311 nm	303	1.64×10^6	-3.74	+0.67	+14.58
	310	1.78×10^6	-3.85		
Complex **8**	295	1.21×10^6	-3.56		
306 nm	303	1.48×10^6	-3.71	+2.95	+22.04
	310	2.14×10^6	-3.90		
Complex **9**	295	9.37×10^5	-3.50		
308 nm	303	1.88×10^6	-3.78	+4.92	+28.58
	310	2.36×10^6	-3.92		

FIGURES

Figure S1. ^{1}H NMR spectrum of 4′-Br-fenH in DMSO-d_6.

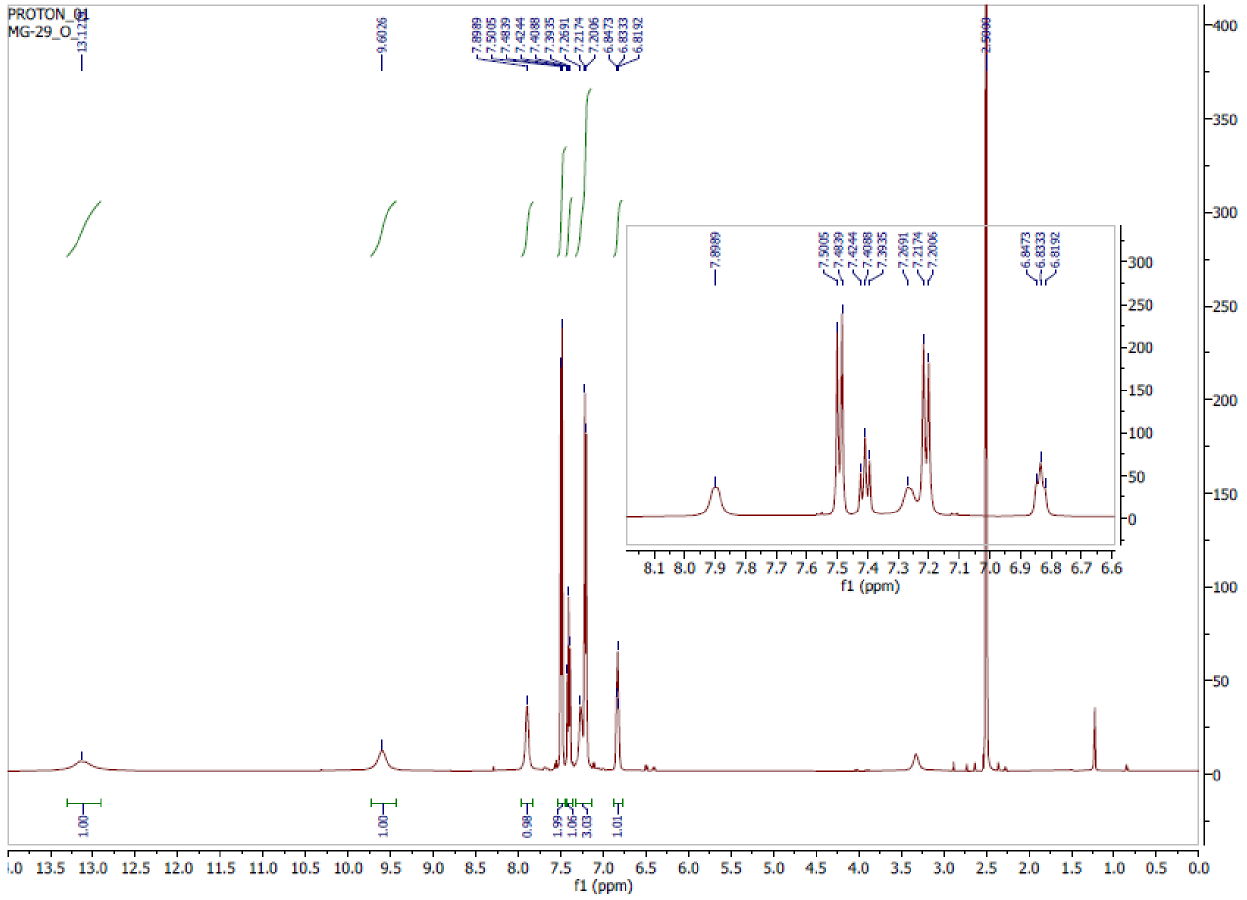

Figure S2. IR spectra (ATR) of the compounds.

4′-Br-fenH

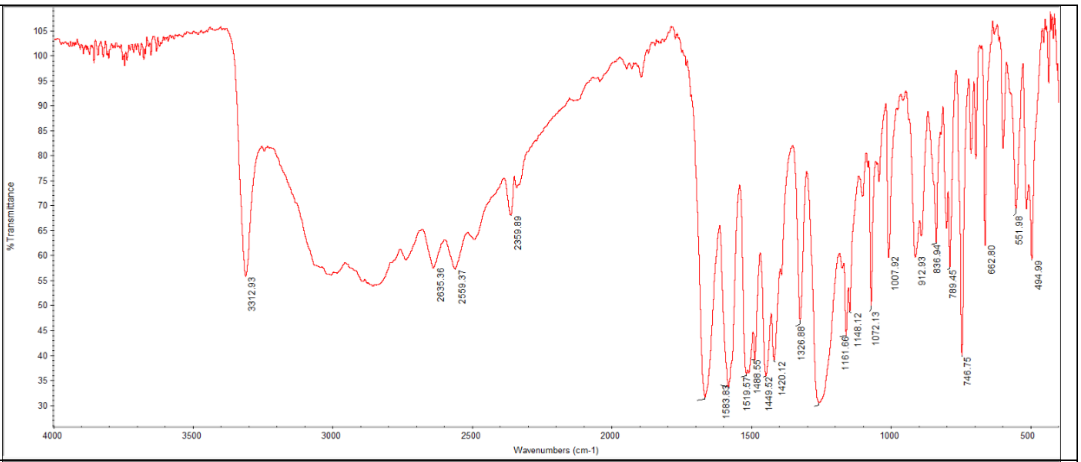

Complex **1**

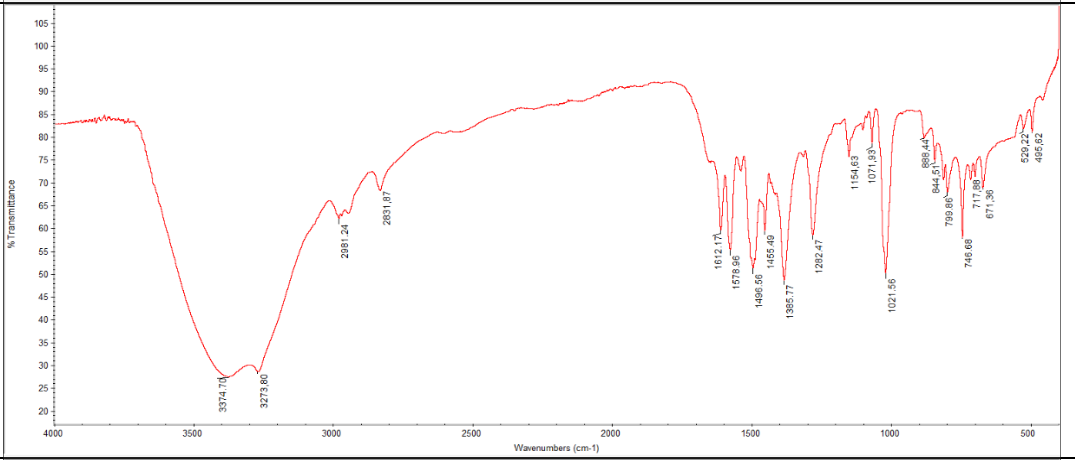

Complex **2**

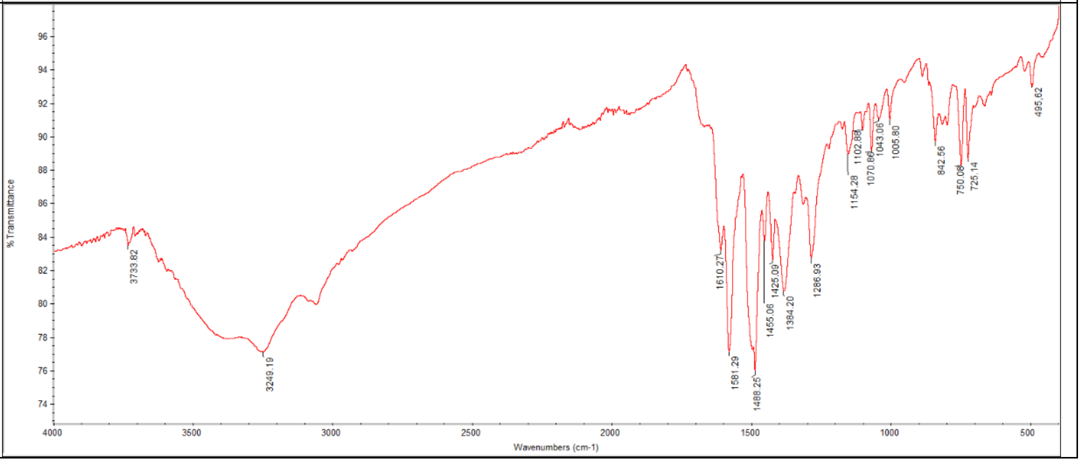

Complex **3**

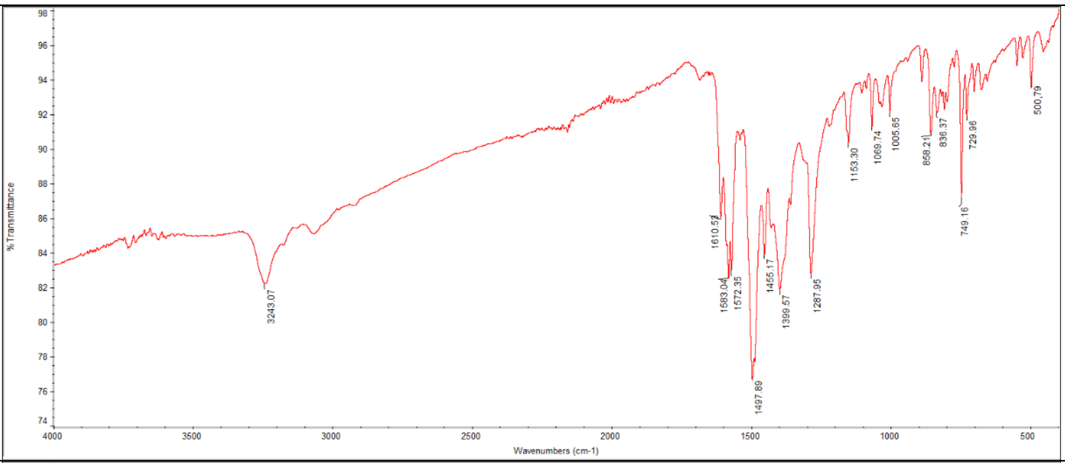

Complex **4**

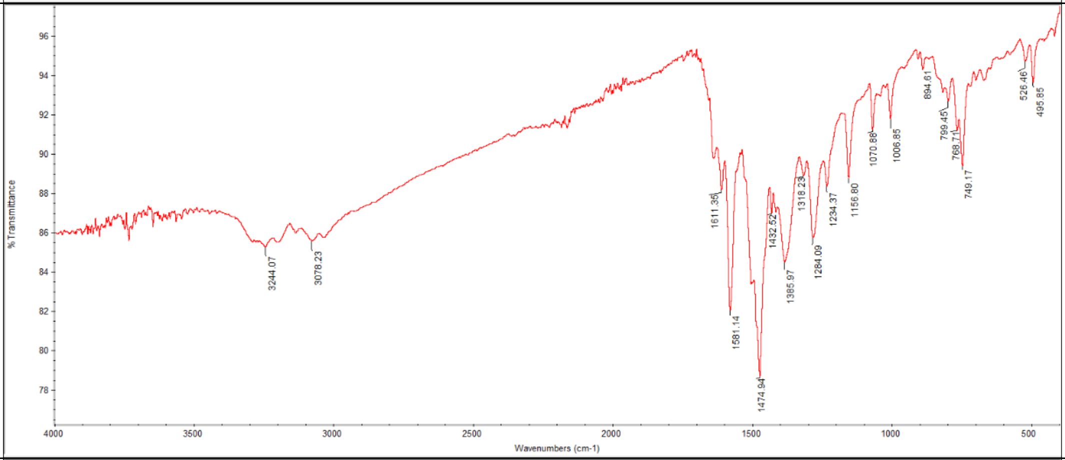

Complex **5**

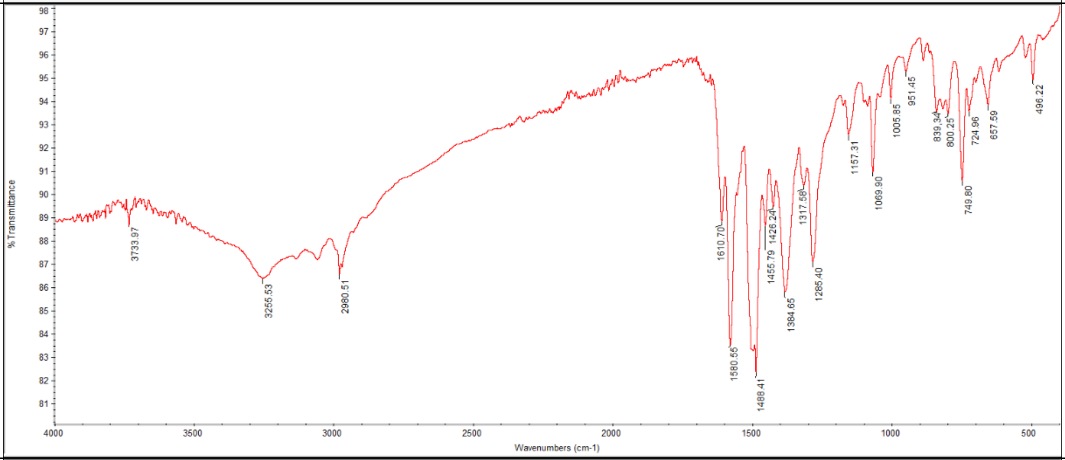

Complex **6**

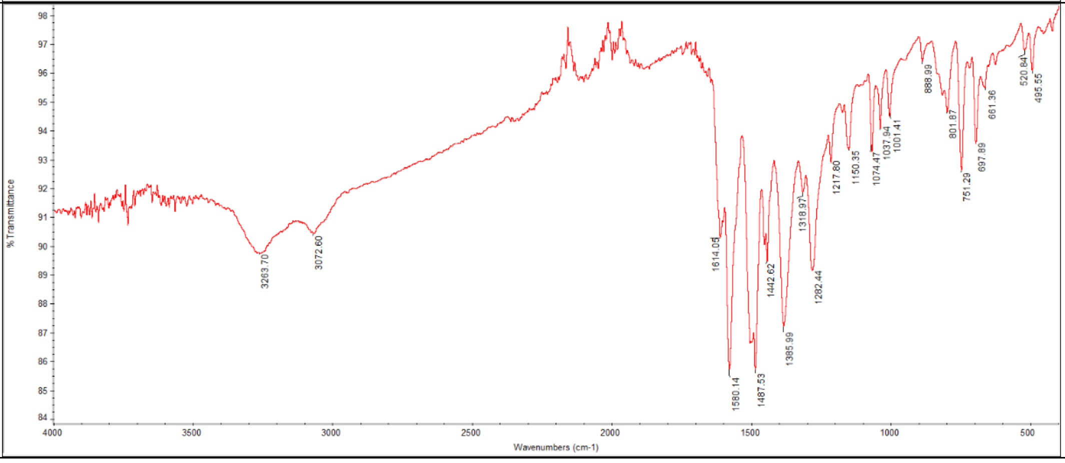

Complex **7**

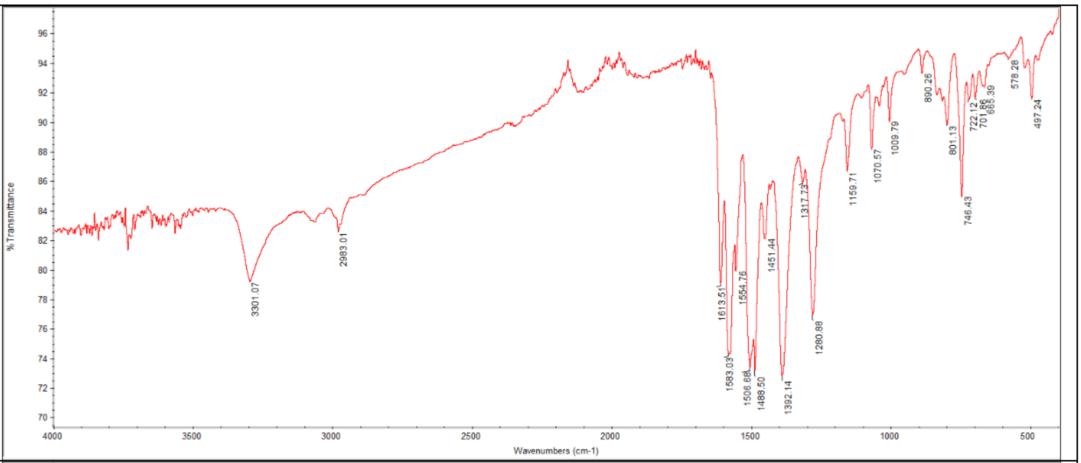

Complex **8**

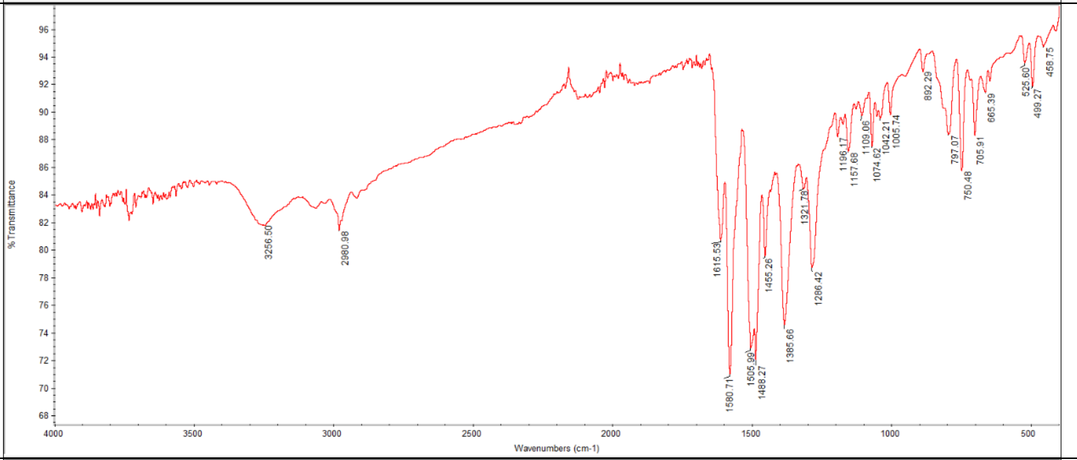

Complex **9**

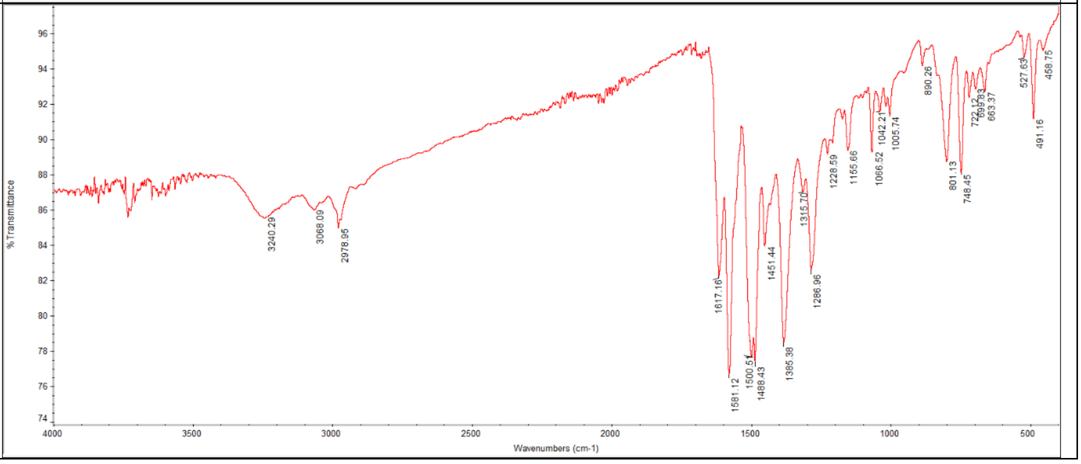

Figure S3. Mass spectra recorded for complexes **1-9**.
Conditions: Solvent: MeOH/MeCN Mixture. ESI(+) or ESI(-) or HRMS as denoted in parenthesis.

Complex **1**

(ESI(+) MS)

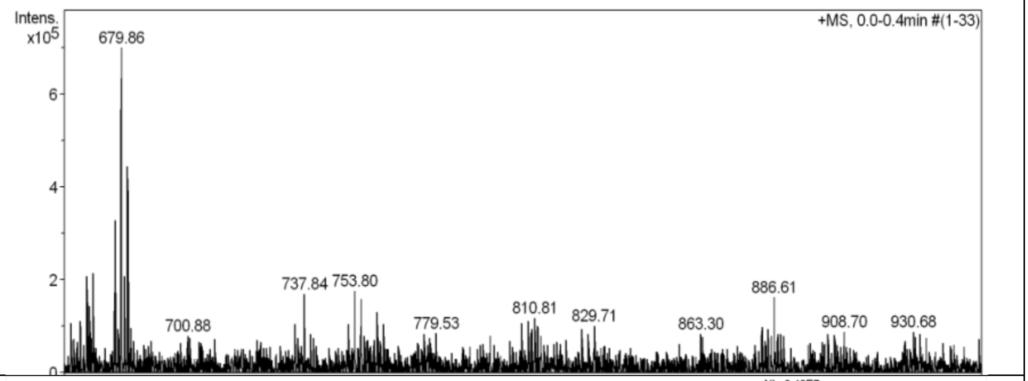

Complex **2**

(HRMS(+))

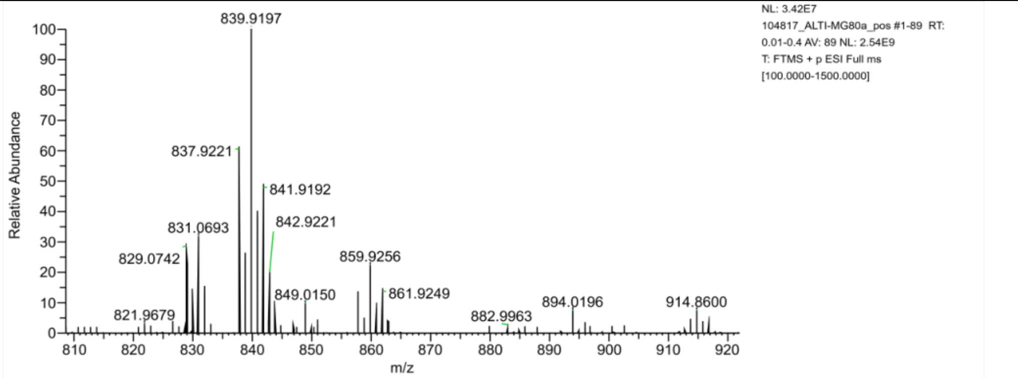

Complex **3**

(HRMS(+))

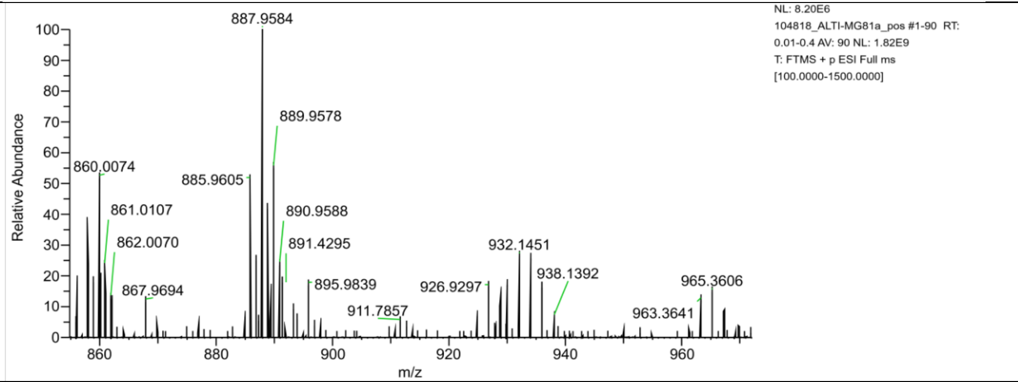

Complex **4**

(ESI(-) MS)

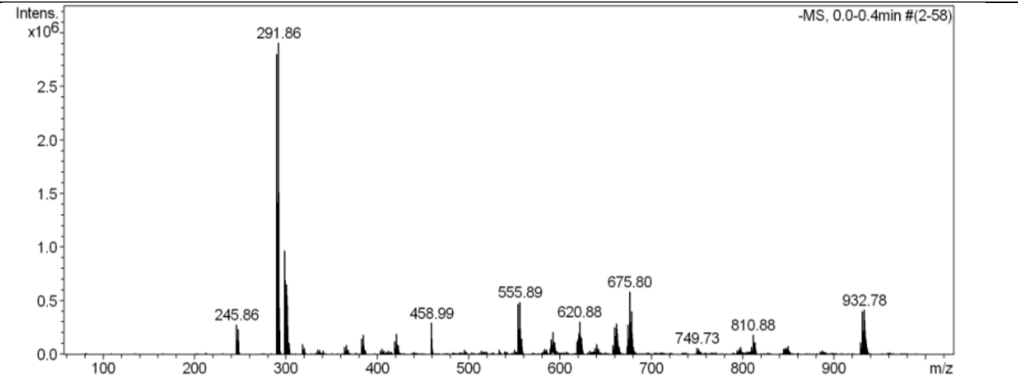

Complex **5**

(ESI(-) MS)

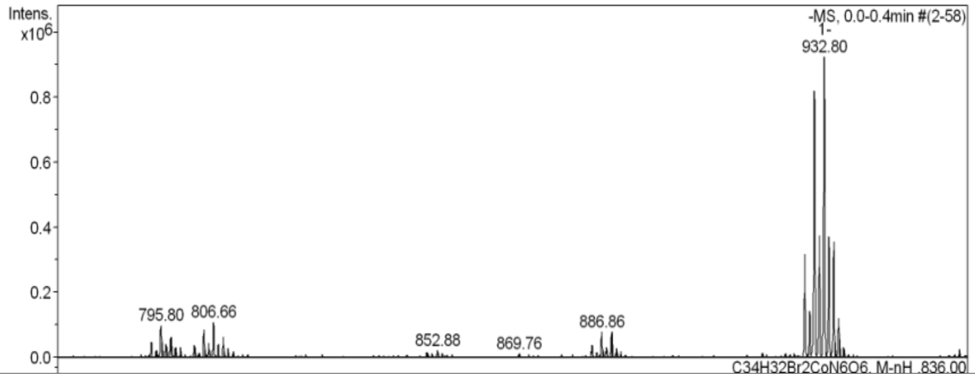

Complex **6**

(ESI(+) MS)

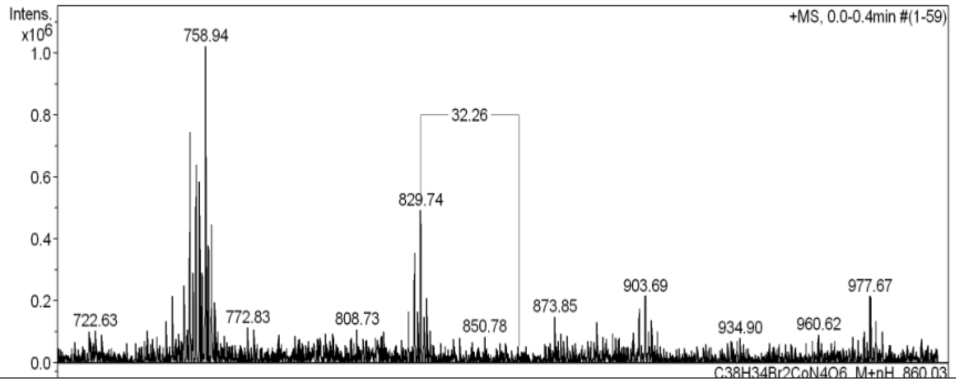

Complex **7**

(ESI(+) MS)

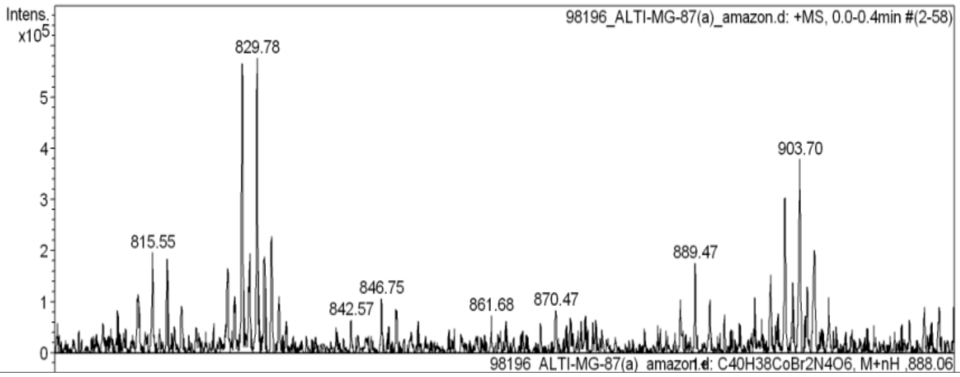

Complex **8**

(ESI(+) MS)

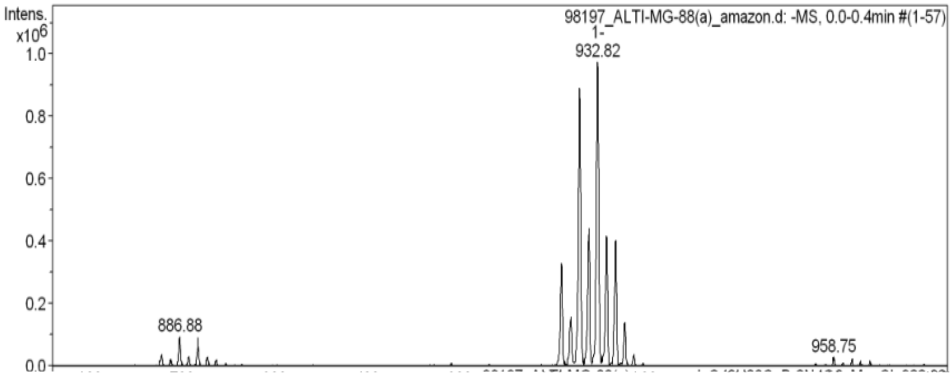

(ESI(-) MS)

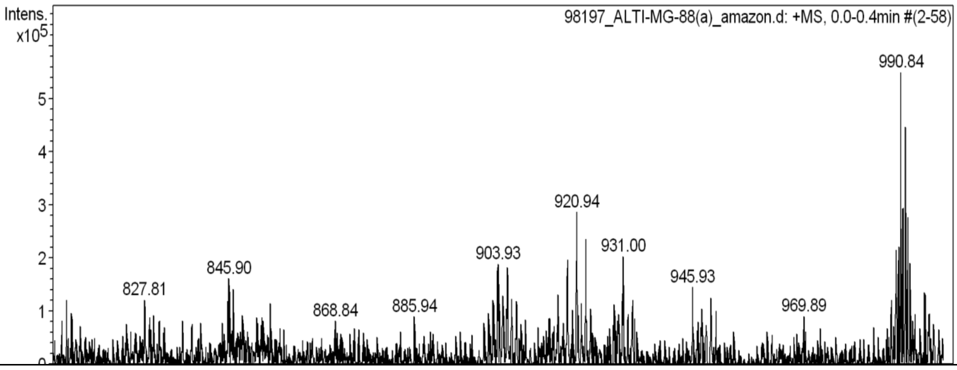

Complex **9**<br><br>(ESI(-) MS)	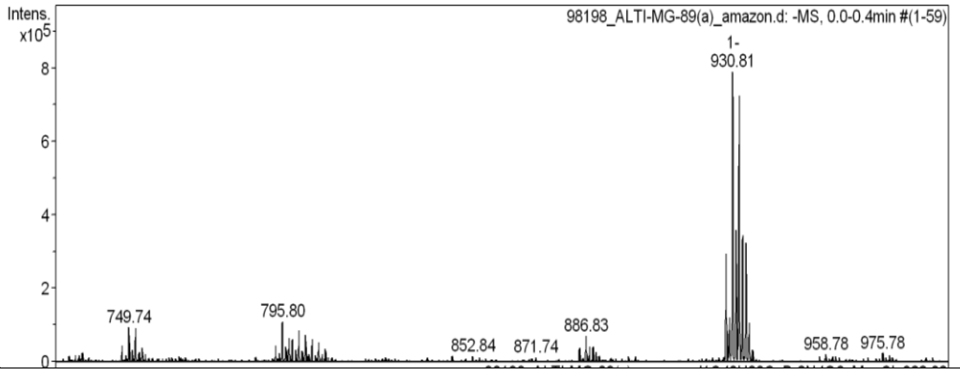

Figure S4. Visible spectra recorded for complexes **4** and **9** in the absence and presence of buffer solution for two different time-intervals.

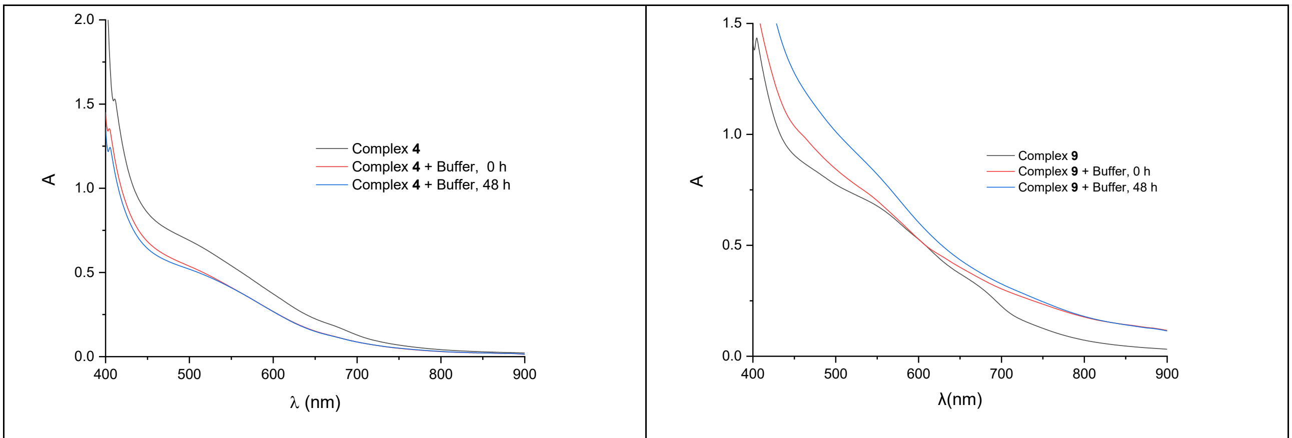

Complex **4**	Complex **9**

Figure S5: Proposed structures for complexes **2-9.**

Complex **2** (X = H)<br>Complex **3** (X = Me)	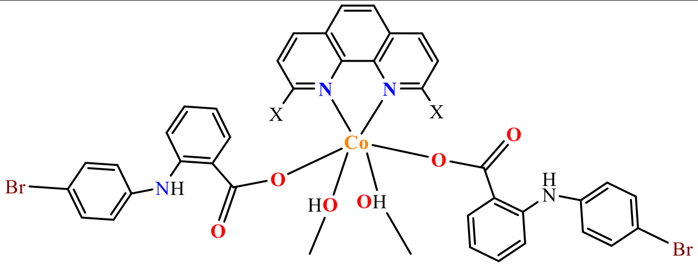
Complex **4**	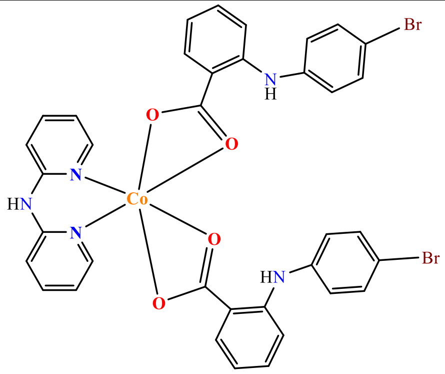
Complex **5**	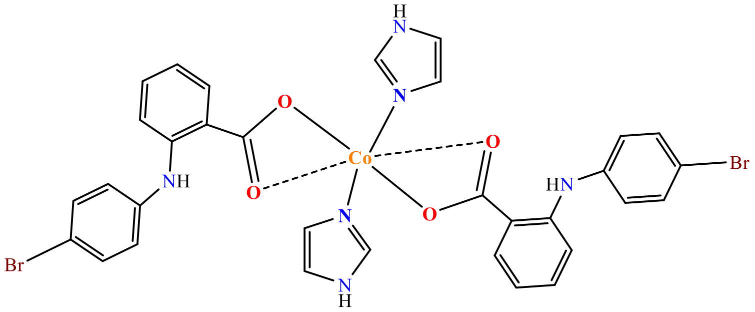
Complex **6** ($X_1 = X_2 = X_3$ = H)<br>Complex **7** (X_1 = Me, $X_2 = X_3$ = H)<br>Complex **8** (X_2 = Me, $X_1 = X_3$ = H)<br>Complex **9** (X_3 = Me, $X_1 = X_2$ = H)	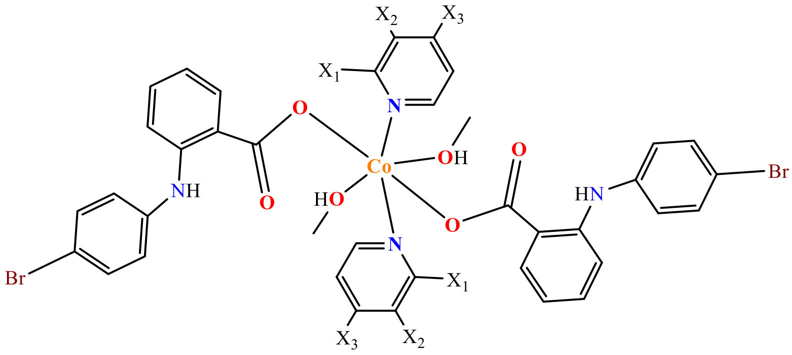

Figure S6. Minimum bactericidal concentration 4′-Br-fenH and its complexes **1-9** against *E. coli, S. epidermidis* and *S. aureus*.

**4′-Br-fenH**	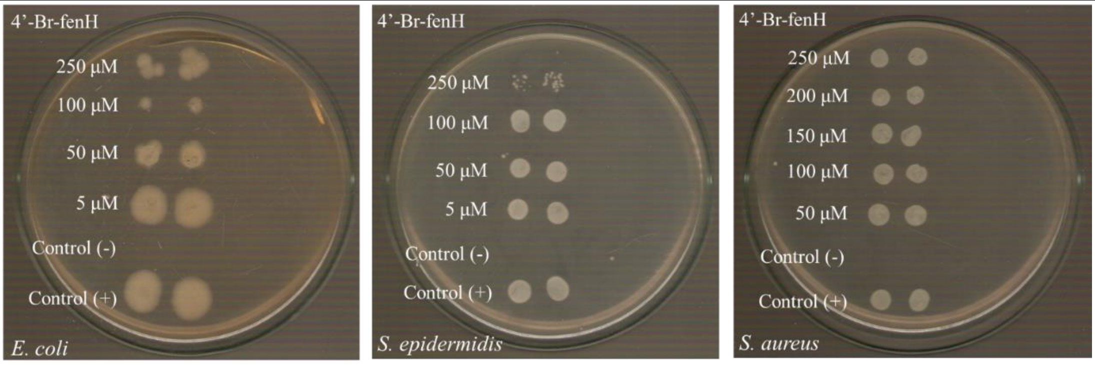
Complex **1**	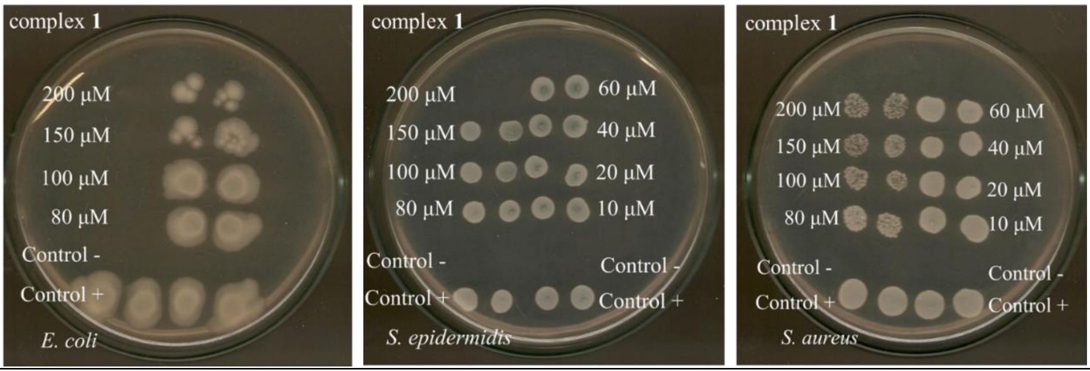
Complex **2**	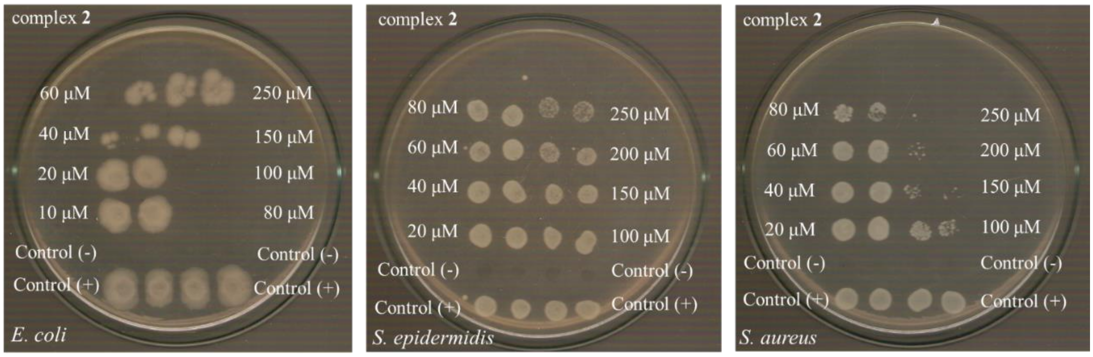
Complex **3**	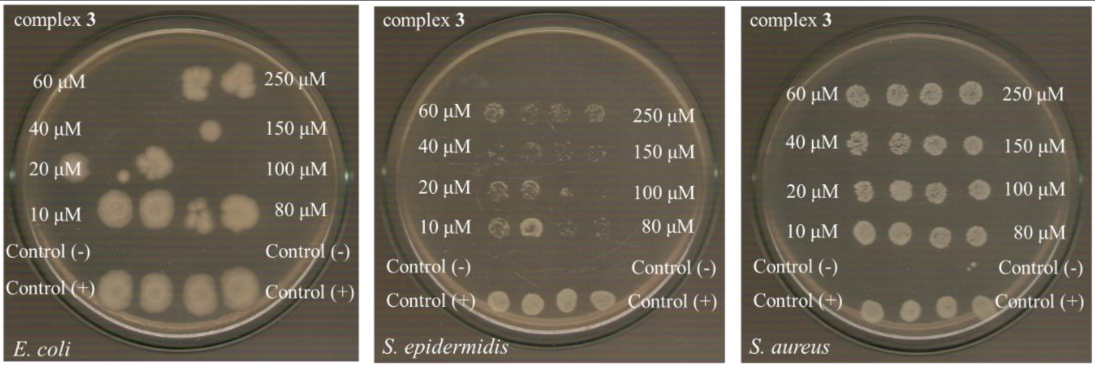

Complex **4**	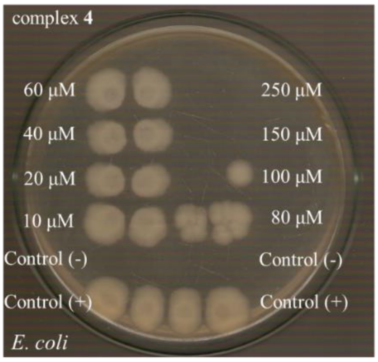 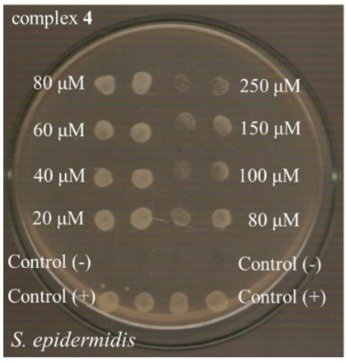 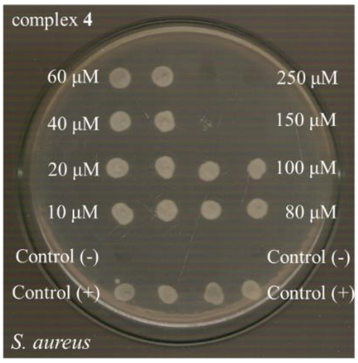
Complex **5**	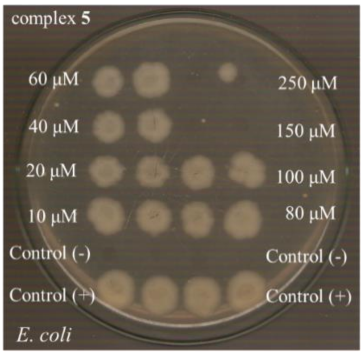 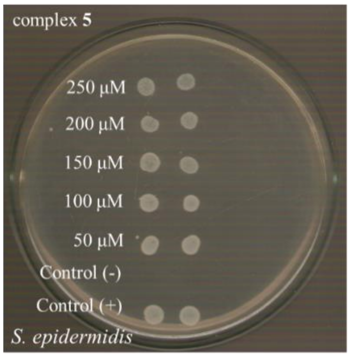 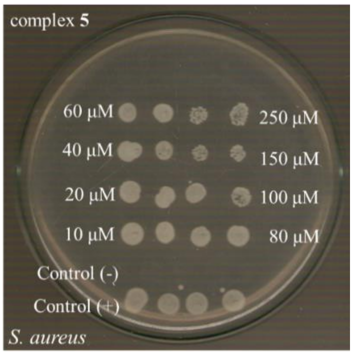
Complex **6**	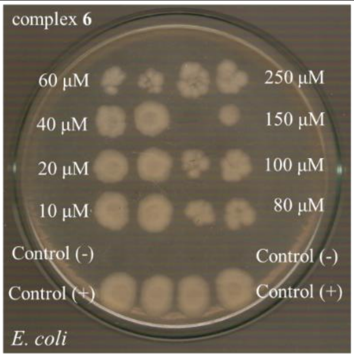 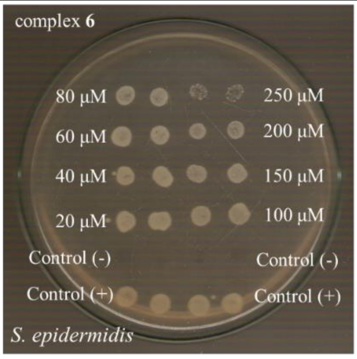 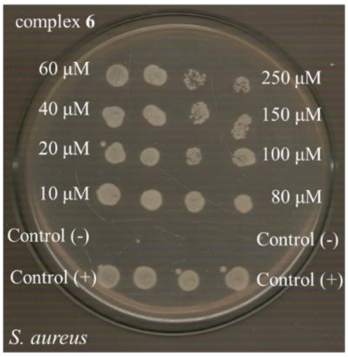
Complex **7**	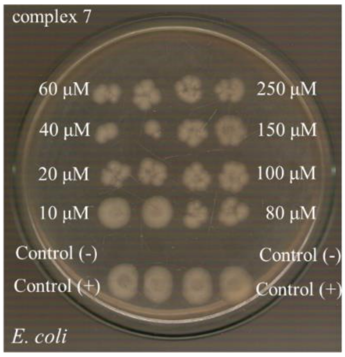 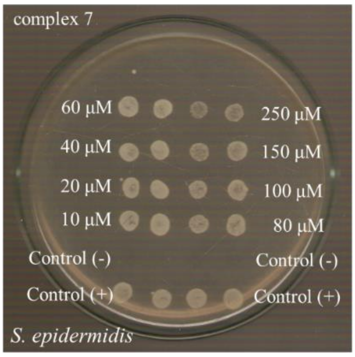 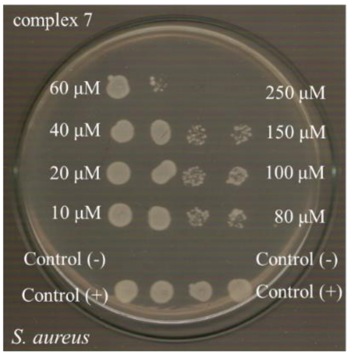
Complex **8**	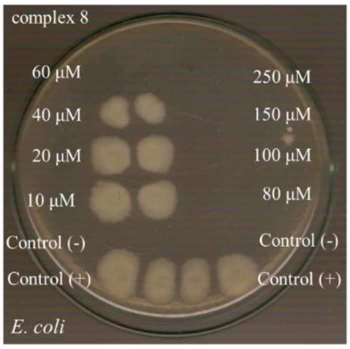 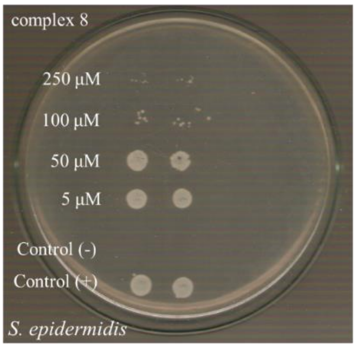 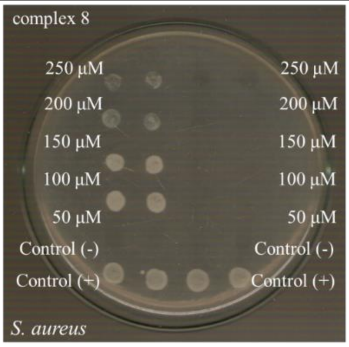

Complex **9**	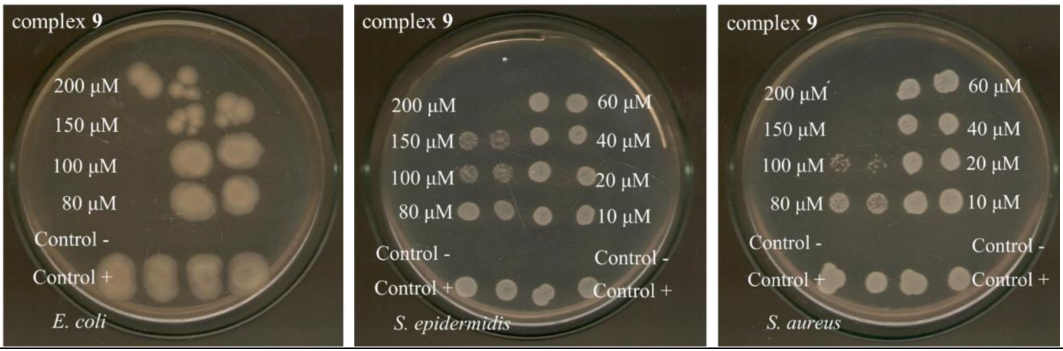

complex 9
200 μM
150 μM
100 μM
80 μM
Control -
Control +
E. coli
complex 9
200 μM
150 μM
100 μM
80 μM
60 μM
40 μM
20 μM
10 μM
Control -
Control +
Control -
Control +
S. epidermidis
complex 9
200 μM
150 μM
100 μM
80 μM
60 μM
40 μM
20 μM
10 μM
Control -
Control +
Control -
Control +
S. aureus

Figure S7. IZs which are developed in agar plates of *P. aeruginosa, E. coli, S. epidermidis* and *S. aureus* by 4′-Br-fenH and its complexes **1-9** and at 1 mM.

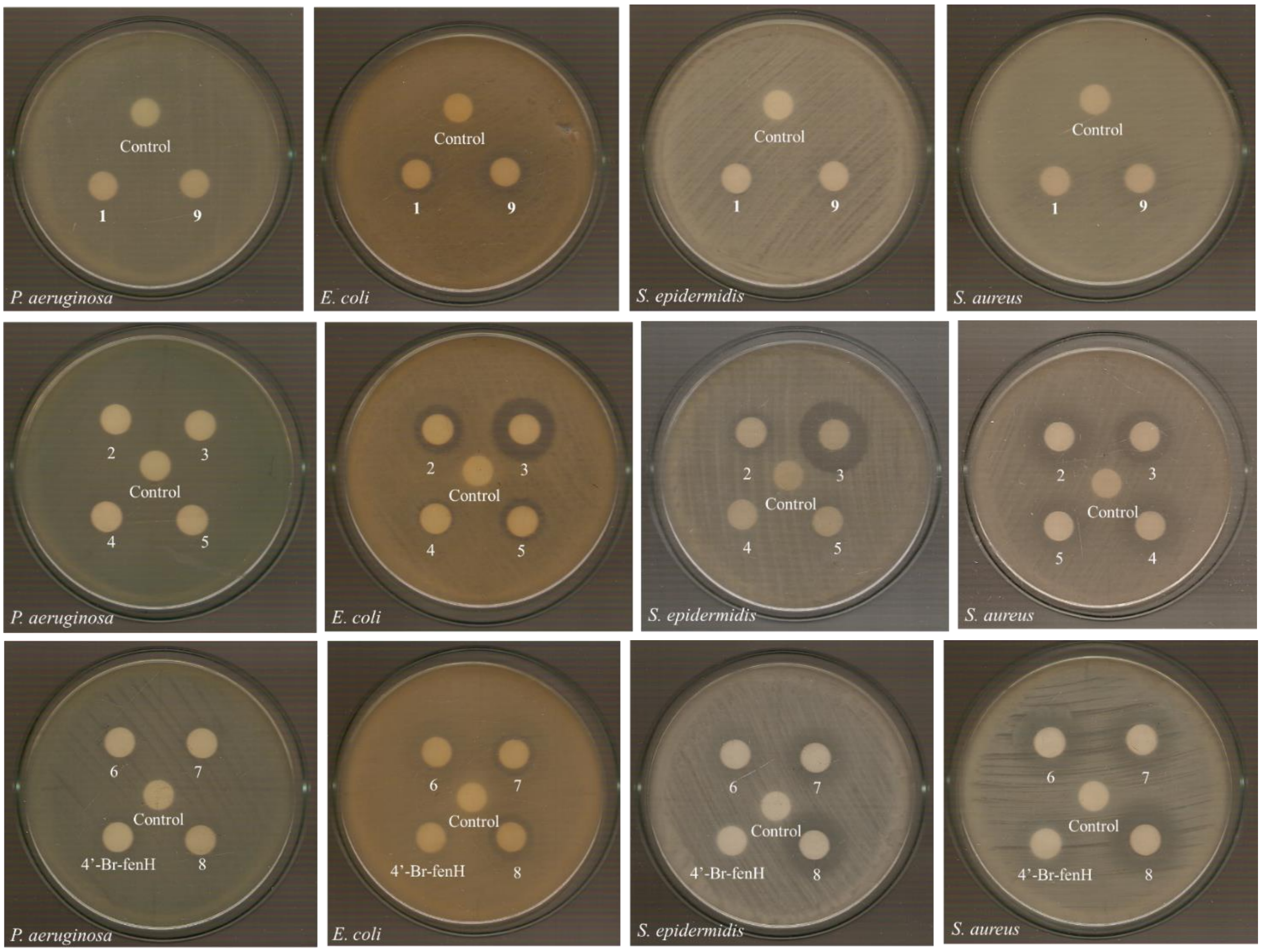

Figure S8. UV-vis spectra of a DMSO solution of the compounds (4′-Br-fenH and its complexes **1-9**) in the presence of increasing amounts of CT DNA.
The concentrations of the solution of the compounds are given in parentheses. The arrows show the changes upon increasing amounts of CT DNA.

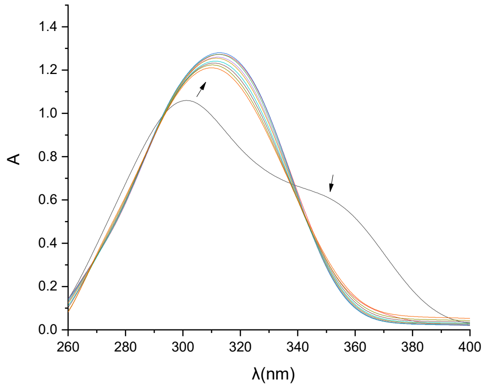

4′-Br-fenH (100 μM)

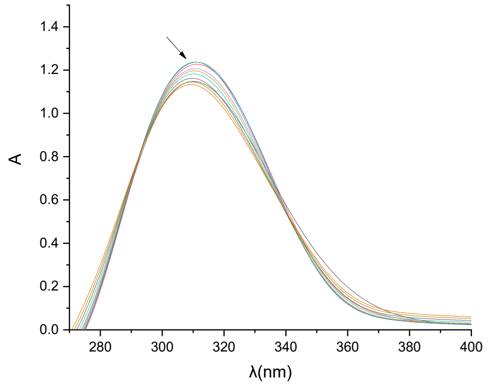

Complex **1** (60 μM)

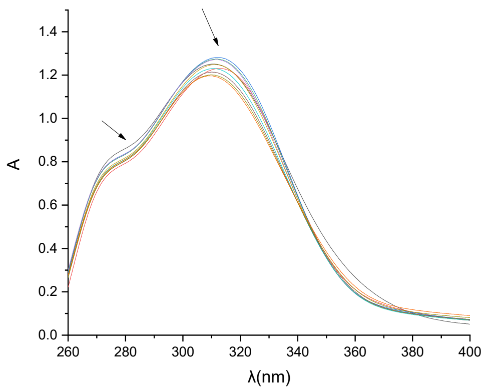

Complex **2** (50 μM)

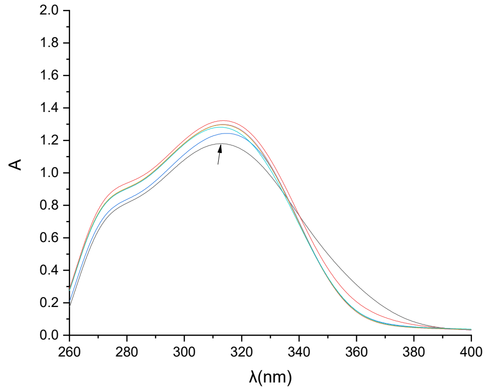

Complex **3** (50 μM)

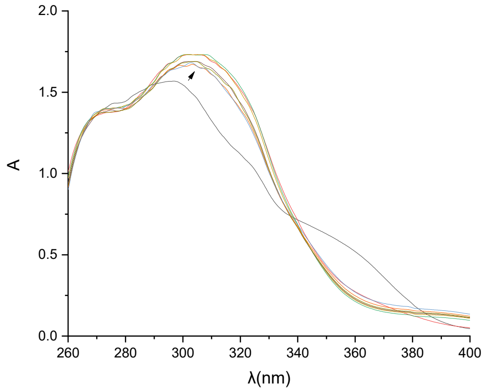

Complex **4** (40 μM)

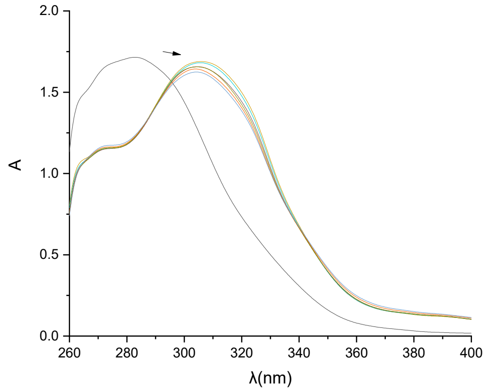

Complex **5** (25 μM)

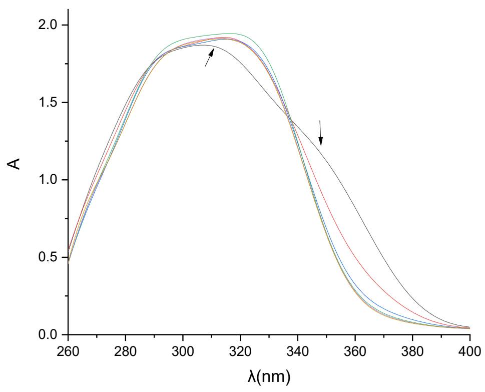

Complex **6** (100 μM)

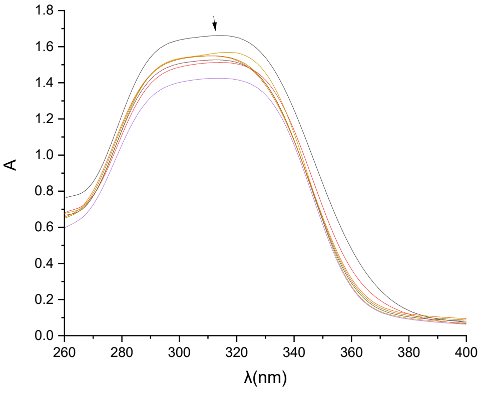

Complex **7** (100 μM)

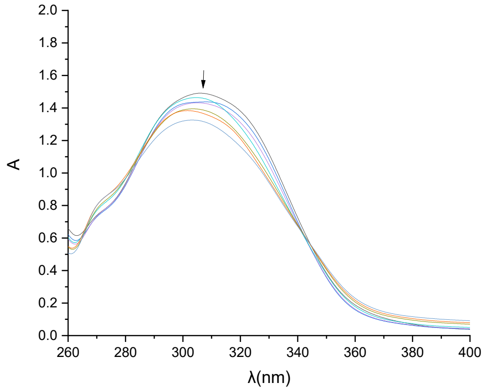

Complex **8** (50 μM)

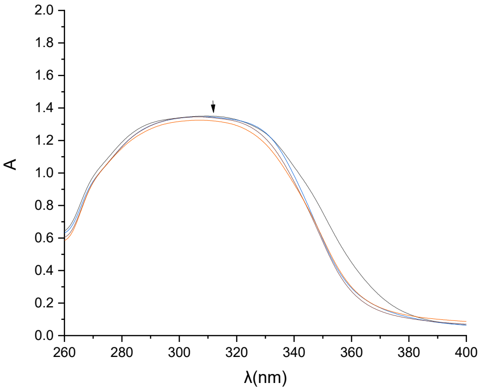

Complex **9** (100 μM)

Figure S9. Plots of $\frac{[DNA]}{(\varepsilon_A - \varepsilon_f)}$ *versus* [DNA] for the compounds.

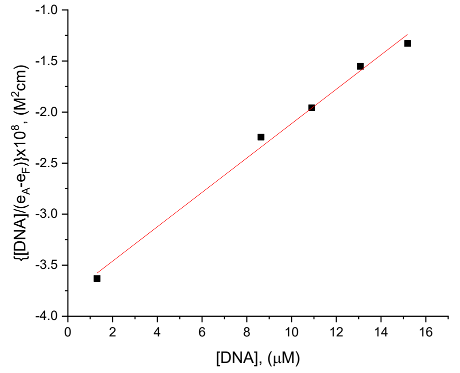

4′-Br-fenH

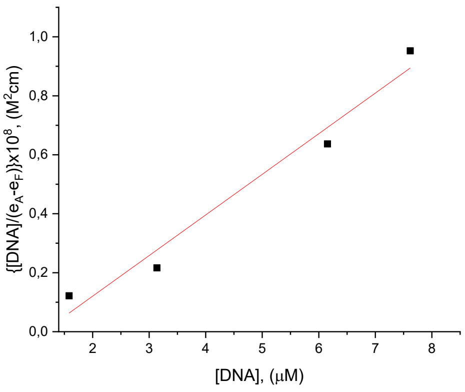

Complex **1**

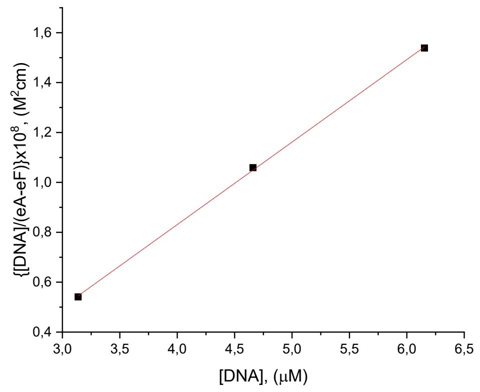

Complex **2**

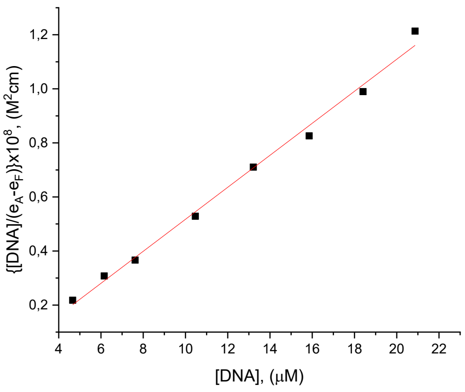

Complex **3**

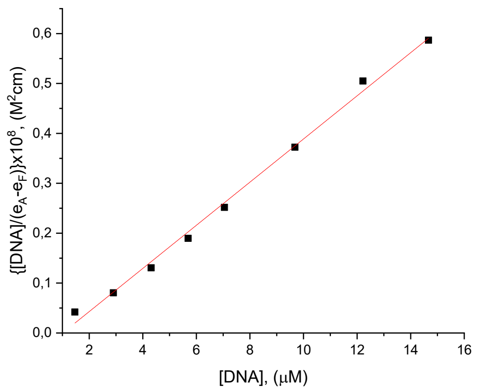

Complex **4**

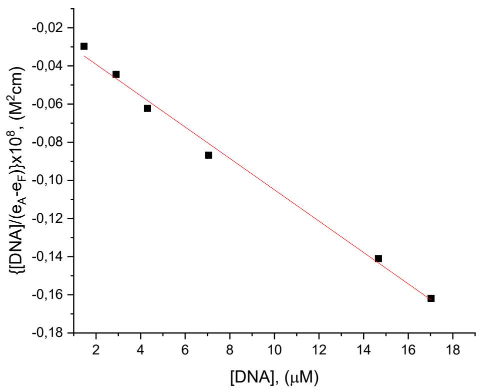

Complex **5**

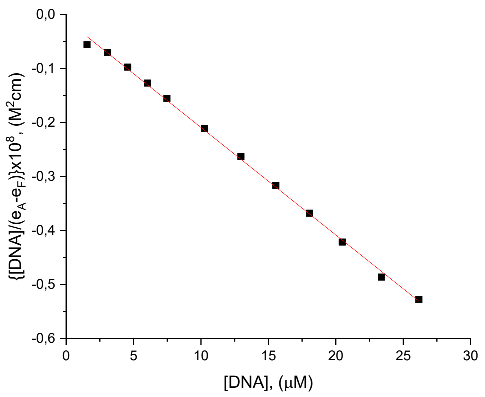

Complex **6**

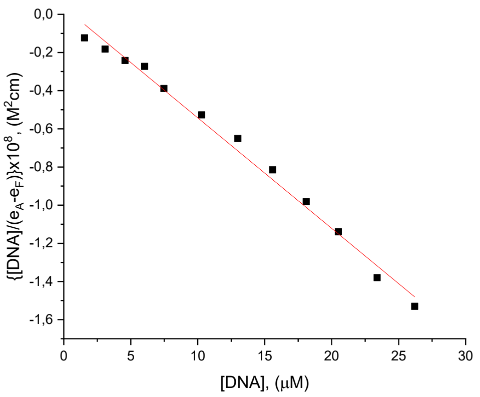

Complex **7**

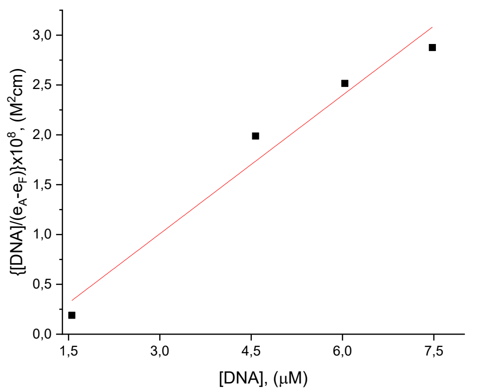

Complex **8**

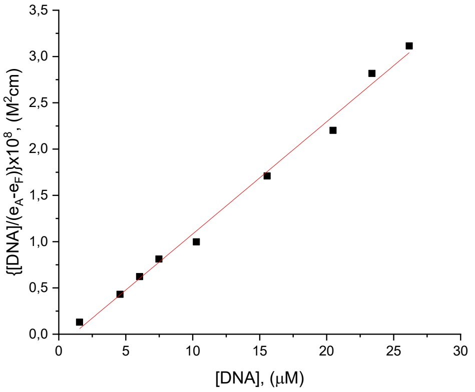

Complex **9**

Figure S10. Fluorescence emission spectra for EB-DNA in buffer solution in the absence and presence of increasing amounts of the compounds.

Conditions: $\lambda_{excitation}$ = 540 nm. [EB] = 40 μM. [DNA] = 45 μM. Buffer solution: 150 mM NaCl and 15 mM trisodium citrate at pH 7.0.

The arrow shows the changes of intensity upon increasing amounts of the compound.

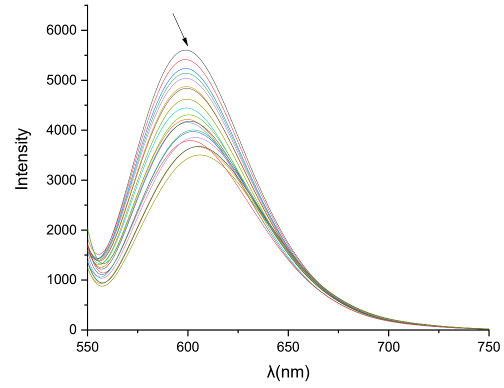

4′-Br-fenH

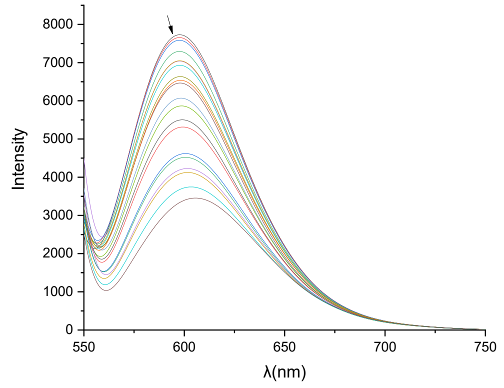

Complex **1**

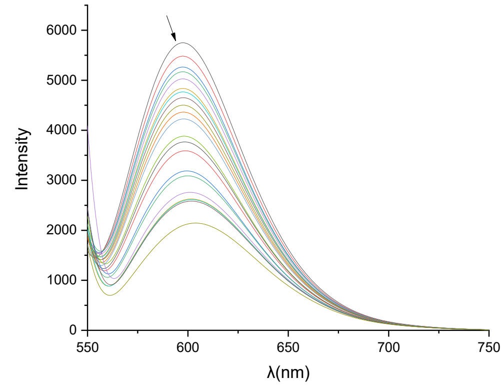

Complex **2**

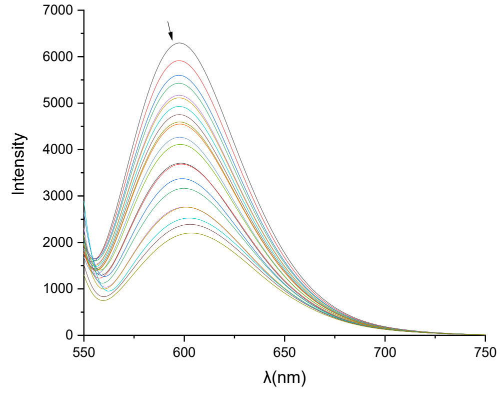

Complex **3**

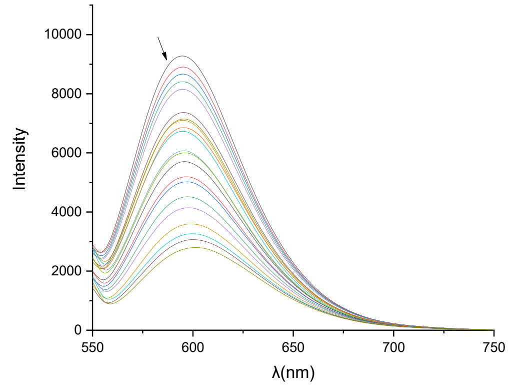

Complex **4**

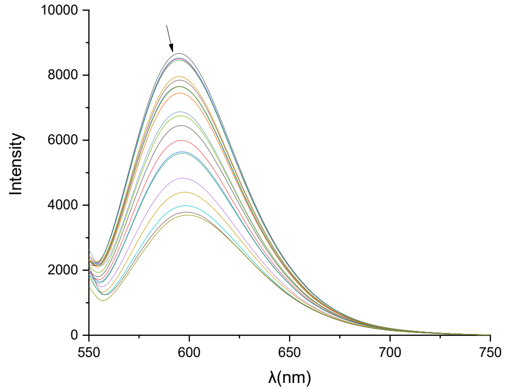

Complex **5**

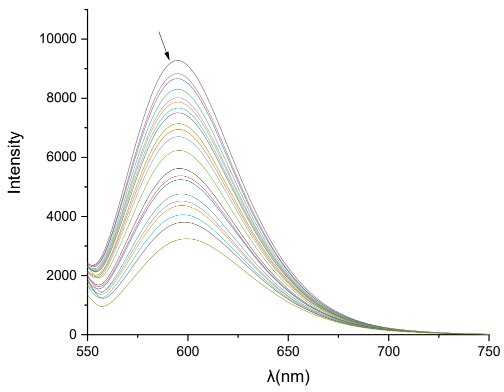

Complex **6**

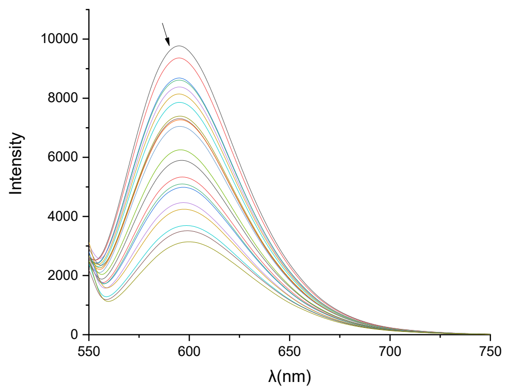

Complex **7**

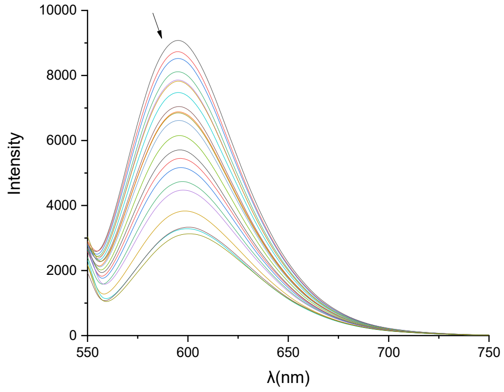

Complex **8**

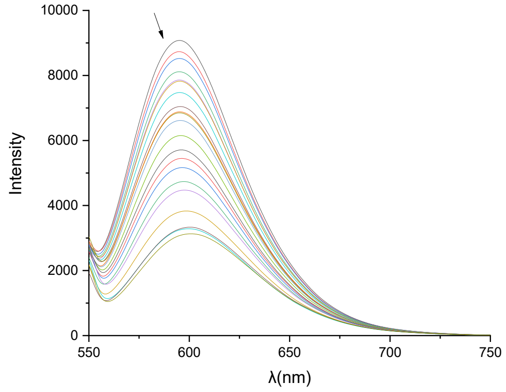

Complex **9**

Figure S11. Stern-Volmer plots of the EB-DNA quenching experiments upon addition of the compounds.

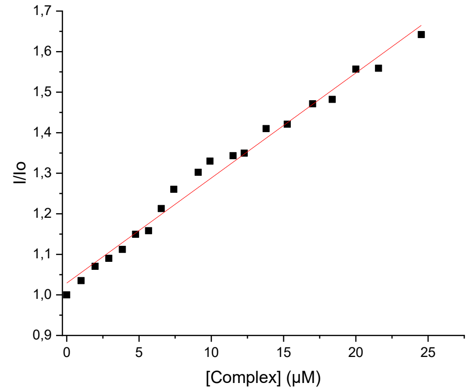

4′-Br-fenH

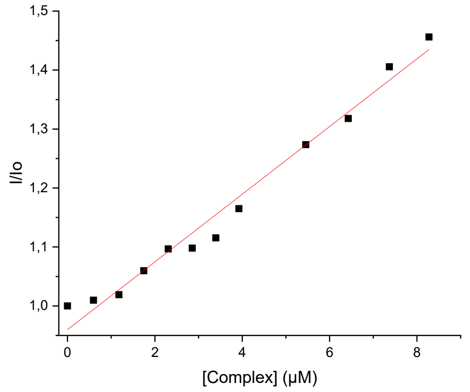

Complex **1**

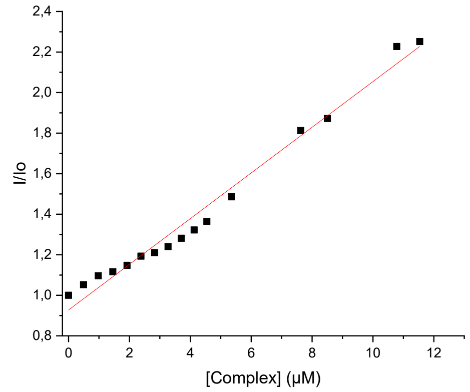

Complex **2**

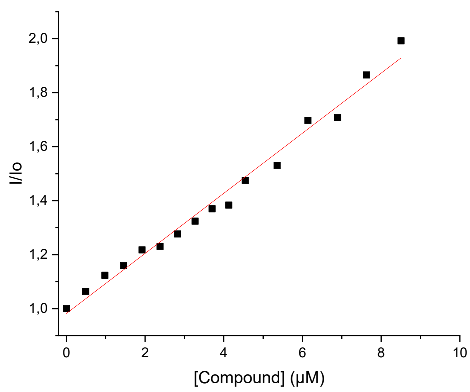

Complex **3**

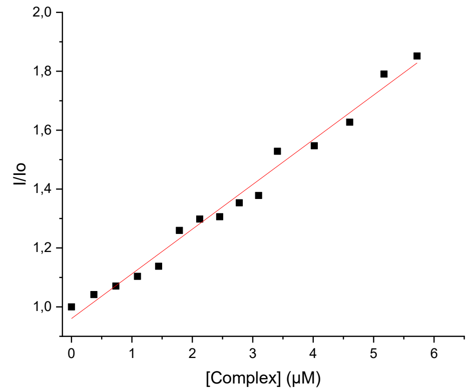

Complex **4**

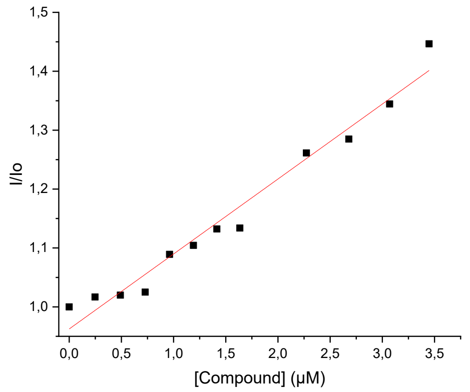

Complex **5**

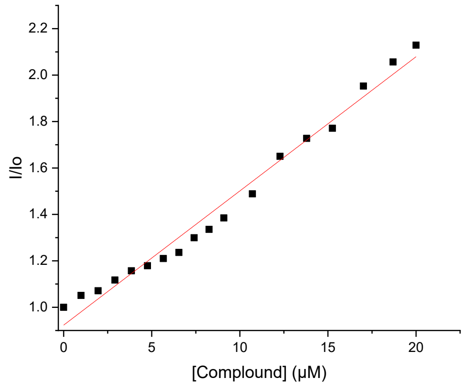

Complex **6**

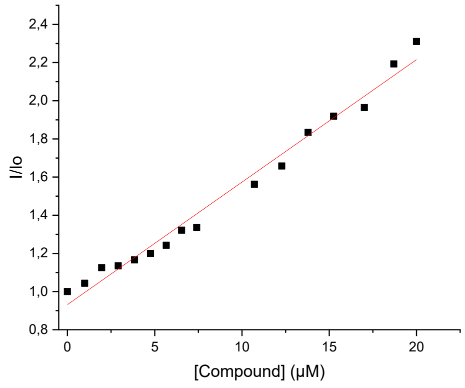

Complex **7**

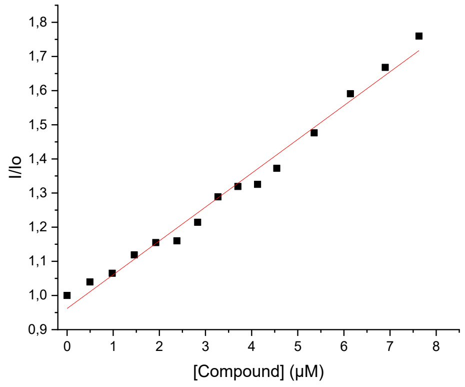

Complex **8**

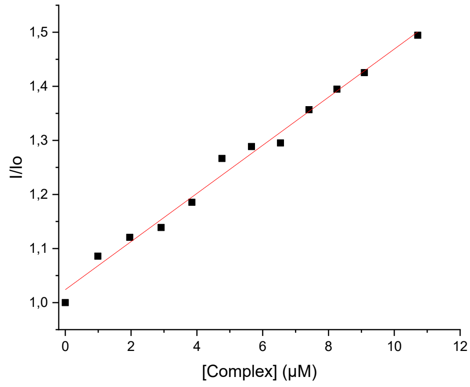

Complex **9**

Figure S12. Thermal melting profile of CT DNA in the absence or presence of the compounds.
[CT DNA] = 1.25×10^{-4} M; [Compound] = 2.5×10^{-6} M; Compound : DNA ratio = 1:50.

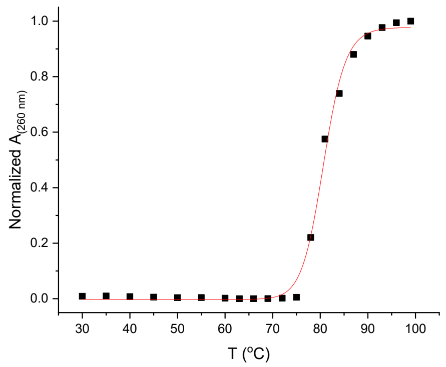

Free CT DNA

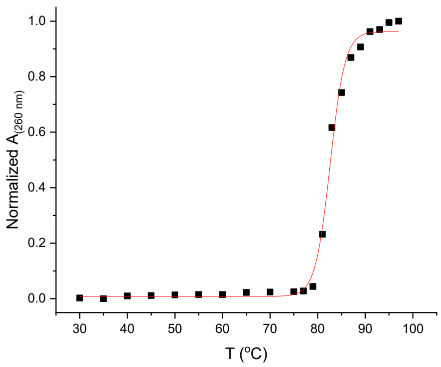

4′-Br-fenH

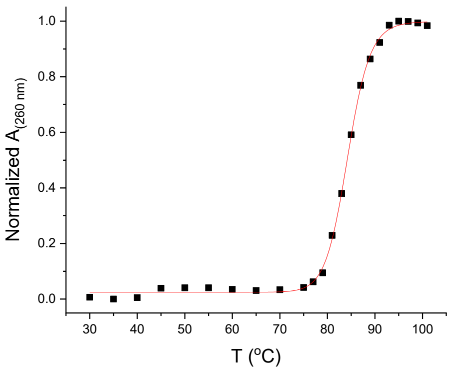

Complex **1**

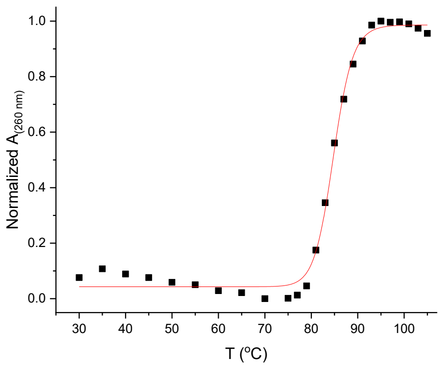

Complex **2**

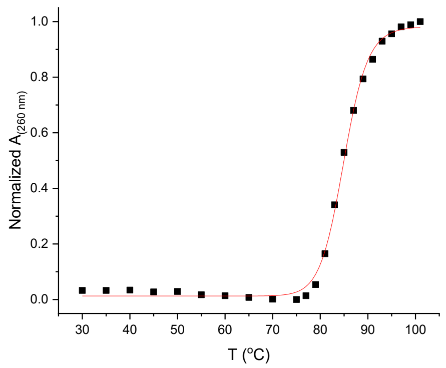

Complex **3**

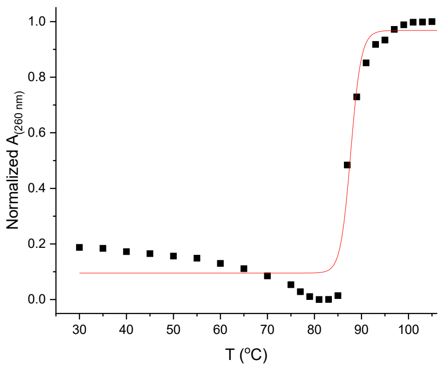

Complex **4**

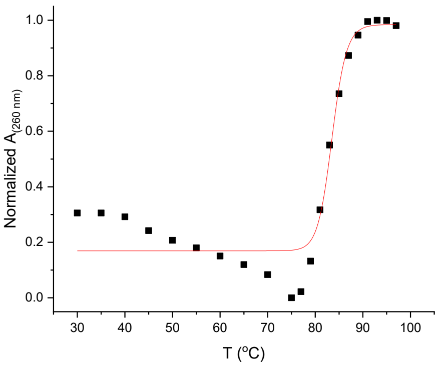

Complex **5**

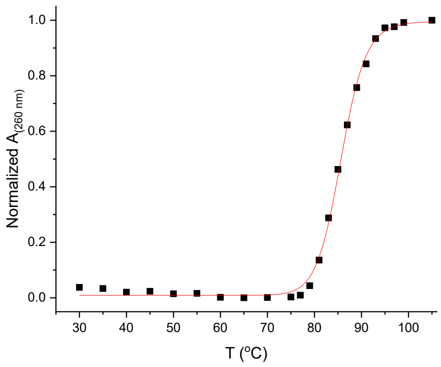

Complex **6**

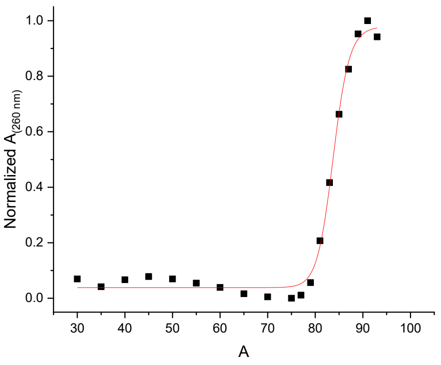

Complex **7**

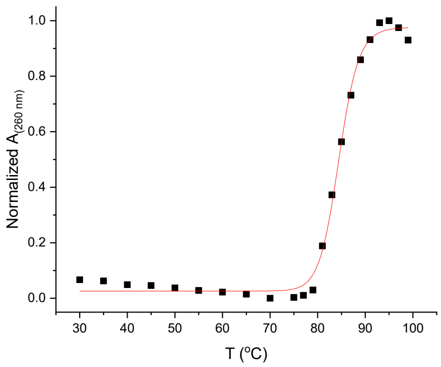

Complex **8**

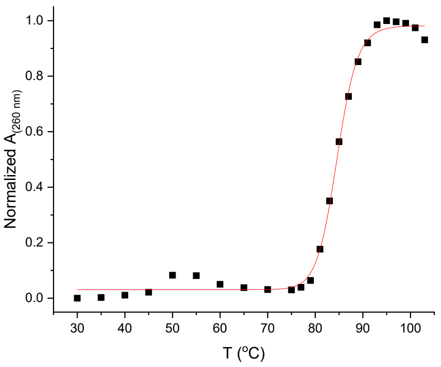

Complex **9**

Figure S13. van’t Hoff plots for the interaction of CT DNA with the compounds.

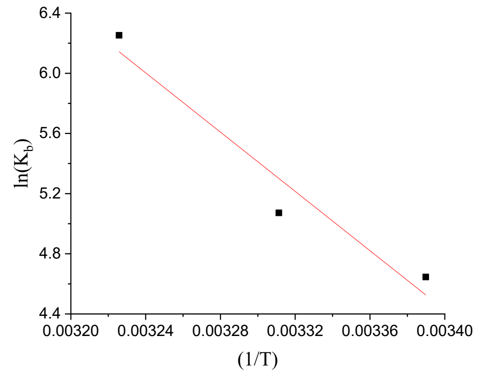

4′-Br-fenH

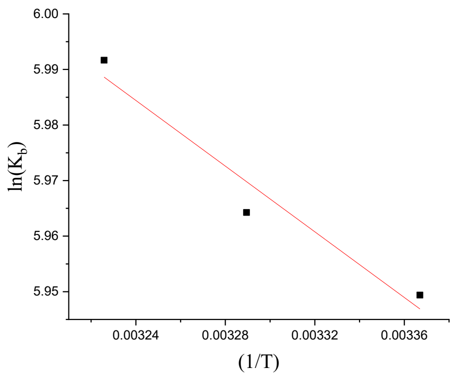

Complex **1**

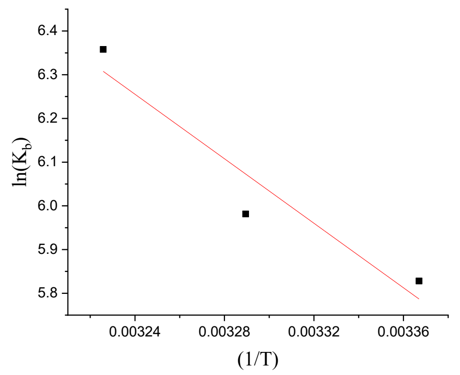

Complex **2**

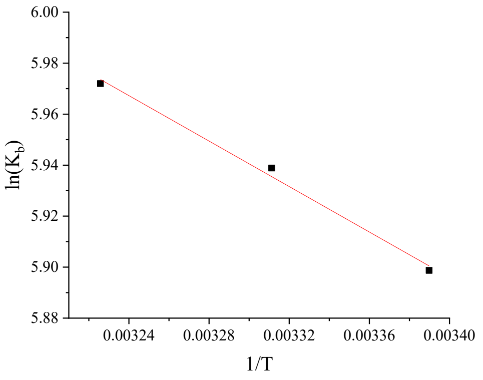

Complex **3**

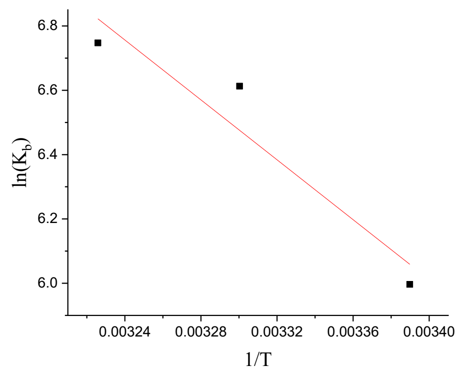

Complex **4**

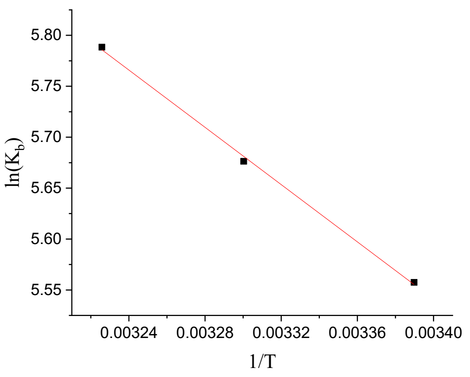

Complex **5**

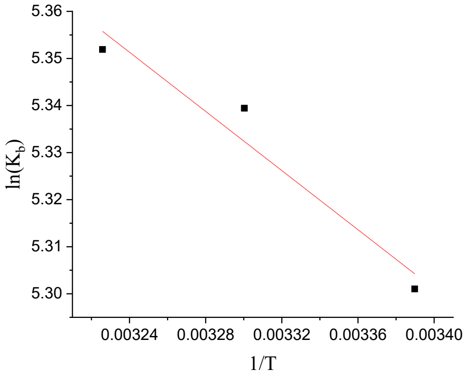

Complex **6**

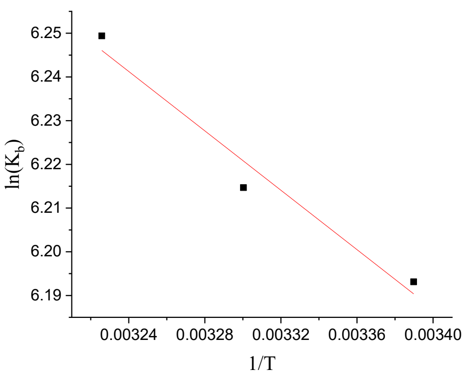

Complex **7**

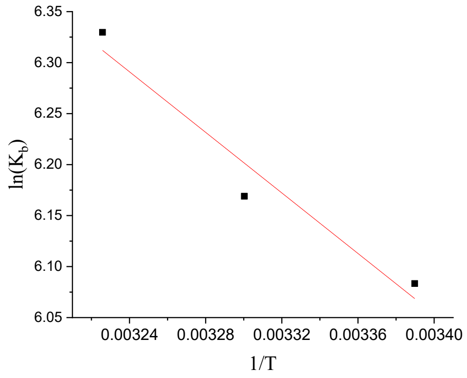

Complex **8**

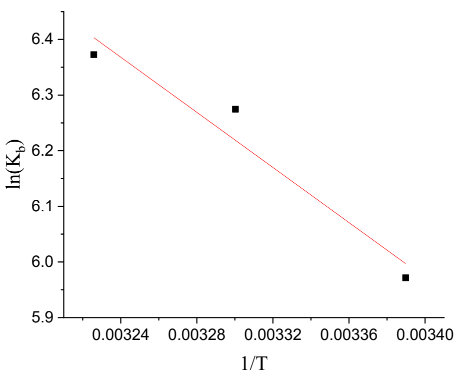

Complex **9**

Figure S14: Agarose gel electrophoretic pattern of EB-stained plasmid DNA (pBR322 plasmid DNA) after incubation with the compounds, in dark.
Conditions: [pDNA] = 50 μM/base pair. [compound] = 500 μM. Power supply: 65 V for 1 h.
Top: gel electrophoresis pictures: lane 1: pDNA; lane 2: HL + pDNA; lane 3: complex **1** + pDNA; lane 4: complex **2** + pDNA; lane 5: complex **3** + pDNA; lane 6: complex **4** + pDNA; lane 7: complex **5** + pDNA; lane 8: complex **6** + pDNA; lane 9: complex **7** + pDNA; lane 10: complex **8** + pDNA; lane 11: complex **9** + pDNA.
Bottom: calculation of the % conversion to ss and ds damage. DNA forms: form I = supercoiled pDNA, form II = relaxed pDNA, and form III = linear pDNA.

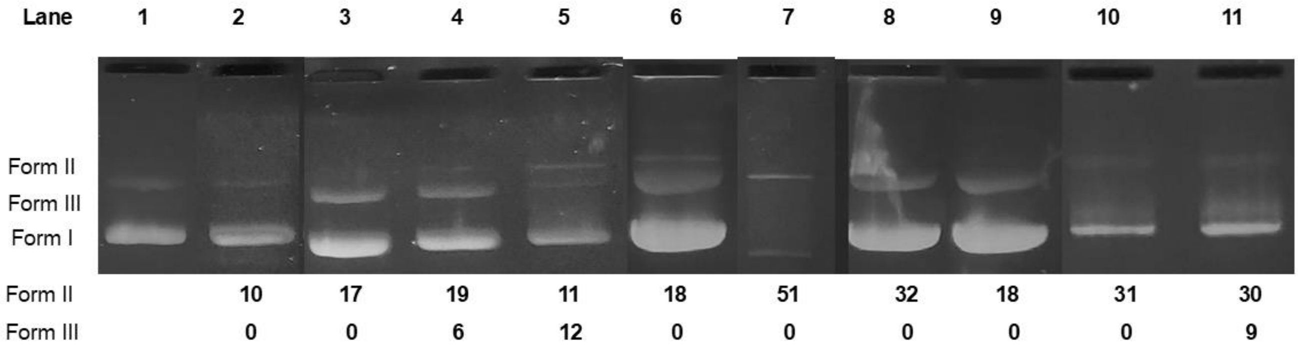

Figure S15: Agarose gel electrophoretic pattern of EB-stained plasmid DNA (pBR322 plasmid DNA) with the compounds, after irradiation at 312 nm (UV-B) for 30 min.
Conditions: [pDNA] = 50 μM/base pair; [compound] = 500 μM. Power supply: 65 V for 1 h.
Top: gel electrophoresis pictures: lane 1: pDNA + irradiation; lane 2: HL + pDNA+ irradiation; lane 3: complex **1** + pDNA+ irradiation; lane 4: complex **2** + pDNA+ irradiation; lane 5: complex **3** + pDNA+ irradiation; lane 6: complex **4** + pDNA+ irradiation; lane 7: complex **5** + pDNA+ irradiation; lane 8: complex **6** + pDNA+ irradiation; lane 9: complex **7** + pDNA+ irradiation; lane 10: complex **8** + pDNA+ irradiation; lane 11: complex **9** + pDNA+ irradiation.
Bottom: calculation of the % conversion to ss and ds damage. DNA forms: form I = supercoiled pDNA, form II = relaxed pDNA, and form III = linear pDNA.

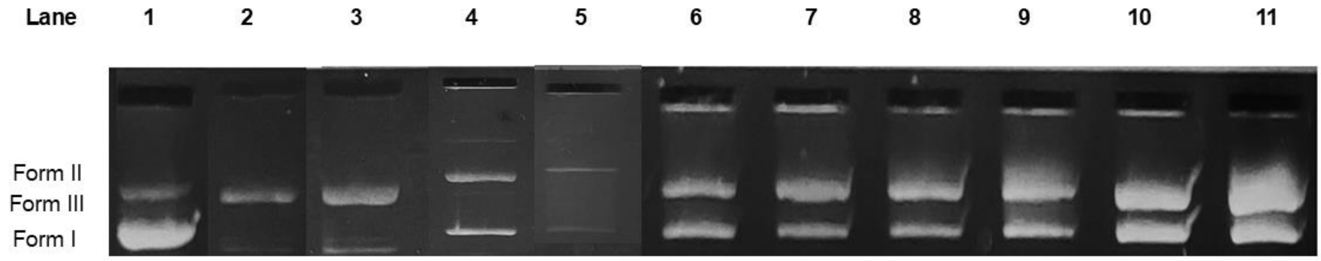

	1	2	3	4	5	6	7	8	9	10	11
Form II		70	61	25	61	38	43	52	51	46	56
Form III		0	0	0	0	0	0	0	0	0	0

Figure S16: Agarose gel electrophoretic pattern of EB-stained plasmid DNA (pBR322 plasmid DNA) with the compounds, after irradiation at 365 nm (UV-A) for 30 min.
Conditions: [pDNA] = 50 μM/base pair; [compound] = 500 μM. Power supply: 65 V for 1 h.
Top: gel electrophoresis pictures: lane 1: pDNA + irradiation; lane 2: HL + pDNA+ irradiation; lane 3: complex **1** + pDNA+ irradiation; lane 4: complex **2** + pDNA+ irradiation; lane 5: complex **3** + pDNA+ irradiation; lane 6: complex **4** + pDNA+ irradiation; lane 7: complex **5** + pDNA+ irradiation; lane 8: complex **6** + pDNA+ irradiation; lane 9: complex **7** + pDNA+ irradiation; lane 10: complex **8** + pDNA+ irradiation; lane 11: complex **9** + pDNA+ irradiation.
Bottom: calculation of the % conversion to ss and ds damage. DNA forms: form I = supercoiled pDNA, form II = relaxed pDNA, and form III = linear pDNA.

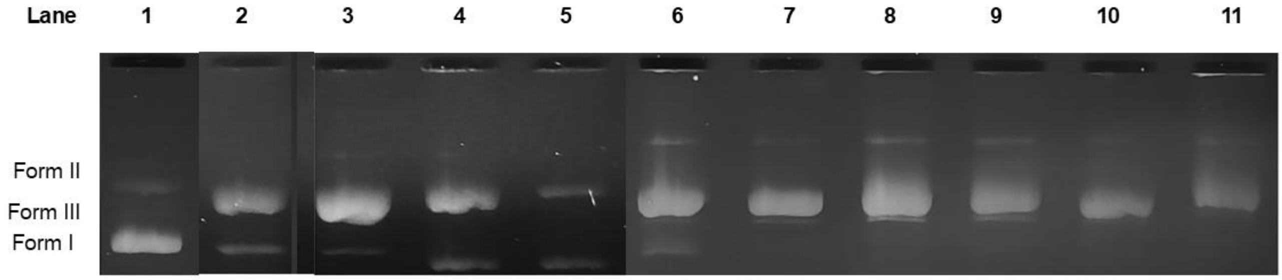

Lane	1	2	3	4	5	6	7	8	9	10	11
Form II		64	71	64	40	63	81	82	78	92	91
Form III		0	0	0	0	0	11	12	15	0	0

Figure S17: Agarose gel electrophoretic pattern of EB-stained plasmid DNA (pBR322 plasmid DNA) with the compounds, after irradiation under visible light for 2 h.
Conditions: [pDNA] = 50 μM/base pair; [compound] = 500 μM. Power supply: 65 V for 1 h.
Top: gel electrophoresis pictures: lane 1: pDNA + irradiation; lane 2: HL + pDNA+ irradiation; lane 3: complex **1** + pDNA+ irradiation; lane 4: complex **2** + pDNA+ irradiation; lane 5: complex **3** + pDNA+ irradiation; lane 6: complex **4** + pDNA+ irradiation; lane 7: complex **5** + pDNA+ irradiation; lane 8: complex **6** + pDNA+ irradiation; lane 9: complex **7** + pDNA+ irradiation; lane 10: complex **8** + pDNA+ irradiation; lane 11: complex **9** + pDNA+ irradiation.
Bottom: calculation of the % conversion to ss and ds damage. DNA forms: form I = supercoiled pDNA, form II = relaxed pDNA, and form III = linear pDNA.

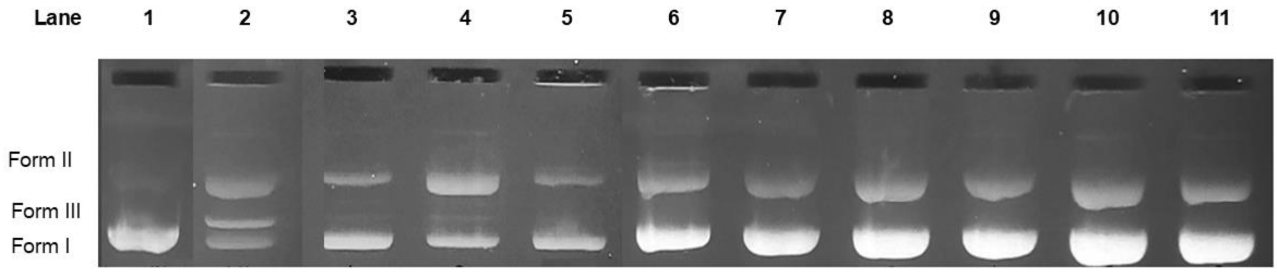

	1	2	3	4	5	6	7	8	9	10	11
Form II		37	34	46	26	37	24	29	15	26	23
Form III		22	0	9	0	0	0	0	0	0	0

Figure S18. Fluorescence emission spectra of BSA in buffer solution in the presence of increasing amounts of the compounds.
Conditions: $\lambda_{excitation}$ = 295 nm. [BSA] = 3 μM. Buffer solution: 150 mM NaCl and 15 mM trisodium citrate at pH 7.0.
The arrow shows the changes of intensity upon increasing amounts of the compound.

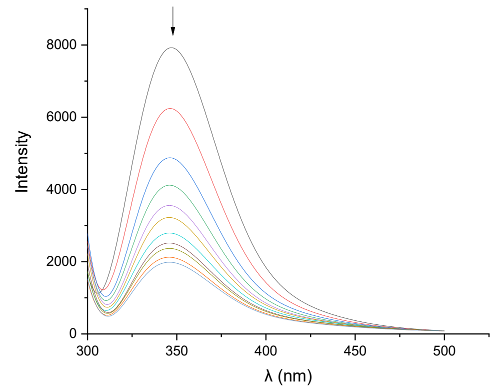

4′-Br-fenH

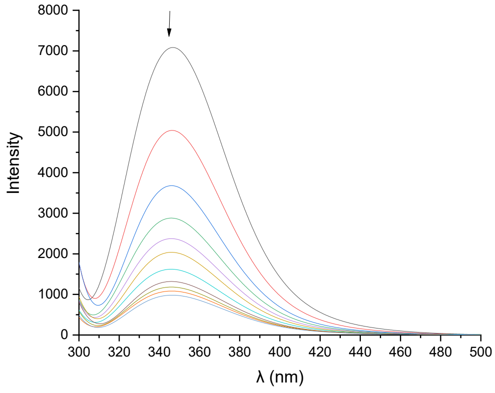

Complex **1**

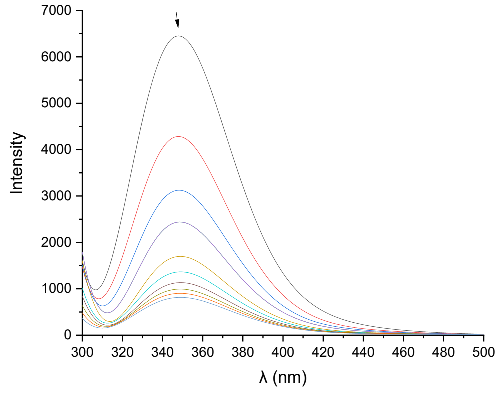

Complex **2**

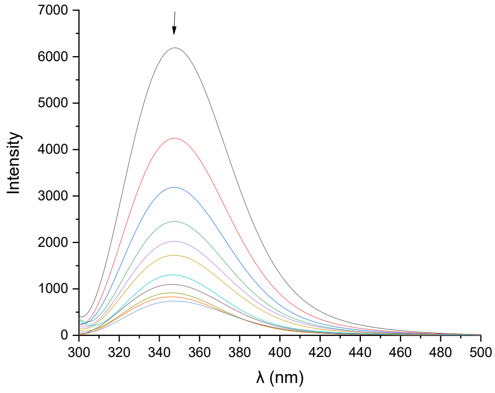

Complex **3**

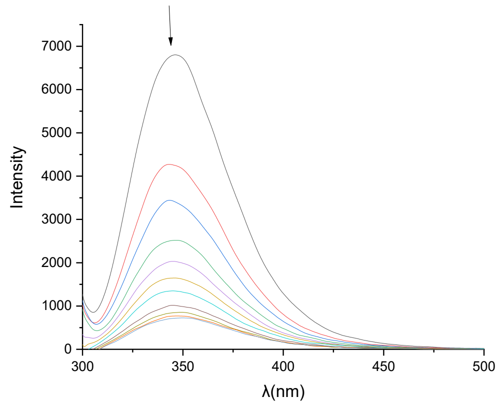

Complex **4**

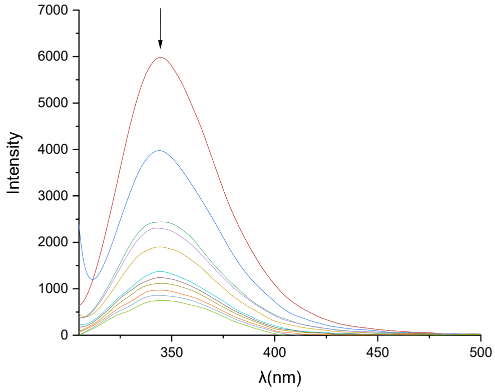

Complex **5**

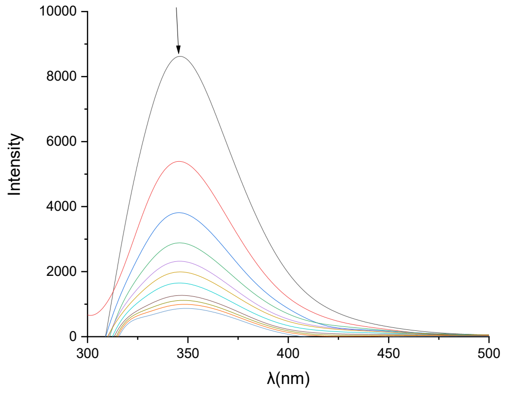

Complex **6**

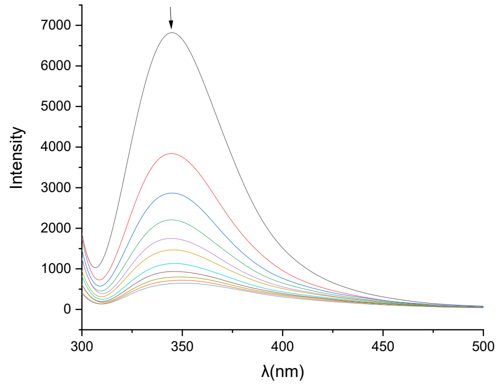

Complex **7**

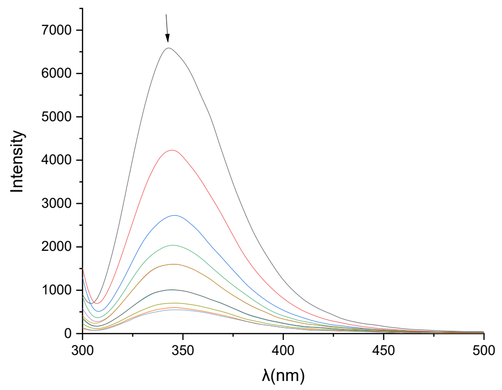

Complex **8**

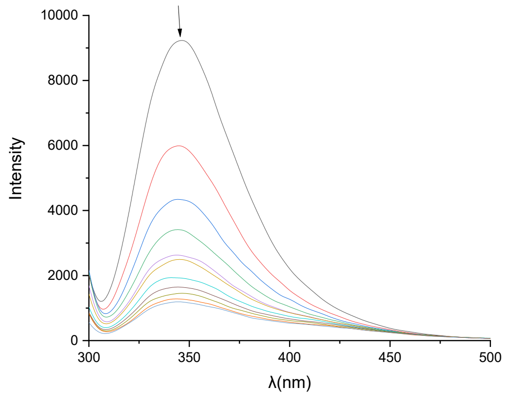

Complex **9**

Figure S19. Fluorescence emission spectra of HSA in buffer solution in the presence of increasing amounts of the compounds.
Conditions: $\lambda_{excitation}$= 295 nm. [HSA] = 3 μM. Buffer solution: 150 mM NaCl and 15 mM trisodium citrate at pH 7.0.
The arrow shows the changes of intensity upon increasing amounts of the compound.

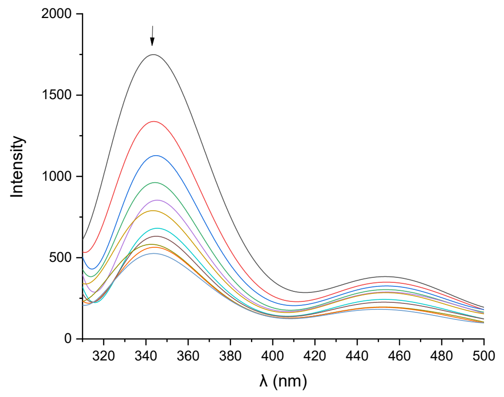

4′-Br-fenH

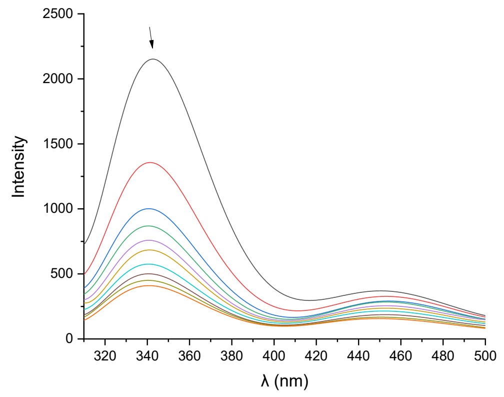

Complex **1**

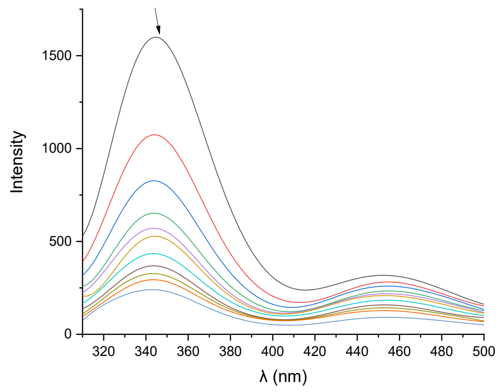

Complex **2**

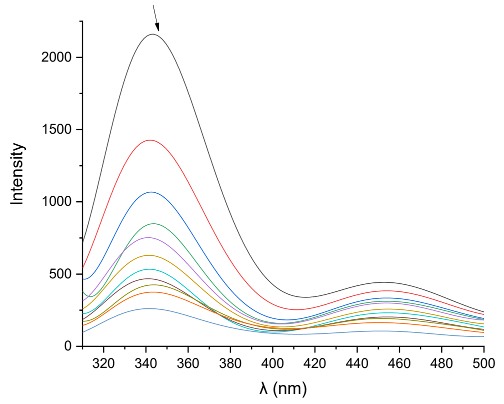

Complex **3**

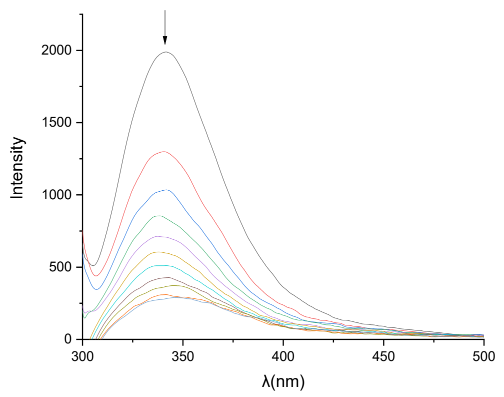

Complex **4**

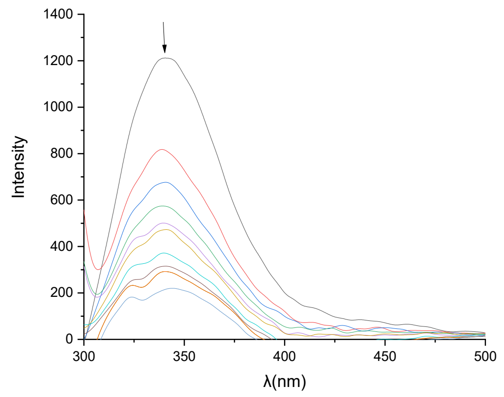

Complex **5**

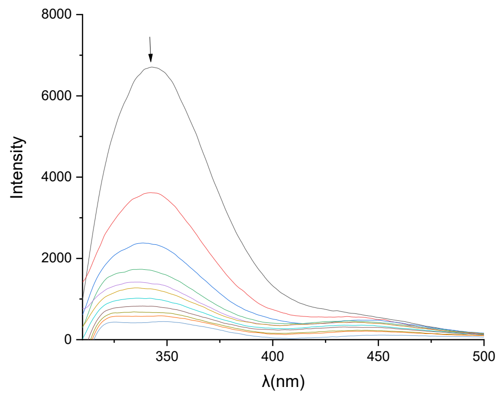

Complex **6**

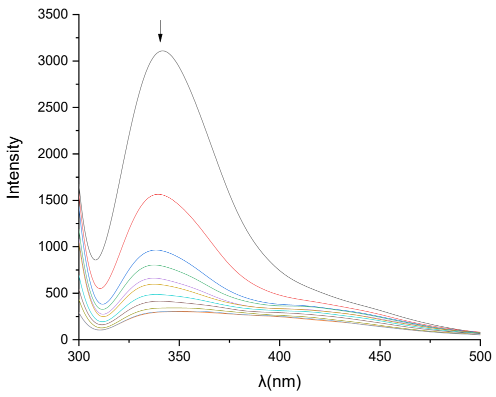

Complex **7**

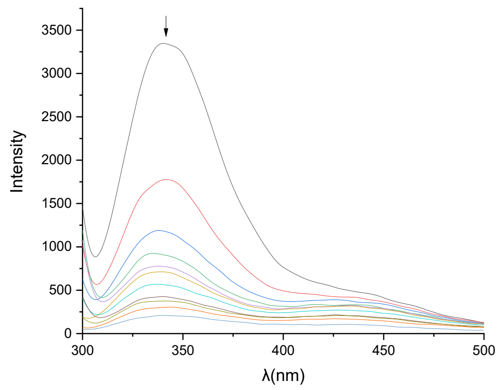

Complex **8**

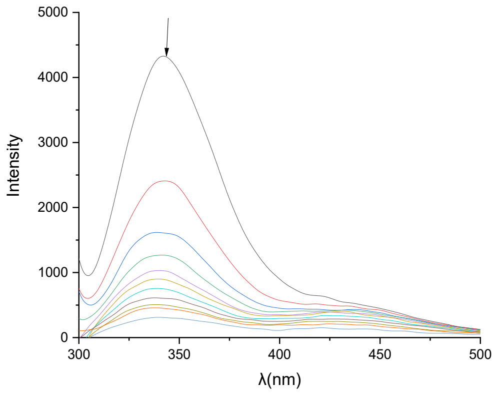

Complex **9**

Figure S20. Stern-Volmer plots of the BSA-quenching experiments upon addition of the compounds.

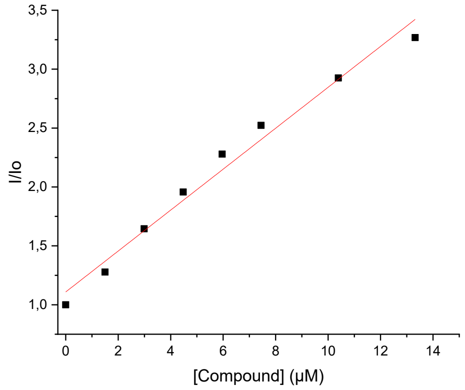

4′-Br-fenH

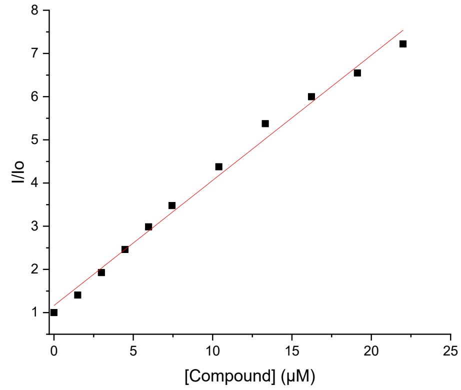

Complex **1**

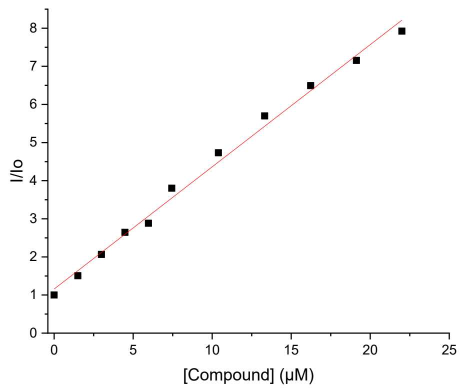

Complex **2**

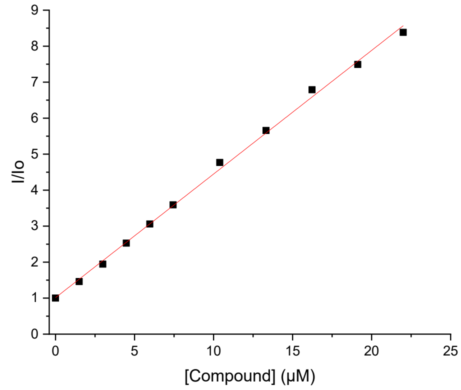

Complex **3**

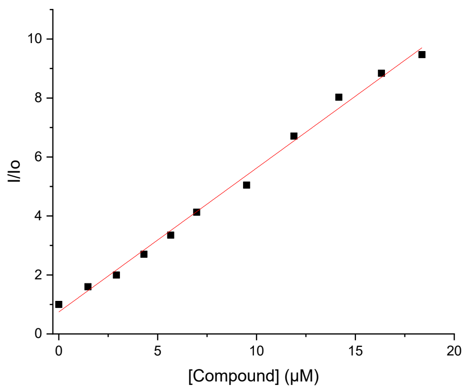

Complex **4**

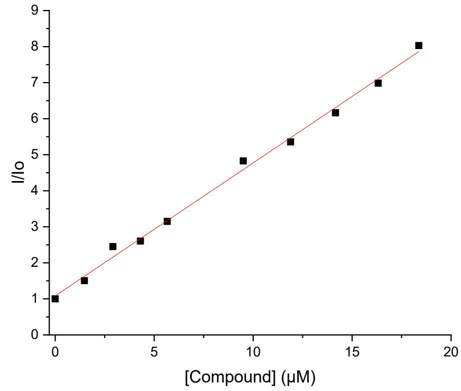

Complex **5**

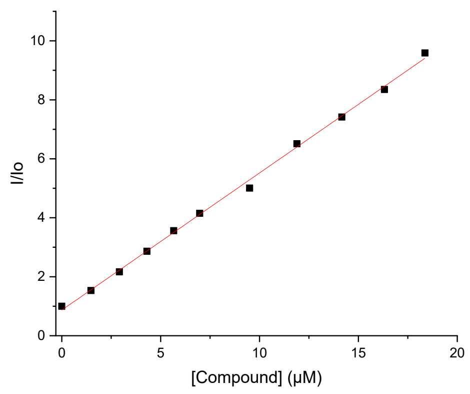

Complex **6**

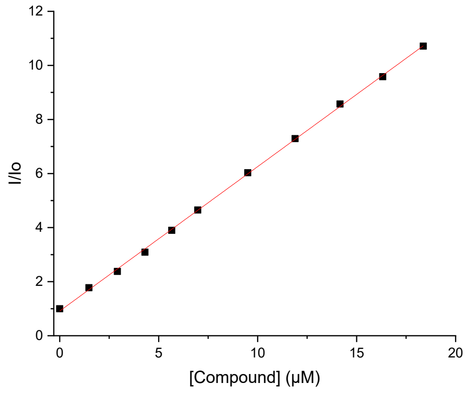

Complex **7**

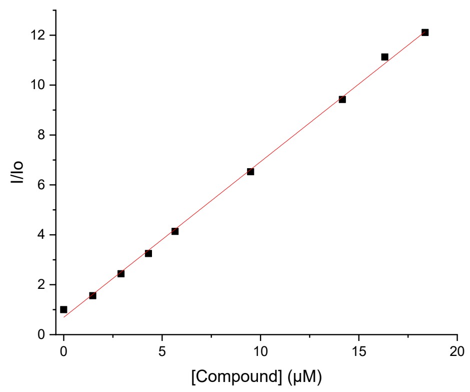

Complex **8**

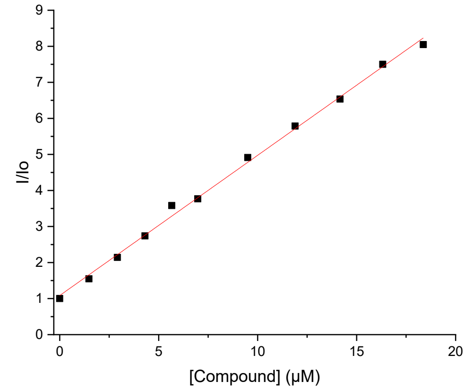

Complex **9**

Figure S21. Stern-Volmer plots of the HSA-quenching experiments upon addition of the compounds.

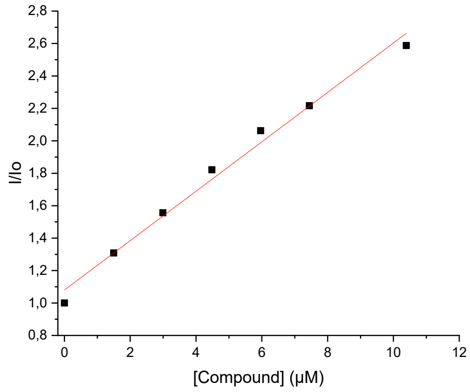

4′-Br-fenH

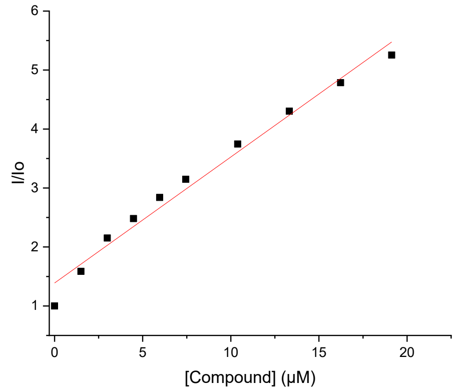

Complex **1**

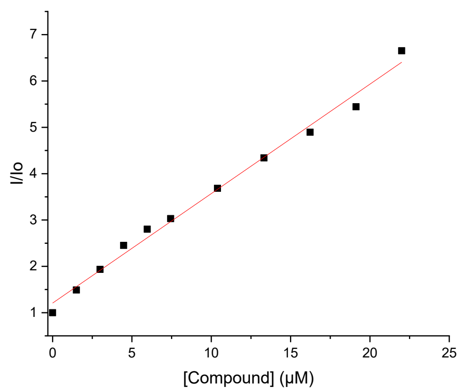

Complex **2**

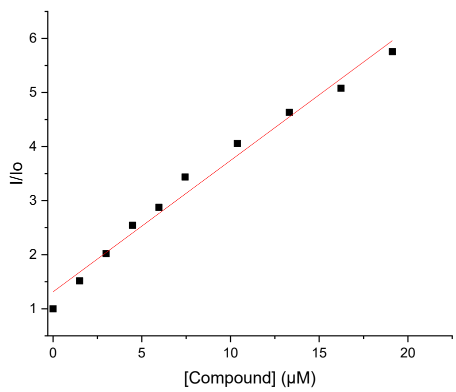

Complex **3**

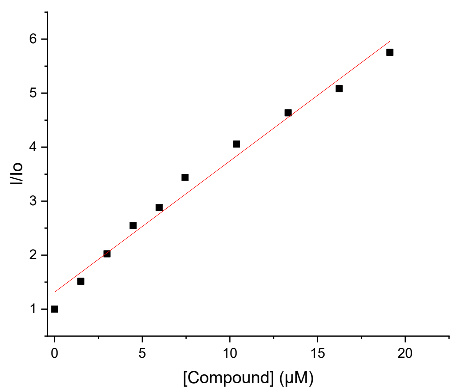

Complex **4**

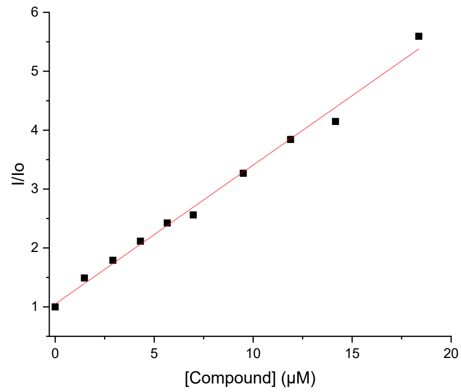

Complex **5**

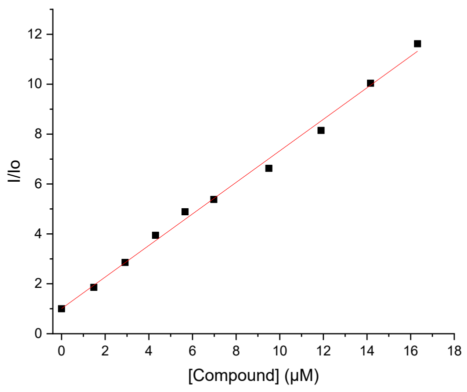

Complex **6**

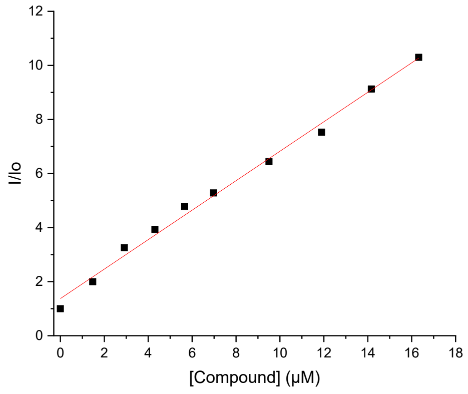

Complex **7**

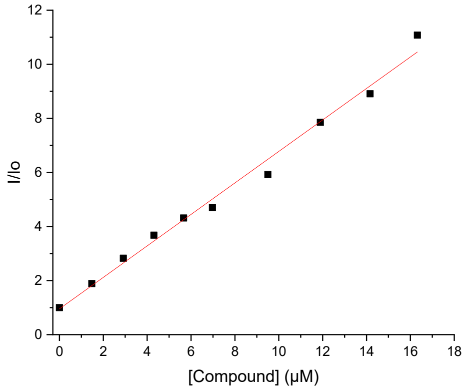

Complex **8**

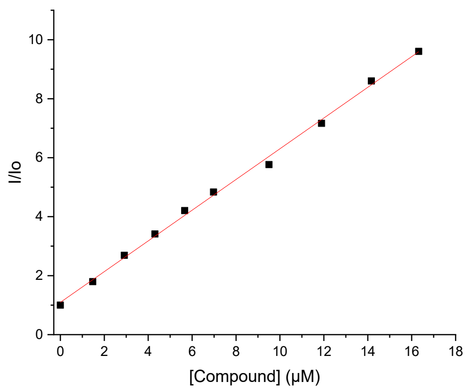

Complex **9**

Figure S22. Scatchard plots of the BSA-quenching experiments upon addition of the compounds.

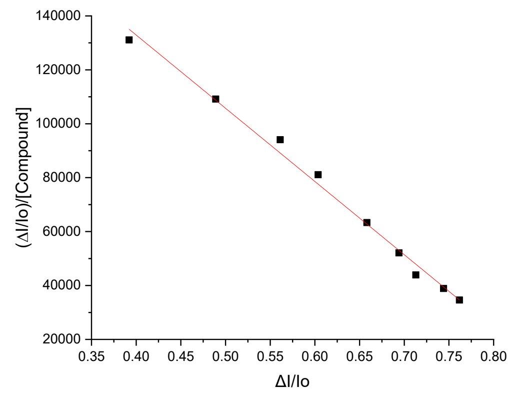

4′-Br-fenH

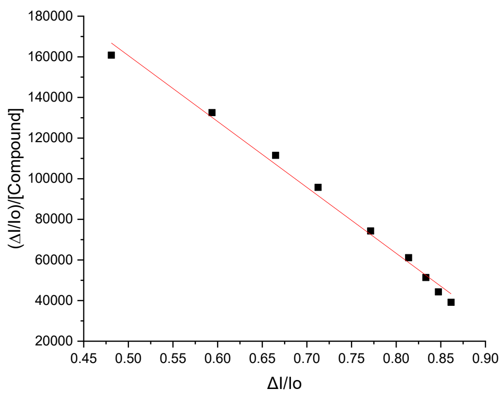

Complex **1**

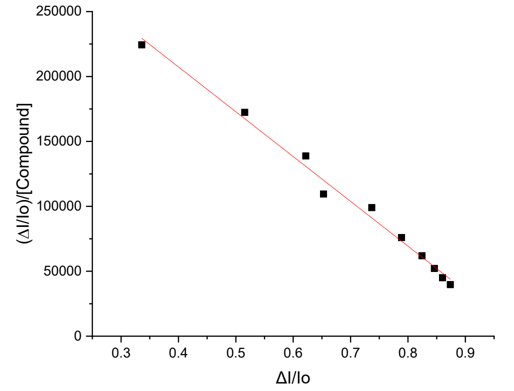

Complex **2**

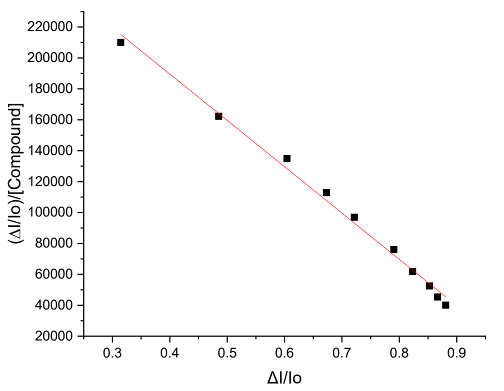

Complex **3**

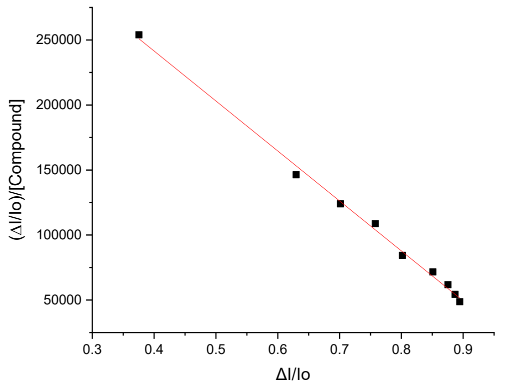

Complex **4**

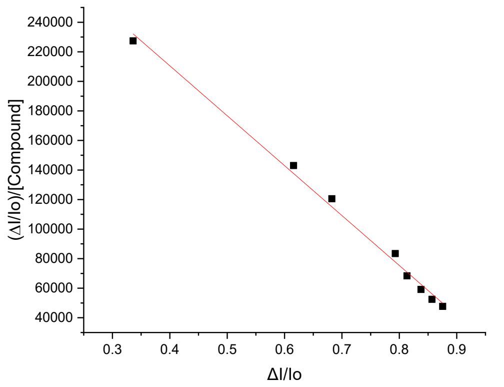

Complex **5**

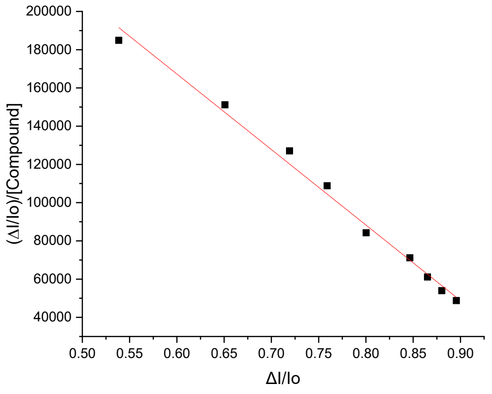

Complex **6**

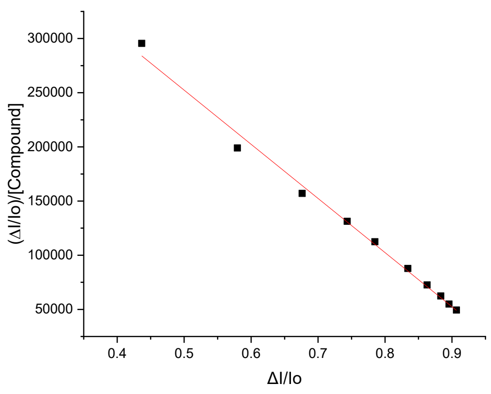

Complex **7**

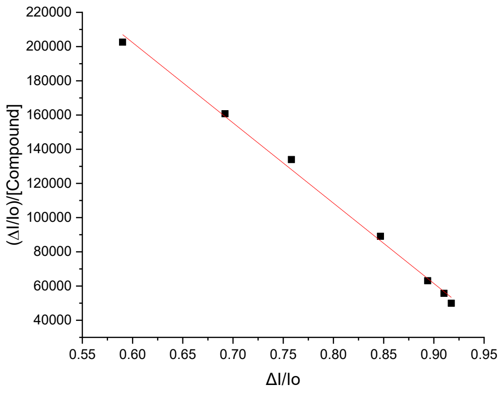

Complex **8**

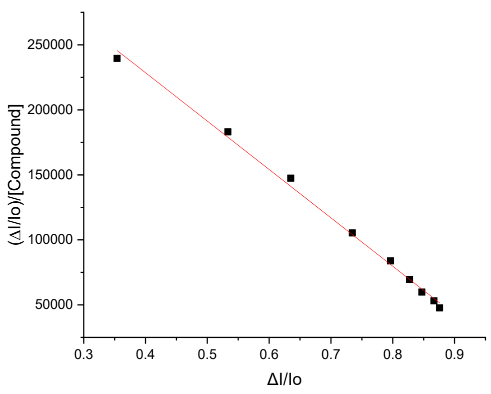

Complex **9**

Figure S23. Scatchard plots of the HSA-quenching experiments upon addition of the compounds.

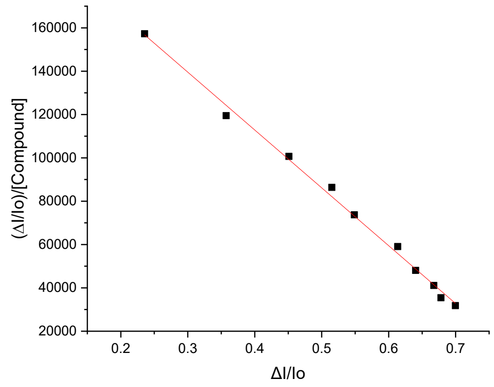

4′-Br-fenH

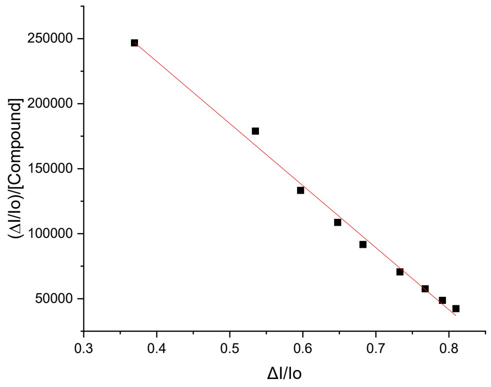

Complex **1**

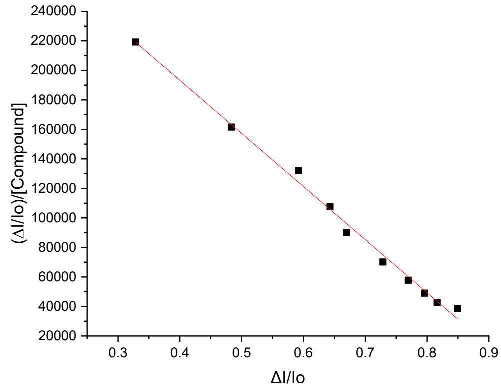

Complex **2**

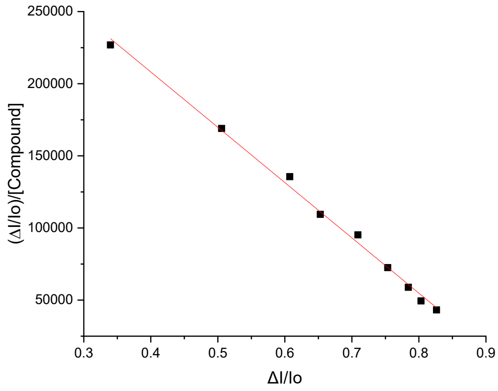

Complex **3**

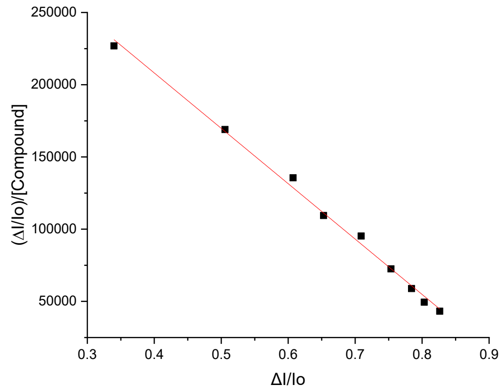

Complex **4**

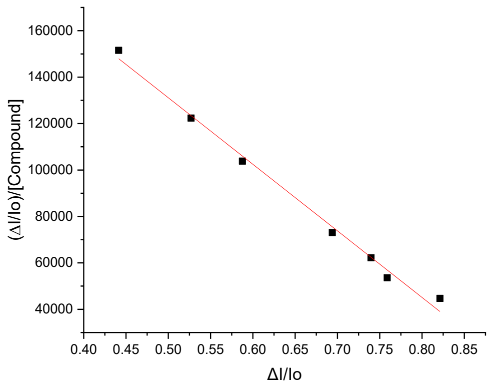

Complex **5**

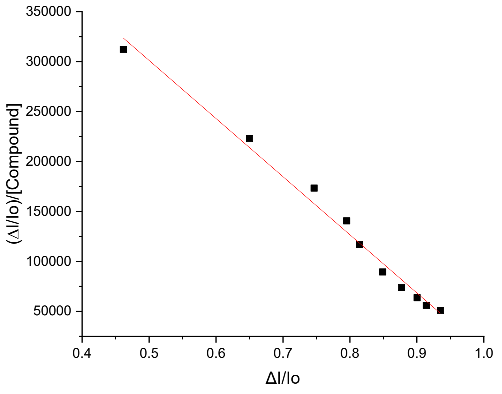

Complex **6**

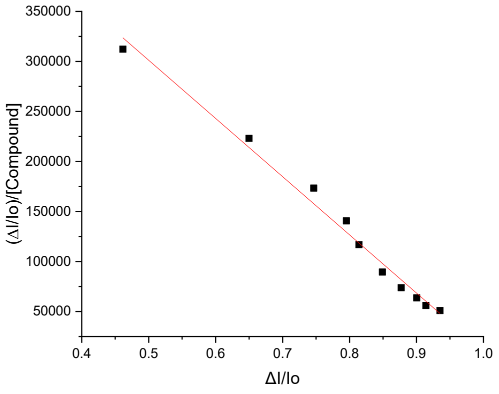

Complex **7**

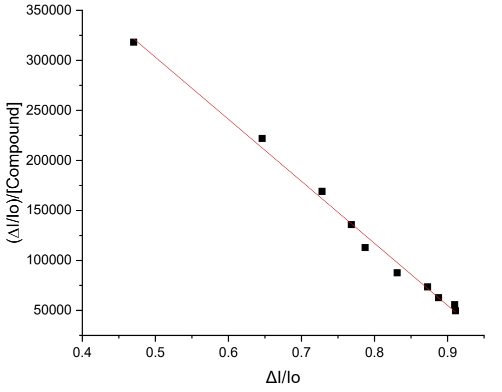

Complex **8**

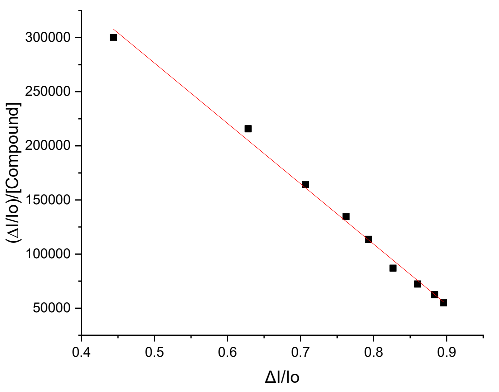

Complex **9**

Figure S24. Fluorescence emission spectra of BSA in buffer solution in the presence of warfarin upon addition of increasing amounts of the compounds.
Conditions: $\lambda_{excitation}$= 295 nm. [BSA] = 3 μM. Buffer solution: 150 mM NaCl and 15 mM trisodium citrate at pH 7.0. [Warfarin] = 3 μM.
The arrow shows the changes of intensity upon increasing amounts of the compound.

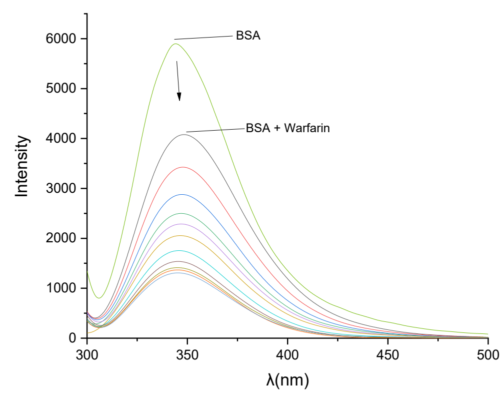

4′-Br-fenH

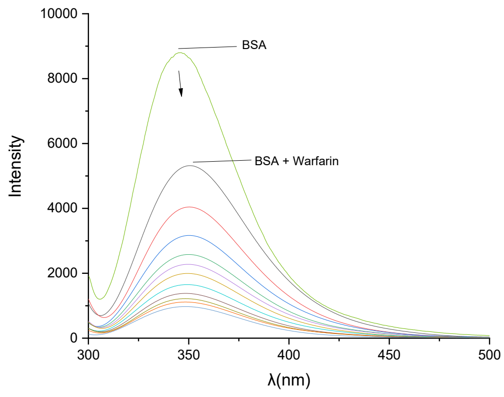

Complex **1**

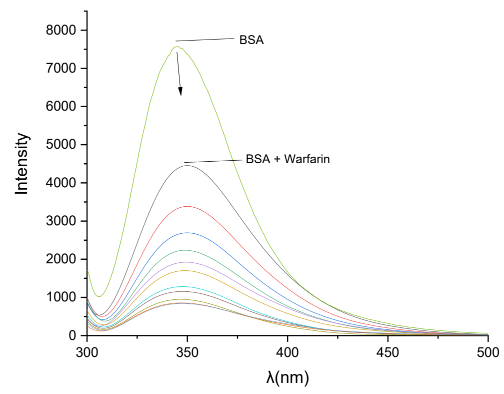

Complex **2**

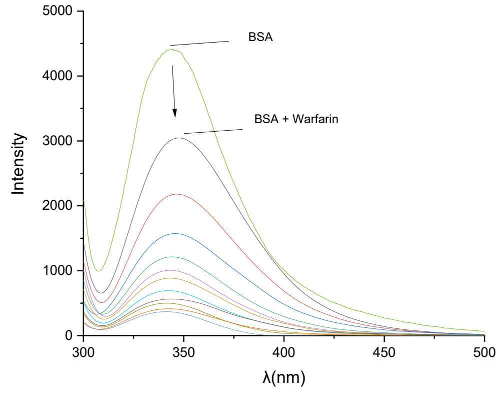

Complex **3**

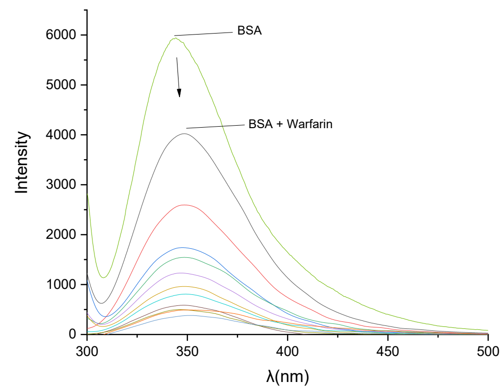

Complex **4**

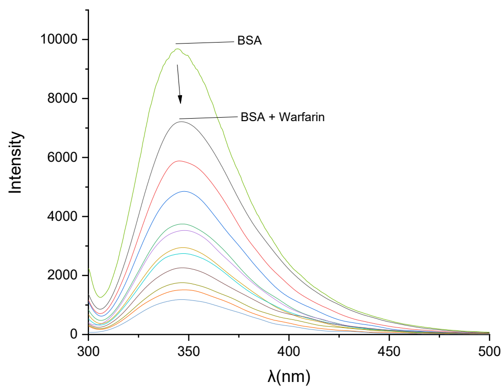

Complex **5**

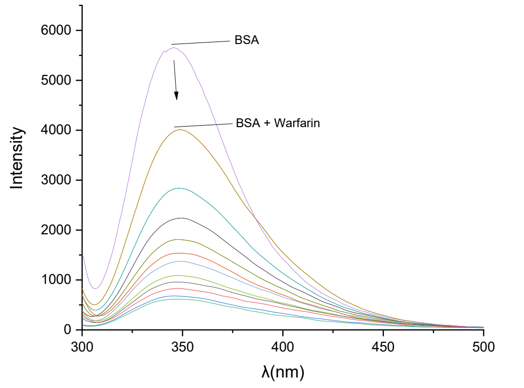

Complex **6**

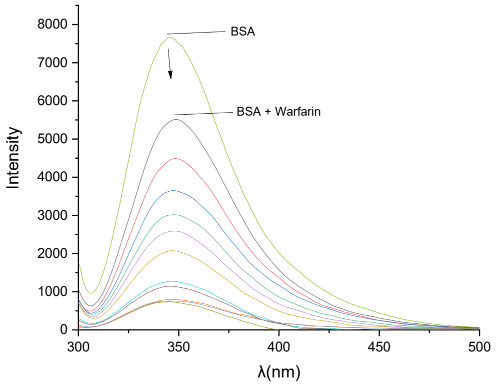

Complex **7**

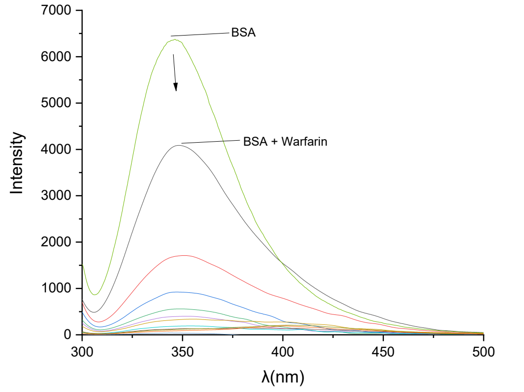

Complex **8**

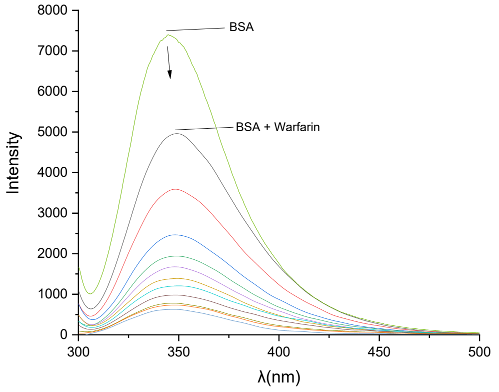

Complex **9**

Figure S25. Fluorescence emission spectra of BSA in buffer solution in the presence of ibuprofen upon addition of increasing amounts of the compounds.
Conditions: $\lambda_{excitation}$ = 295 nm. [BSA] = 3 μM. Buffer solution: 150 mM NaCl and 15 mM trisodium citrate at pH 7.0. [Ibuprofen] = 3 μM.
The arrow shows the changes of intensity upon increasing amounts of the compound.

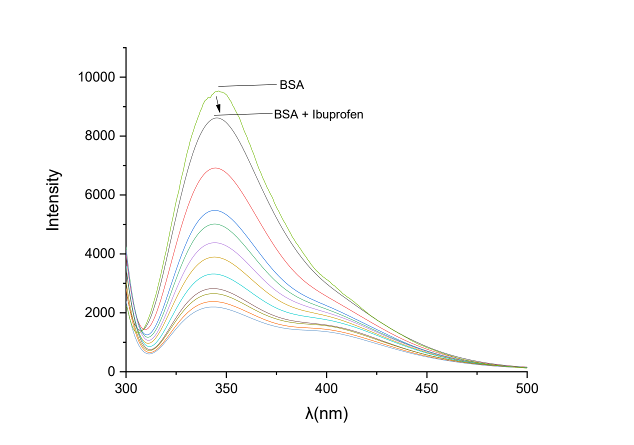

4′-Br-fenH

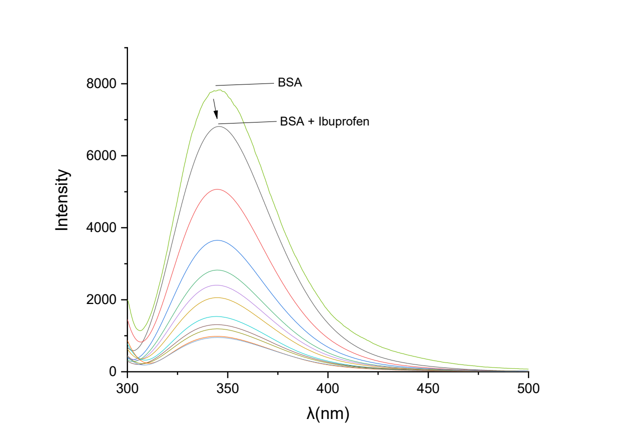

Complex **1**

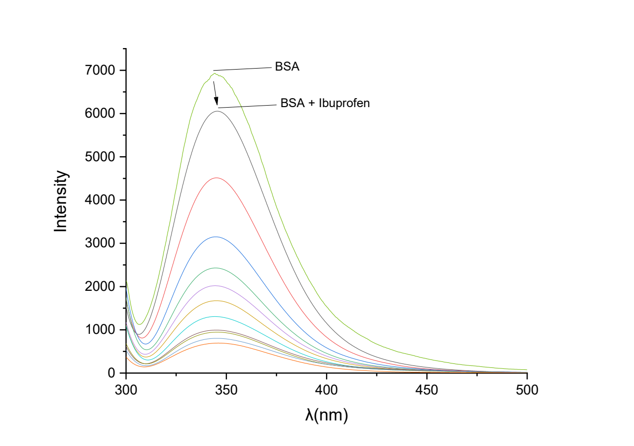

Complex **2**

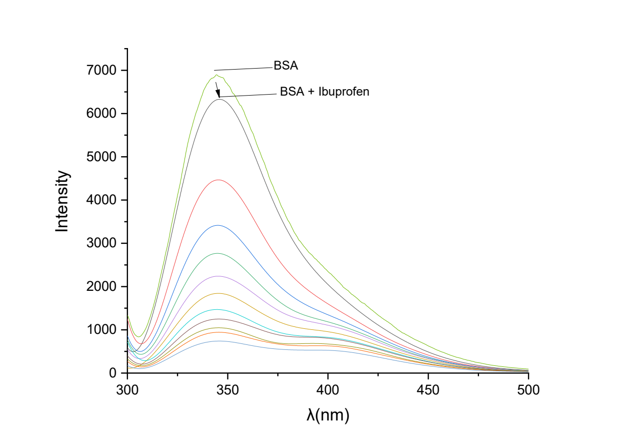

Complex **3**

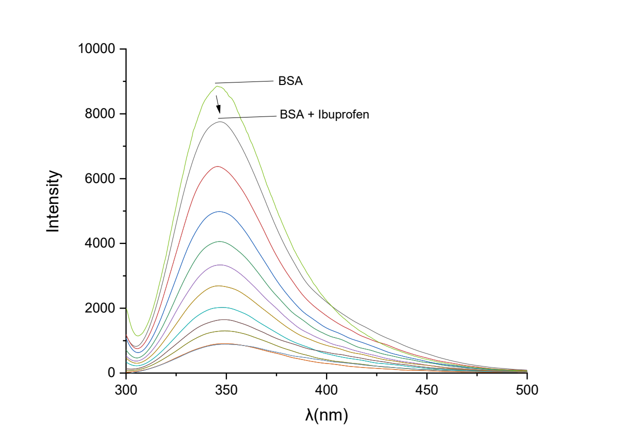

Complex **4**

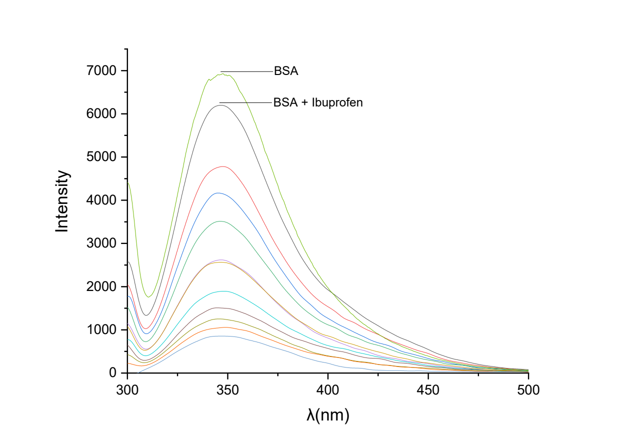

Complex **5**

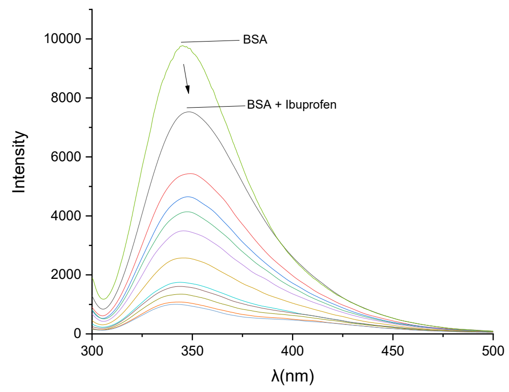

Complex **6**

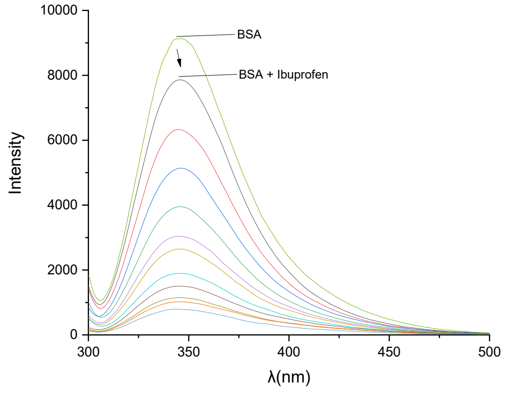

Complex **7**

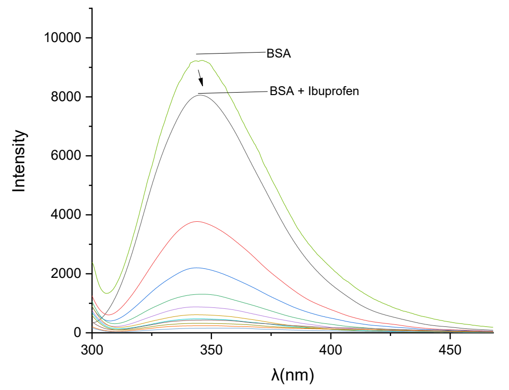

Complex **8**

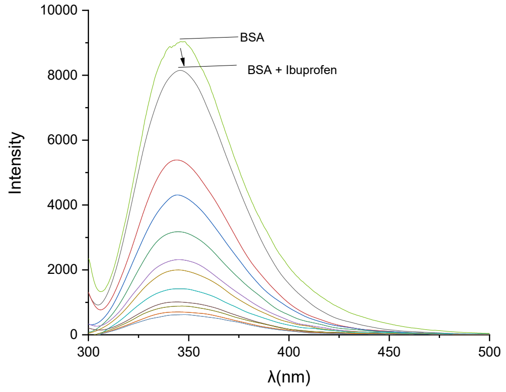

Complex **9**

Figure S26. Fluorescence emission spectra of HSA in buffer solution in the presence of warfarin upon addition of increasing amounts of the compounds.
Conditions: $\lambda_{excitation}$ = 295 nm. [HSA] = 3 μM. Buffer solution: 150 mM NaCl and 15 mM trisodium citrate at pH 7.0. [Warfarin] = 3 μM.
The arrow shows the changes of intensity upon increasing amounts of the compound.

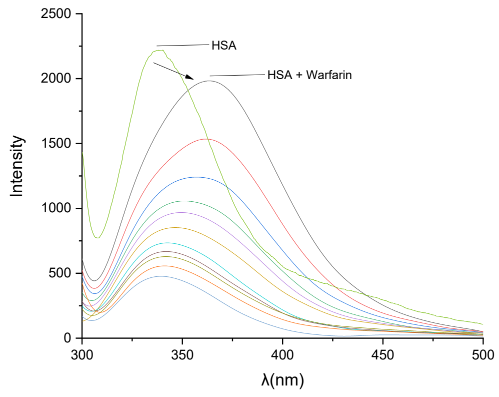

4′-Br-fenH

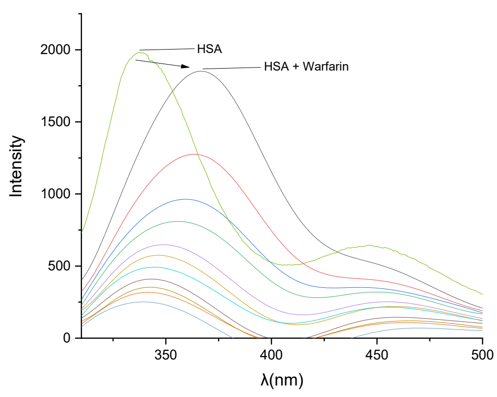

Complex **1**

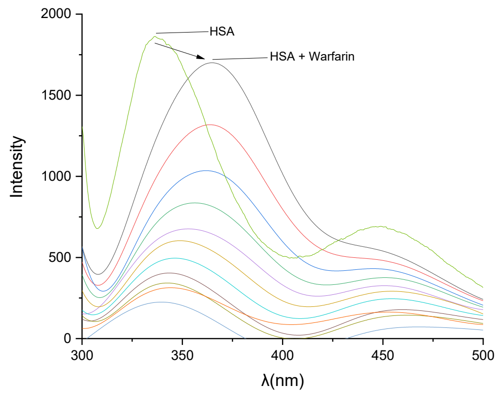

Complex **2**

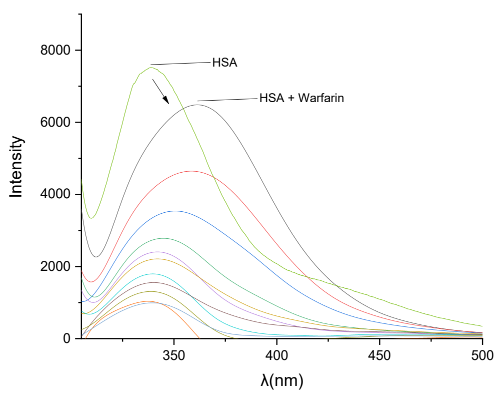

Complex **3**

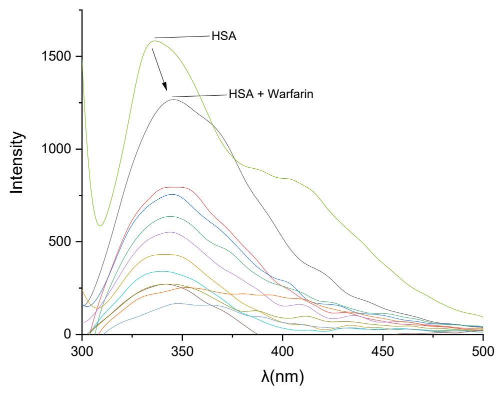

Complex **4**

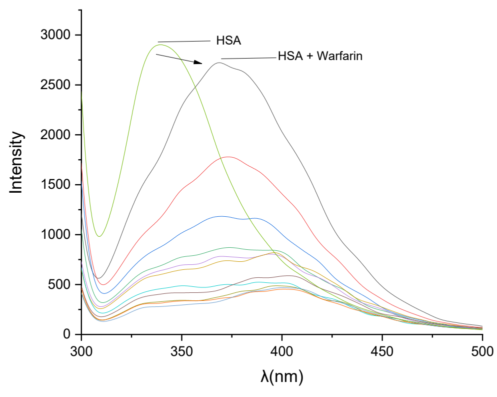

Complex **5**

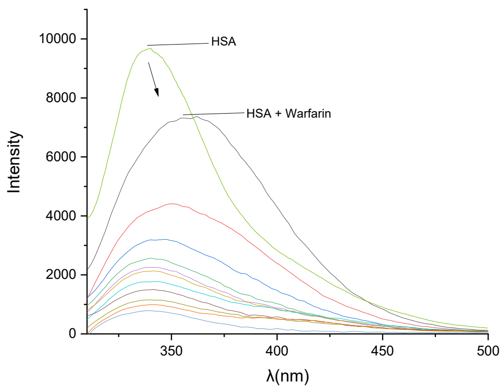

Complex **6**

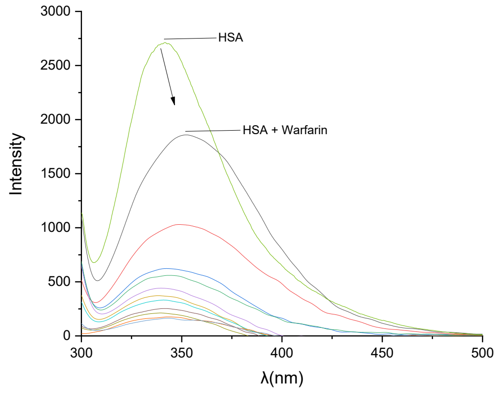

Complex **7**

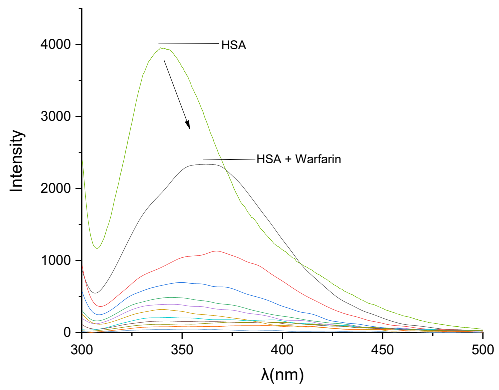

Complex **8**

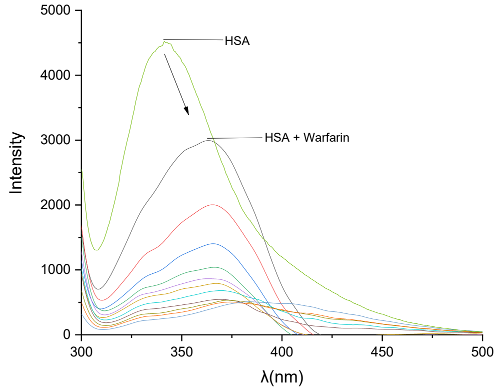

Complex **9**

Figure S27. Fluorescence emission spectra of HSA in buffer solution in the presence of ibuprofen upon addition of increasing amounts of the compounds.
Conditions: $\lambda_{excitation}$ = 295 nm. [HSA] = 3 μM. Buffer solution: 150 mM NaCl and 15 mM trisodium citrate at pH 7.0. [Ibuprofen] = 3 μM.
The arrow shows the changes of intensity upon increasing amounts of the compound.

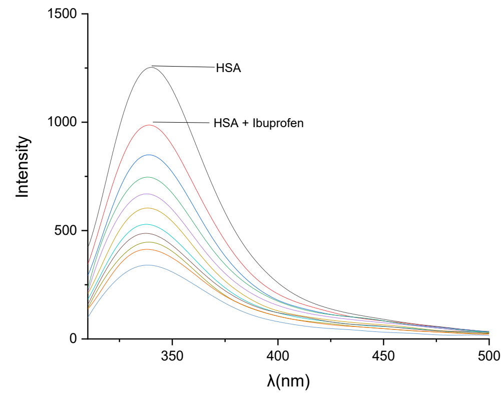

4′-Br-fenH

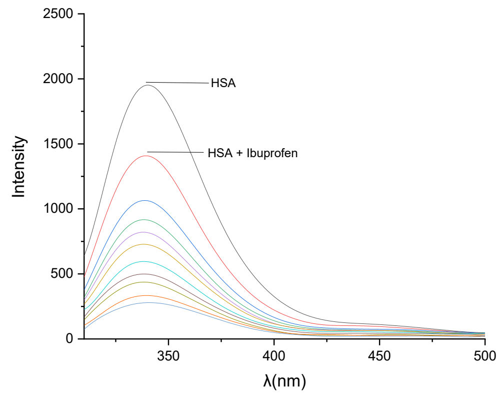

Complex **1**

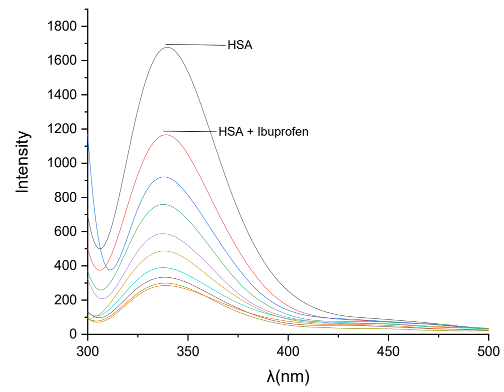

Complex **2**

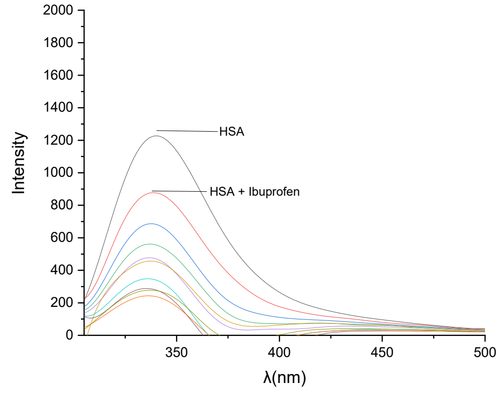

Complex **3**

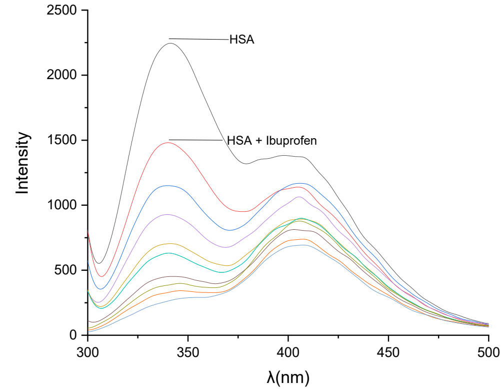

Complex **4**

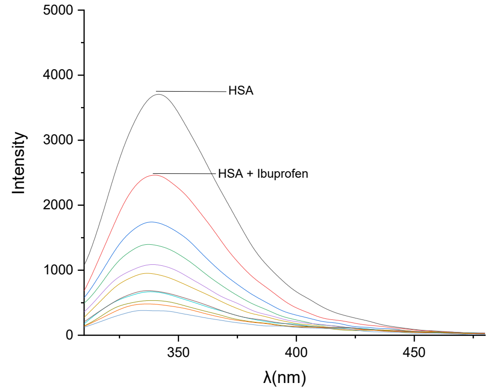

Complex **5**

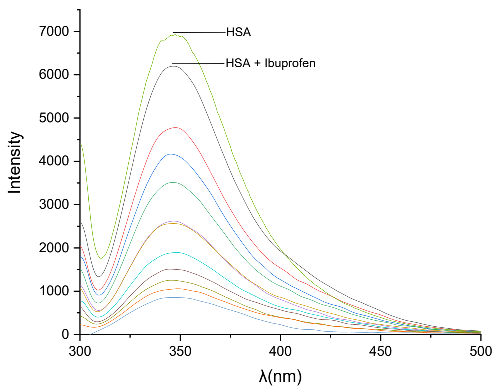

Complex **6**

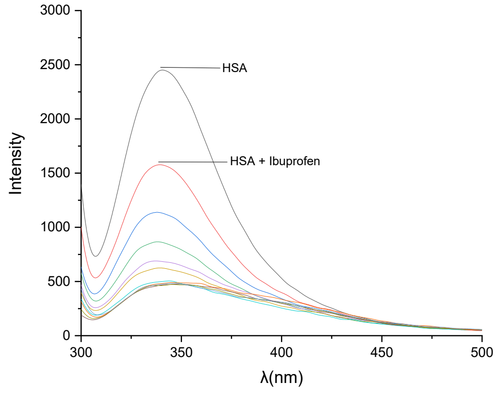

Complex **7**

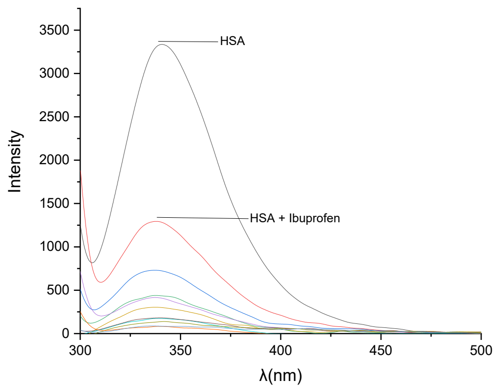

Complex **8**

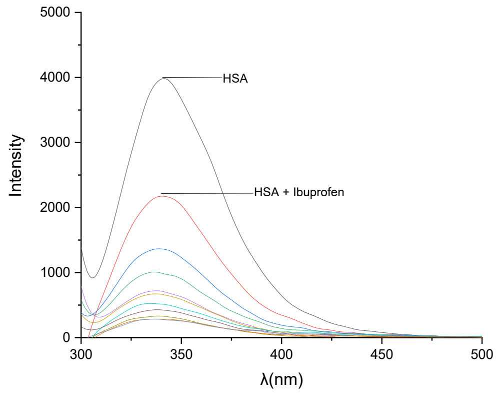

Complex **9**

Figure S28. Scatchard plots of the BSA quenching experiments in the presence of warfarin upon addition of the compounds.

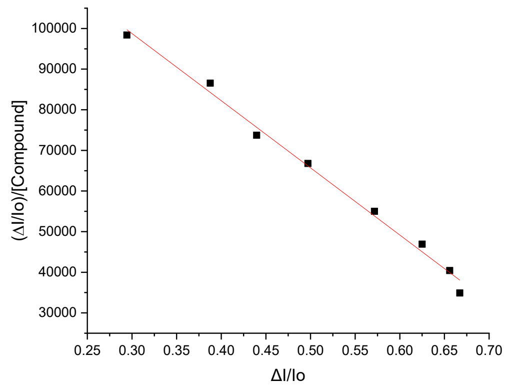

4′-Br-fenH

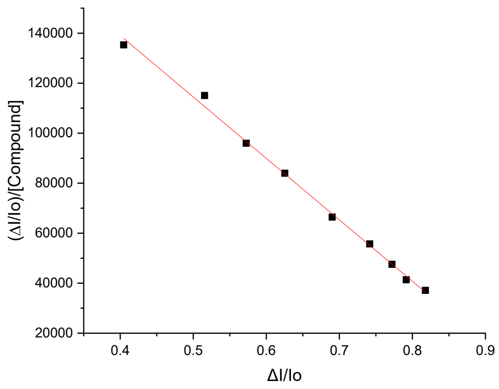

Complex **1**

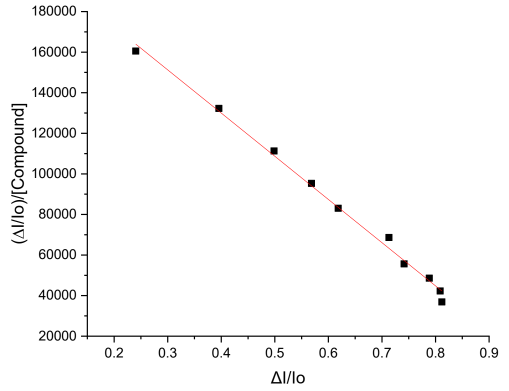

Complex **2**

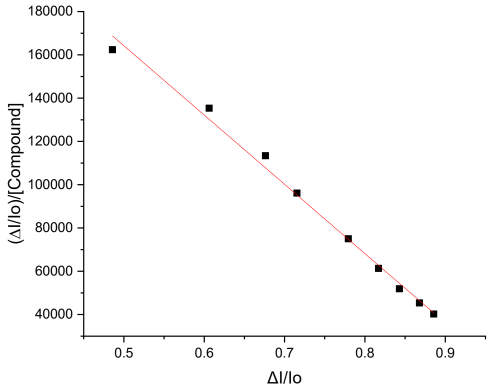

Complex **3**

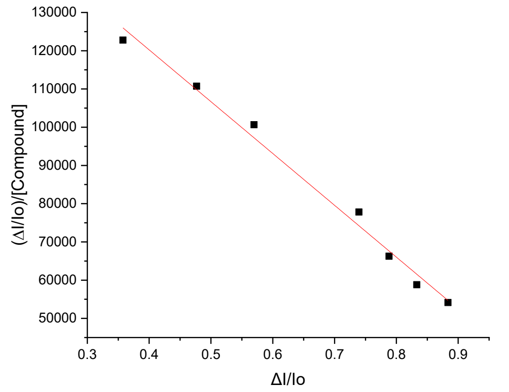

Complex **4**

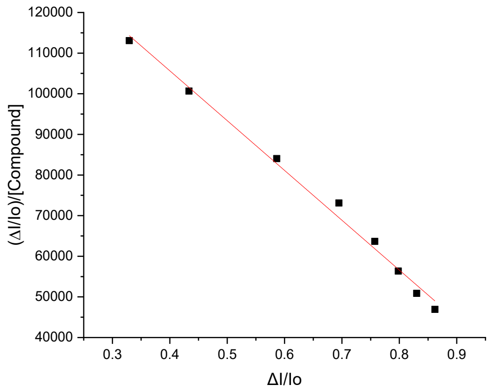

Complex **5**

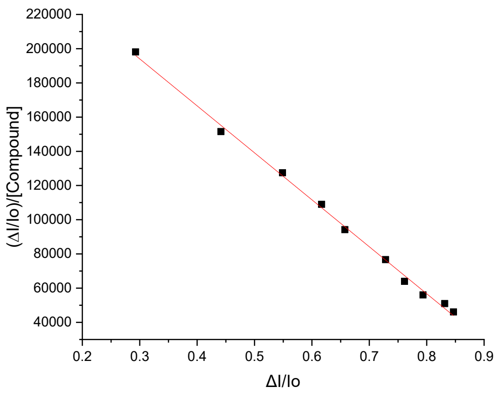

Complex **6**

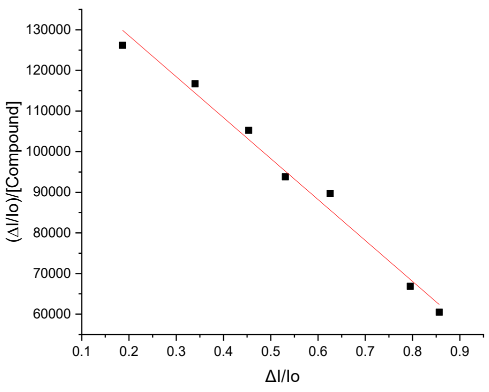

Complex **7**

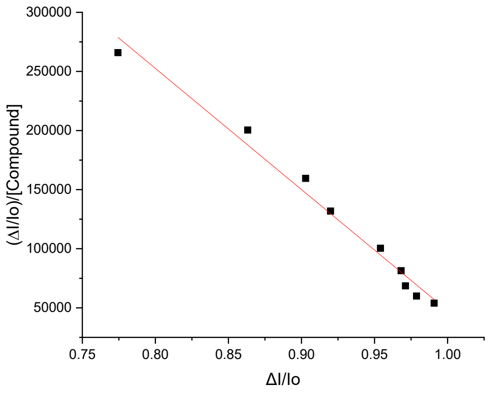

Complex **8**

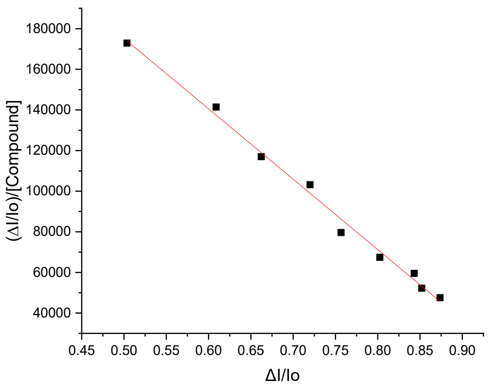

Complex **9**

Figure S29. Scatchard plots of the BSA quenching experiments in the presence of ibuprofen upon addition of the compounds.

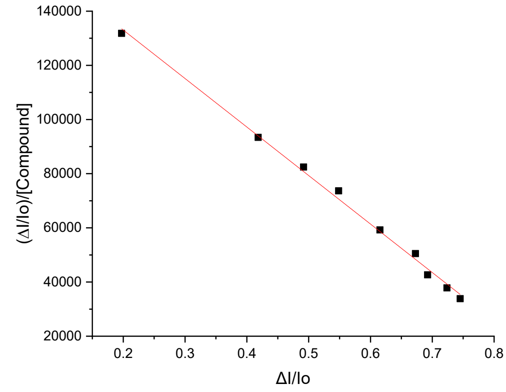

4′-Br-fenH

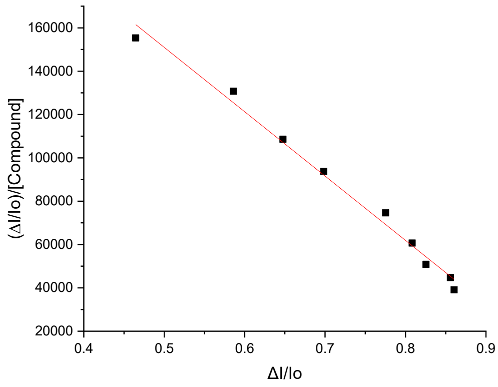

Complex **1**

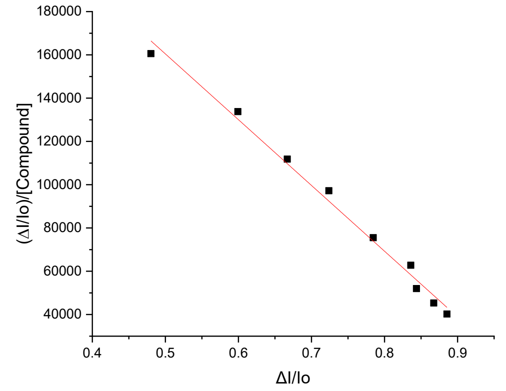

Complex **2**

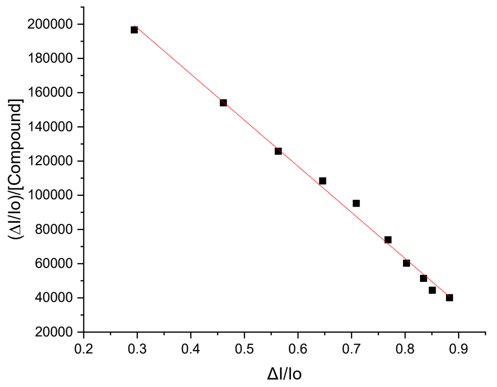

Complex **3**

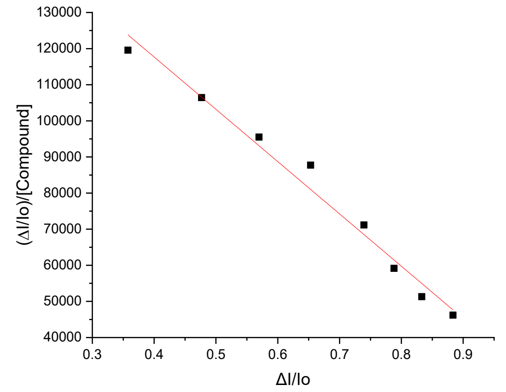

Complex **4**

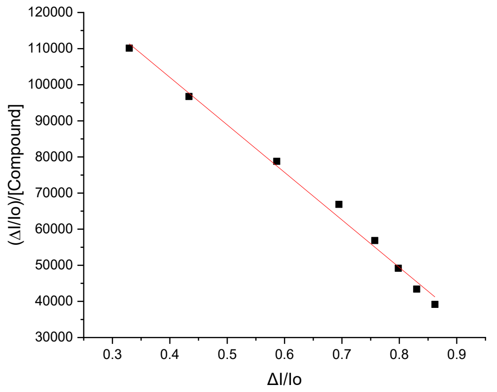

Complex **5**

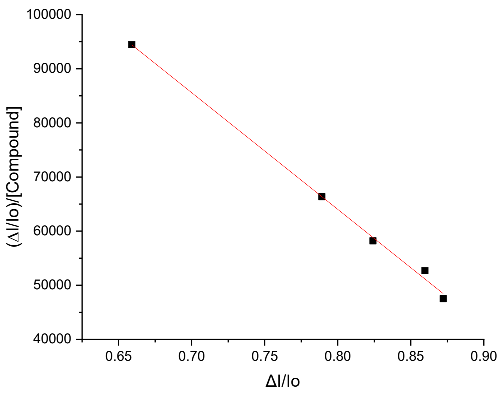

Complex **6**

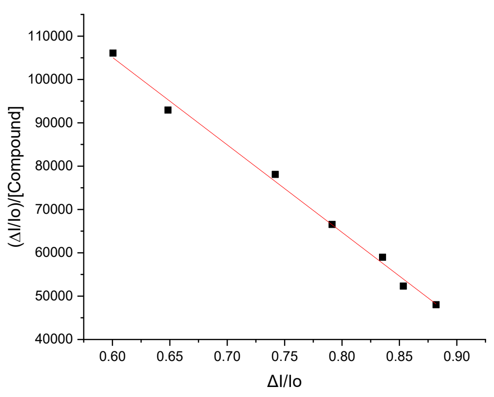

Complex **7**

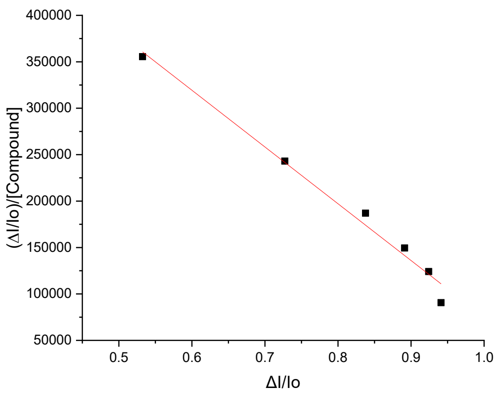

Complex **8**

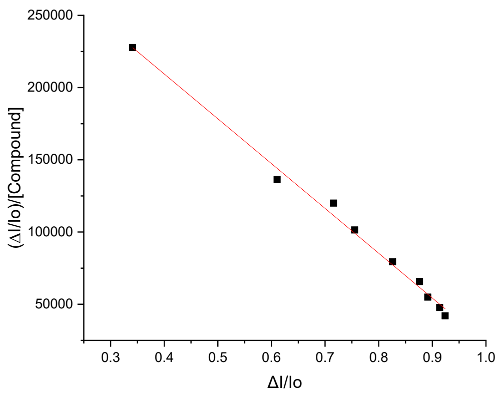

Complex **9**

Figure S30. Scatchard plots of the HSA quenching experiments in the presence of warfarin upon addition of the compounds.

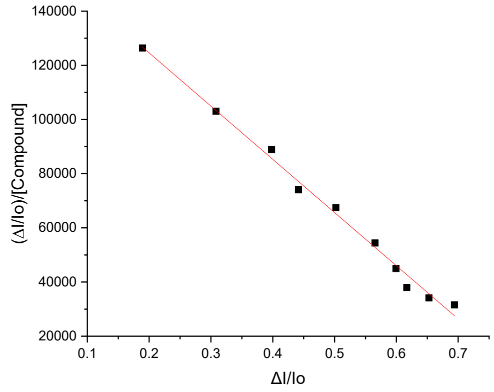

4′-Br-fenH

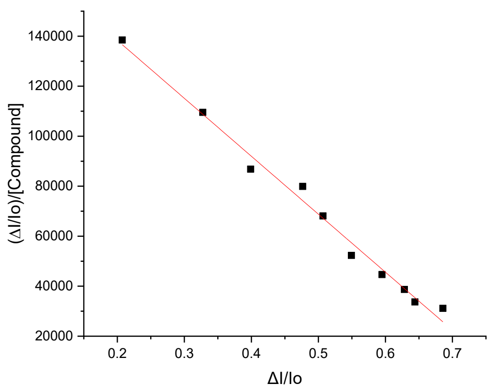

Complex **1**

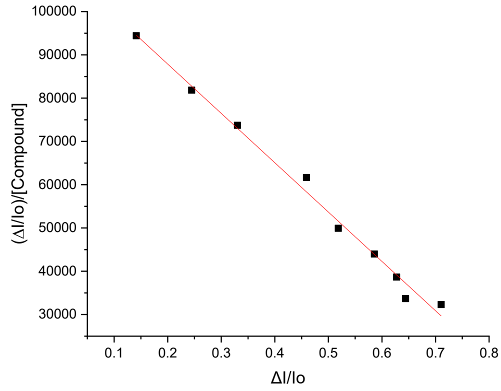

Complex **2**

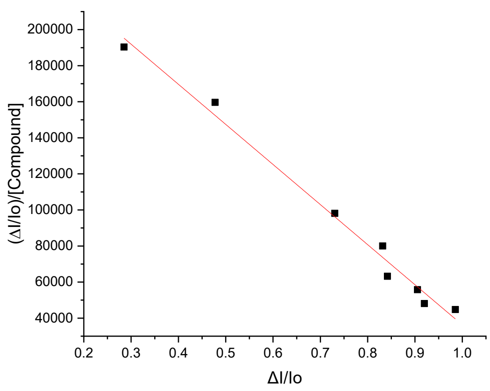

Complex **3**

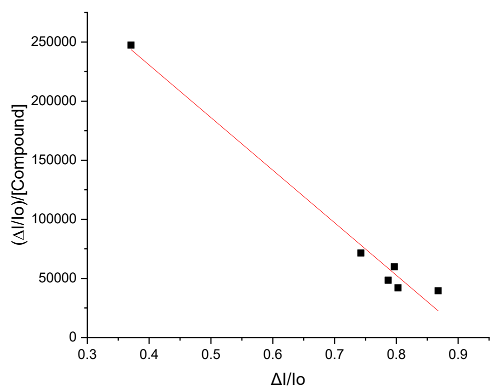

Complex **4**

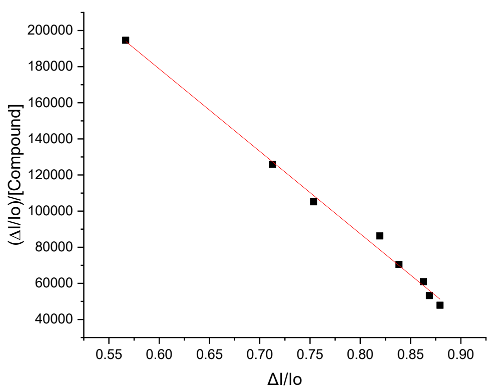

Complex **5**

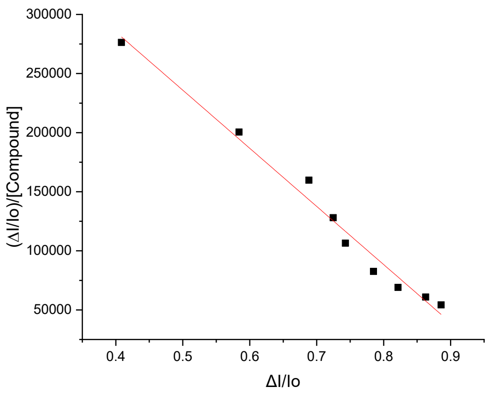

Complex **6**

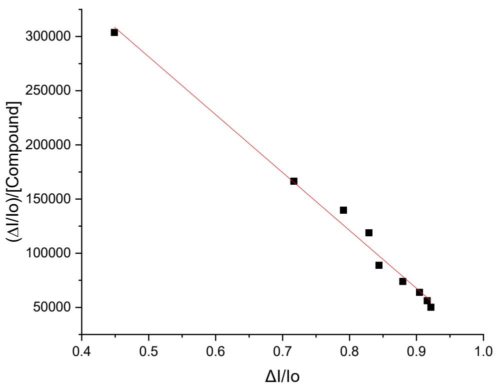

Complex **7**

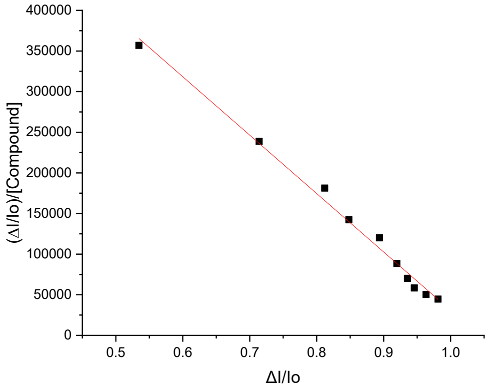

Complex **8**

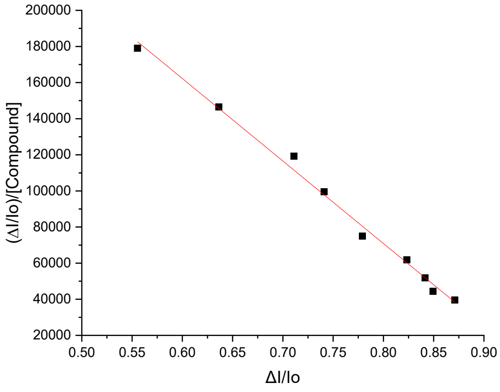

Complex **9**

Figure S31. Scatchard plots of the HSA quenching experiments in the presence of ibuprofen upon addition of the compounds.

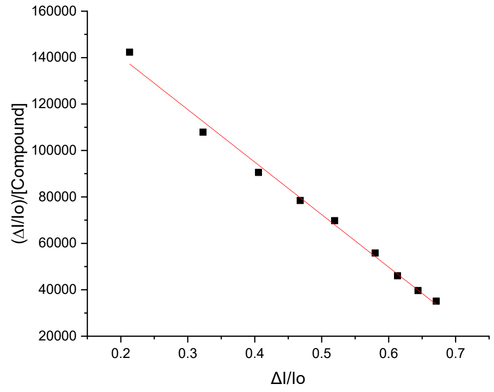

4′-Br-fenH

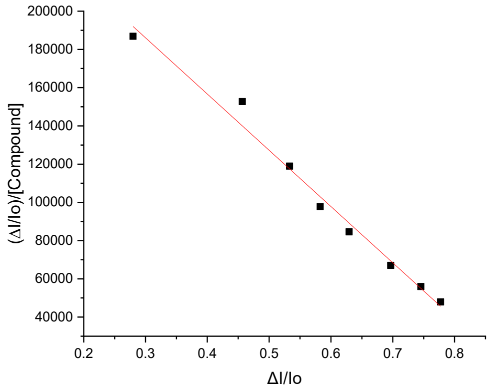

Complex **1**

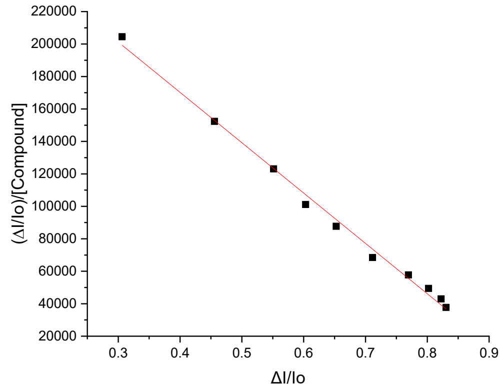

Complex **2**

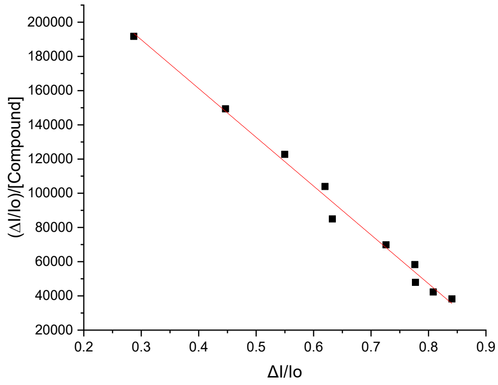

Complex **3**

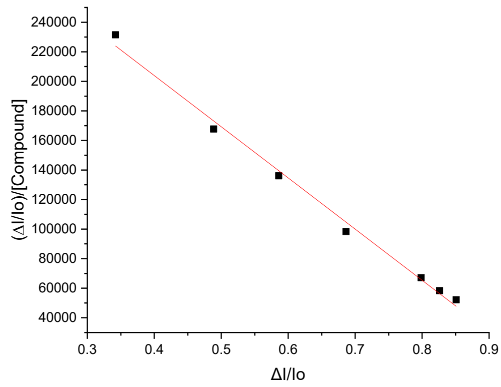

Complex **4**

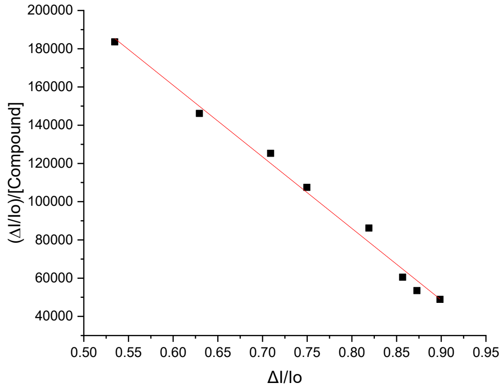

Complex **5**

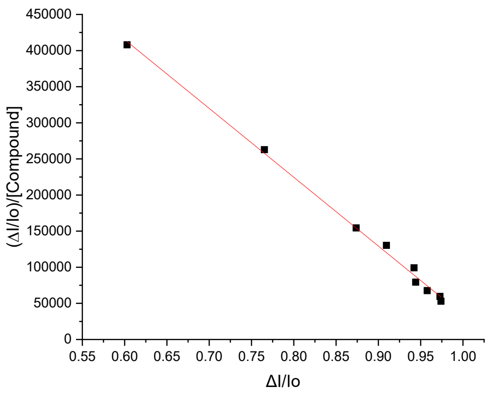

Complex **6**

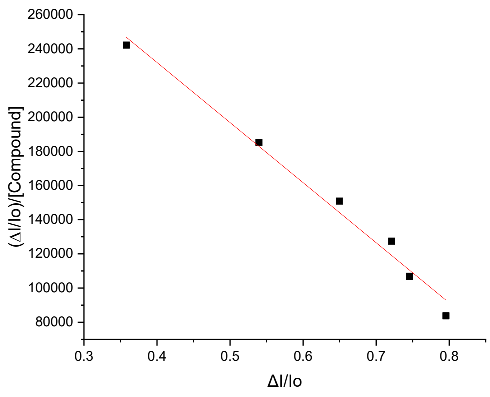

Complex **7**

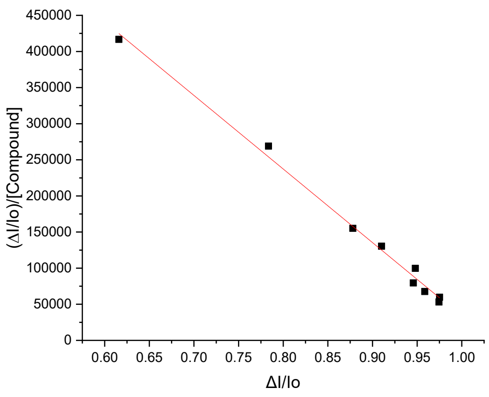

Complex **8**

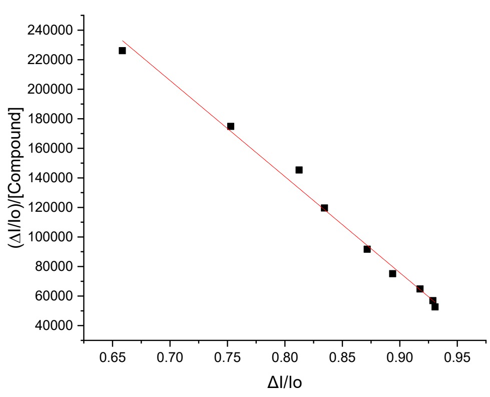

Complex **9**
